# Opposing activities of oncogenic MIR17HG and tumor suppressive MIR100HG clusters and their gene targets regulate replicative senescence in human adult stem cells

**DOI:** 10.1038/s41514-017-0006-y

**Published:** 2017-04-20

**Authors:** Mary F. Lopez, Ping Niu, Lu Wang, Maryann Vogelsang, Meenakshi Gaur, Bryan Krastins, Yueqiang Zhao, Aibek Smagul, Aliya Nussupbekova, Aikan A. Akanov, I. King Jordan, Victoria V. Lunyak

**Affiliations:** 1 Nuclea Biotechnologies, Cambridge, MA 02140 UK; 2 Department of Pediatrics, Renmin Hospital of Wuhan University, Wuhan, 430060 China; 3 Aelan Cell Technology, San Francisco, CA 92107 USA; 4 School of Biology, Georgia Institute of Technology, Atlanta, GA 30306 USA; 5 Department of Plastic Surgery, Renmin Hospital of Wuhan University, Wuhan, 430060 China; 6 Kazakh Medical University, Almaty, Kazakhstan; 7 PanAmerican Bioinformatics Institute, Cali, Valle del Cauca Colombia

## Abstract

Growing evidence suggests that many diseases of aging, including diseases associated with robust changes and adipose deports, may be caused by resident adult stem cell exhaustion due to the process called cellular senescence. Understanding how microRNA pathways can regulate cellular senescence is crucial for the development of novel diagnostic and therapeutic strategies to combat these pathologies. Herein, using integrated transcriptomic and semi-quantitative proteomic analysis, we provide a system level view of the regulation of human adipose-derived stem cell senescence by a subset of mature microRNAs (termed senescence-associated-microRNAs) produced by biogenesis of oncogenic *MIR17HG* and tumor-suppressive *MIR100HG* clusters. We demonstrate functional significance of these mature senescence-associated-microRNAs in the process of replicative senescence of human adipose-derived stem cells ex-vivo and define a set of senescence-associated-microRNA gene targets that are able to elicit, modulate and, most importantly, balance intimate connections between oncogenic and senescent events.

## Stem cells aging: balancing self-renewal halt with complexity

Mounting evidence suggests a link between cellular senescence and human adult stem cell function upon aging. Senescence is often viewed as an intrinsic program to prevent oncogenic transformation. However, the collaborative research lead by Aelan Cell Technologies suggests that replicative senescence might be more dynamic than previously anticipated. Integrated genomic and proteomic analyses of human adult stem cells revealed that subset of senescence-associated miRNAs (SA-miRNA) functionally required for establishing senescence, act to provide an intricate balance between driving and restraining cancerous events upon senescence. It is commonly believed that cancer arises as a consequence of an imbalance in cellular homeostasis. These new results shed light on the fundamental role of these SA-miRNA as functionally antagonistic regulators of gene networks, future exploration of which can help us understand the etiology underlying age-related disease and cancer.

## Introduction

Adipose tissue is not only a storage depot for triglycerides but also a critical functional organ regulating energy homeostasis. It is well known that abnormal fat accumulation and function is associated with adverse health outcomes, including obesity, type II diabetes, cardiovascular and cerebrovascular diseases and ultimately, aging. During adult adipose tissue homeostasis and turnover (tissue maintenance), adipocytes are derived from adipose tissue stem cells (ADSCs), whose origin has been traced to mural cells (also termed pericytes) residing in the perivascular niche via a specialized cell lineage differentiation process.^1–3^ ADSCs are a type of adult stem cell of mesenchymal origin that possess many of the traits common to bone marrow-derived mesenchymal stem cells (BMMSCs). New adipocyte formation is critical for adult homeostatic balance, and adipose tissue maintenance often requires a steady replenishment of cells from stem or progenitor sources.^4, 5^ However, throughout life it appears that changes in the quantity and quality of ADSCs due to external stimuli, specialized stem cell microenvironment, and/or intrinsic stem cell aging processes, can influence adipose tissue metabolism, turnover rate and regeneration and, surprisingly, also impose restrictions on ADSC immunomodulation properties invoked in settings such as tissue injury, transplantation and autoimmunity.^1, 2, 5–8^

Robust aging-related changes in tissue maintenance are thought to be caused by resident adult stem cell exhaustion due to the process called cellular senescence (SEN).^9–13^ SEN involves signaling, metabolic and cytoskeletal changes resulting in the diminished ability of cells to cope with DNA damage and to maintain the structure and function of chromatin.^2, 14^ Despite the effort to uncover crosstalk between cellular signaling pathways controlling SEN,^15, 16^ the full set of regulators involved in its establishment and maintenance are not well defined, and their complex interactions are still poorly understood.

Over the past decade, microRNAs (miRNAs) have emerged as a new dimension of sophisticated genomic regulations in a variety of physiological processes. Once the messenger RNA is targeted by miRNAs, the RNA-induced silencing complex is thought to inhibit protein production either through blocking translation or by reducing messenger RNA stability.^17–20^ A given miRNA can target a multitude of different mRNAs, and a given gene target might similarly be targeted by multiple miRNAs. For this reason, miRNAs frequently represent the central nodes of several regulatory networks and may act as rheostats to provide stability and/or fine-tuning to gene expression cascades.^21, 22^ Different miRNA expression profiles were reported for various cell types undergoing replicative SEN, such as arterial and umbilical vein-derived endothelial cells, replicating CD8(+) T cells, renal proximal tubular epithelial cells and skin fibroblasts,^23, 24^ indicating that miRNAs might play a major role in orchestrating replicative SEN.

Some miRNAs have been reported to accelerate or inhibit the process of adipocyte differentiation during adipogenesis, thereby influencing the process of cellular SEN as well as impacting the aging process in general.^25–29^ However, whether the altered miRNA profile is a consequence of SEN or whether it triggers replicative SEN is still a matter of debate. Although many methods have been proposed for miRNA target identification,^30, 31^ little is known about a specialized cohort of miRNA gene targets that can trigger and/or mediate senescent phenotypes and how downregulation in its expression is linked to restriction of proliferation capacity, diminished DNA damage repair and severe abnormality in the chromatin assembly generally observed upon SEN. Therefore, a better understanding of how miRNA pathways can regulate human adipose-derived stem cell (hADSC) SEN through their gene targets is crucial for the development of novel therapeutic strategies to combat the many diseases of aging, including diseases associated with robust changes and adipose depots.

In this manuscript, we investigate the critical role of two micro-RNA clusters, oncogenic *MIR17HG* and tumor-suppressive *MIR100HG,* in the process of replicative SEN of human adipose-derived stem cells (hADSCs) by using an integrated approach that combines RNA sequencing analysis (RNA-seq) and semi-quantitative proteomic analysis. Here, we uncover the complex interactions among several cellular processes biologically relevant for the state of cellular SEN. We provide functional evidence demonstrating that the senescent state of hADSCs is achieved by the combined action of SEN–associated miRNAs (SA-miRNAs), and we identify a set of novel gene targets that are susceptible to these miRNAs. Our data suggests a functional significance of these miRNAs in the complex SEN–associated changes within human adult adipose-derived stem cells.

## Results

### Replicative SEN of human adult ADSCs is associated with upregulation of a subset of non-coding RNA (ncRNAs)

**Fig. 1:**
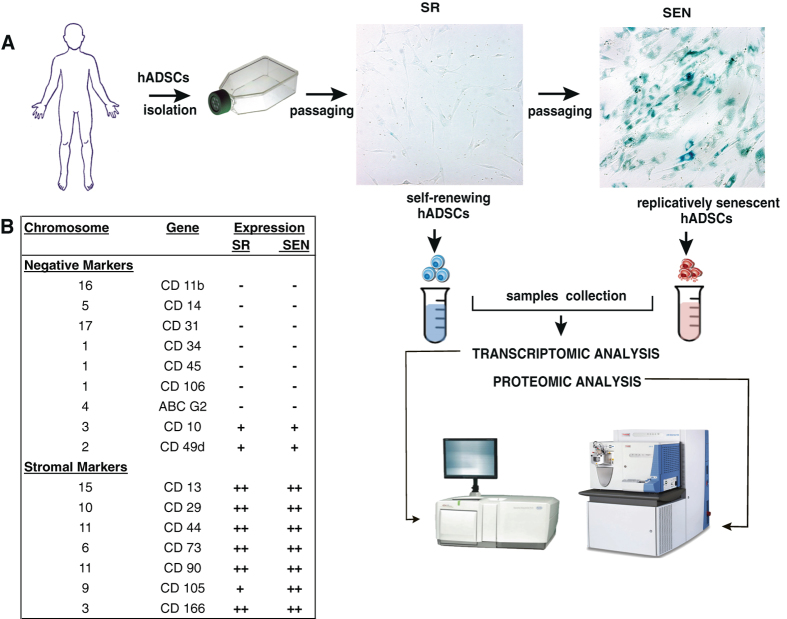
Schematic representation of sample collection for analysis and immunological stability of hADSCs upon replicative SEN. **a** hADSCs were isolated from healthy donor subcutaneous tissue and passaged ex-vivo as described in materials and methods. Colorimetric detection of SA-β-Gal (10x) in SR and SEN hADSCs is shown. Samples were collected and processed for transcriptomic and proteomic studies as described in material and methods. **b** The table summarizes immunostability of hADSCs in SR and senescent states, which were assessed by expression of MSC-positive and MSC-negative CD markers, details shown in Supplementary Table S3

Isolated hADSCs share many characteristics with BMMSCs, their counterparts in bone marrow.^32–34^ Similar to other types of MSCs, hADSCs are not considered to be immortal either in-vivo or ex-vivo. We and others have previously reported that isolated and ex-vivo cultured hADSCs exhibit consistent self-renewing (SR) and, upon approaching replicative SEN (Fig. 1 and Supplementary Figure S1A), cultures accumulate giant non-dividing cells, as determined by incorporation bromodeoxyuridine (BrdU) into DNA (Supplementary Figure S1B), expressing the enzyme lysosomal pH6 SEN-associated β-galactosidase (SA-β-Gal) (Fig. 1a). SEN hADSCs manifest a loss of control for chromatin organization, activate a persistent DNA damage response (DDR) (Supplementary Figure S1B and C) and manifest robust changes in transcriptional activity.^13, 35, 36^ As hADSCs approached SEN, both mediators of DDR, phosphorylated form of histone variant H2AX (γH2AX),^37^ and p53 binding protein-1,^38^ form characteristic persistent DNA damage foci (Supplementary Figure S1B and C). The presence of these foci drastically increased from very rare in SR ADSCs, to almost 90% in hADSCs approaching SEN (hADSCs) triggered by activation of the p53/P21^*WAF1/Cip1*^ pathway (Supplementary Figure S1B).^13^ In corroboration with previous reports, cluster of differentiation (CD) antigen marker characterization revealed that SEN hADSCs robustly express stromal markers CD29, CD44, CD73, CD90, CD105 while staying negative for hematopoietic lineage markers CD31, CD34 and CD45^32^ (Fig. 1b, Supplementary Figure S2A and S2B), suggesting phenotypical stability of SEN hADSCs.

We further assessed the changes associated with replicative SEN via transcriptome analysis (RNA-*seq)* and matching high-resolution LC-MS/MS differential abundance proteome analysis, a technique that can measure global changes in relative protein abundance (Fig. 1a) as described in materials and methods.^39, 40^

**Fig. 2:**
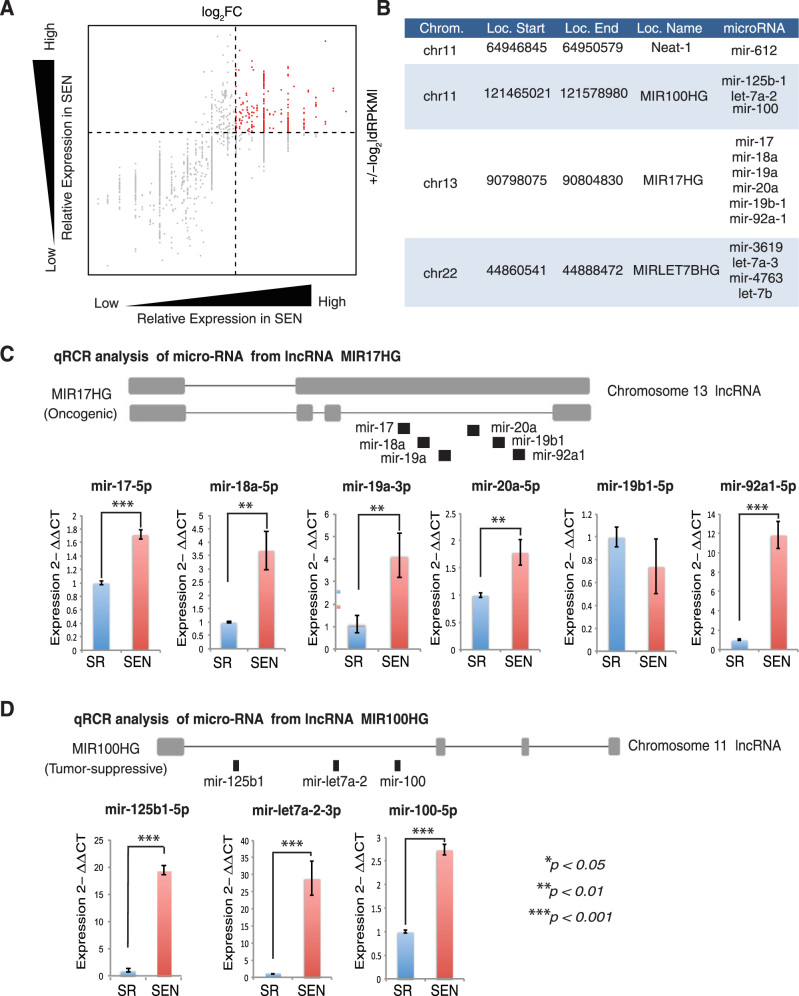
MiRNA clusters and SEN-associated miRNA (SA-miRNAs) discovered via RNA-seq analysis and experimentally validated by qPCR. **a** Differential expression of non-coding RNA genes in SR versus SEN hADSCs revealed by RNA-seq analysis. Fold-change values (log_2_ SEN/SR) are shown on the *x*-axis and RPKM differences (log_2_ SEN-SR) are shown on the *y*-axis. SEN upregulated non-coding RNA genes are shown in *red* (upper right quadrant). **b** Genomic locations and locus names for SEN upregulated miRNA gene clusters revealed by RNA-seq analysis. **c** Graphical representation of oncogenic MIR17HG locus and qPCR analysis of mature mirRNA expression in SR (*blue bar*) and senescent (SEN, *red bar*) states of hADSCs. Relative expression of either passenger strand mature miRNAs (depicted in the graphs as *-3p*) or guide strand mature miRNAs (depicted in the graphs as *-5p*) to U6 small RNA was measured. Data are shown as fold change (ΔΔC*τ*) The mean ± SD from three independent experiments is shown. The statistical difference was evaluated by Student’s *t-*test and *P-*value (*p*) related to experimental measurements and are listed under the graphs, where ***p < 0.001, **p < 0.01. **d** Graphical representation of tumor-suppressive MIR100HG locus and qPCR analysis of mature miRNA expression in SR (*blue bar*) and senescent (SEN, *red bar*) states of hADSCs. Relative expression of either passenger strand mature miRNAs (depicted in the graphs as *-3p*) or guide strand mature miRNAs (depicted in the graphs as *-5p*) to U6 small RNA was measured. Data are shown as fold change (ΔΔC*τ*) The mean ± SD from three independent experiments is shown. The statistical difference was evaluated by Student’s *t-*test and *P-*values (*p*) related to experimental measurements are listed under the graphs, where ***p *<* 0.001.

Comparative transcriptomic analysis (RNA-*seq*) between SR and SEN hADSCs revealed a number of ncRNAs that are upregulated in SEN compared to SR hADSCs (Fig. 2a and b, Supplementary Figure S3A, S3B and materials and methods). Differentially expressed ncRNAs are identified as those that have high levels of fold change (log_2_ SEN/SR) and significant differences in the normalized number of reads (dRPKM SEN-SR) as shown in Supplementary Figure S3B. The 216 ncRNAs upregulated upon SEN have been identified (shown in *red* in the upper right quadrant of Fig. 2a). Three out of four upregulated ncRNA loci (Fig. 2b) encode polycistronic transcripts that could be processed to yield multiple miRNAs (Fig. 2b): chr11:*MIR100HG* (encoding mir-125b1, mir-let7a-2, mir-100), chr13: *MIR17HG* (encoding mir-17, mir-18a, mir-19a, mir-20a, mir-19b-1, mir-92a-1) and chr22: *MIRLET7BHG* (encoding mir-3619, mir-let7a-3, mir-4763, mir-let-7b).

Numerous studies have been done on these ncRNA loci, reporting the potent phenotypes induced by their genetic perturbations and by overexpression/deletion of these loci-associated miRNAs, thus placing these genetic regions at the center of numerous cellular and developmental pathways.^41–43^ *MIR17HG* and *MIR100HG* clusters and their paralogs have been placed at the nexus of opposing activities during malignancy development: oncogenic^41, 44^ and tumor-suppressive,^42, 45, 46^ respectively. Similarly, the *MIRLET7BGH* cluster has been identified as a potent tumor suppressor and a regulator of cellular SEN, functionally linked to neural stem cell age-related decline through the regulation of *p16(Ink4a)*.^43, 47–49^

### Dynamic and differential expression of mature miRNAs from MIR17HG and MIR100HG clusters upon replicative SEN of hADSCs

In the context of this study, we further focused on upregulation of functionally antagonistic *MIR17HG* and *MIR100HG* miRNA-bearing loci upon senescence. The human chromosome 13 *MIR17HG* cluster (800bp) encodes six tightly grouped miRNAs with four distinct “seed” sequences^31, 50^: mir-17, mir-18a, mir-19a, mir-20a, mir-19b1, and mir-92a1 (schematically shown in Fig. 2c). The miRNAs from this locus have been designated as onco-miRNAs because of their importance in cell transformation and tumorigenesis.^41, 46^ The chromosome 11 MIR100HG cluster homes three miRNAs (mir-125b1, mir-let7a-2, mir-100) situated within a comparable genomic distance (Fig. 2d).

The miRNAs are frequently transcribed together as polycistronic primary transcripts that are processed into multiple individual mature miRNAs.^51^ To identify specific miRNA production from these clusters in SEN hADSCs, we examined the abundance of mature miRNAs originating from both guide strand (mir-*5p*) and passenger strand (mir-*3p*/ mir*) by the MystiCq miRNA qPCR assay system as described in materials and methods.

Analysis of the *MIR17HG* cluster has revealed that only mature guide strand miRNAs: mir-17-*5p*, mir-18a-*5p*, mir-20a-*5p*, mir-19b1-*5p* and mir-92a1-*5p*, are detected in both SR and SEN hADSCs (Fig. 2c). No mature passenger strands: mir-17-*3p*, miR-18a-*3p*, miR-20a-*3p*, mir-19b1-*3p* and mir-92a1-*3p*, have been observed in the tested samples. Contrary to that, only mature passenger strand miRNA for mir-19a-*3p* is robustly recorded by real-time PCR (Fig. 2c). We have observed a statistically significant SEN-related increase in production of mature miRNAs in accordance with their corresponding primary non-coding transcripts *MIR17HG*: miR-17-*5p* (*p* < 0.001), miR-18a-*5p* (*p* < 0.01), miR-20a-*5p* (*p* *<* 0.01), mir-92a1-*5p* (*p* *<* 0.001) and mir-19a-*3p* (*p* *<* 0.01) (Fig. 2c and Supplementary Figure S4A). No significant change in the mature mir-19b1-*5p* has been detected upon replicative SEN of hADSCs (Fig. 2c).

The *MIR100HG* cluster has given rise to two guide strand mature miRNAs: mir-125b1-*5p* and mir-100-*5p* in SEN hADSCs. No mature passenger strands: mir-125b1-*3p* and mir-100-*3p* have been detected in our samples. Contrary to this notion, both guide mir-let7a-2-*5p* and passenger mir-let7a-2-*3p* have been detected in both SR and SEN conditions, where the balance in the stability/maturation preference of guide mir-let7a-2-*5p* is shifted upon SEN, favoring the production of passenger strand mir-let7a-2-*3p* (greater than 25-fold upregulation shown in Fig. 2c and Supplementary Figure S3A). Interestingly, this switch in the mature strand selection for mir-let7a-2 in SEN hADSCs is not due to an increase in AGO3 protein expression as was reported previously.^52^ The level of endogenous AGO3 protein does not seem to change significantly with replicative SEN (Supplementary Figure S4B).

Together, these data provide evidence that SEN of hADSCs correlates with a dramatic upregulation of the subset of mature miRNAs from the *MIR100HG* and *MIR17HG* clusters, and for some of them, such as mir-let7a-2, a notable shift in the maturation equilibrium between guide and passenger strands of miRNA has been observed. These abundantly upregulated mature miRNAs are called herein: SEN-associated micro RNAs (SA-miRNAs).

### Identification of the gene targets of SA-miRNAs by combined transcriptome and proteome analysis

Despite a plethora of available miRNA target prediction algorithms, it remains a challenge to predict the potential target genes of a given miRNA. A number of these prediction algorithms use sequence, contextual, structural and/or evolutionary constraints and rely on subsequent validation of the targets by assessment of mRNA expression level at the large scale.^53^ However, transcriptional analysis of miRNA target genes does not fully reveal the extent to which miRNAs can exert control on protein expression levels, which have a tendency to change more dramatically than mRNA levels.^31, 54, 55^

**Fig. 3:**
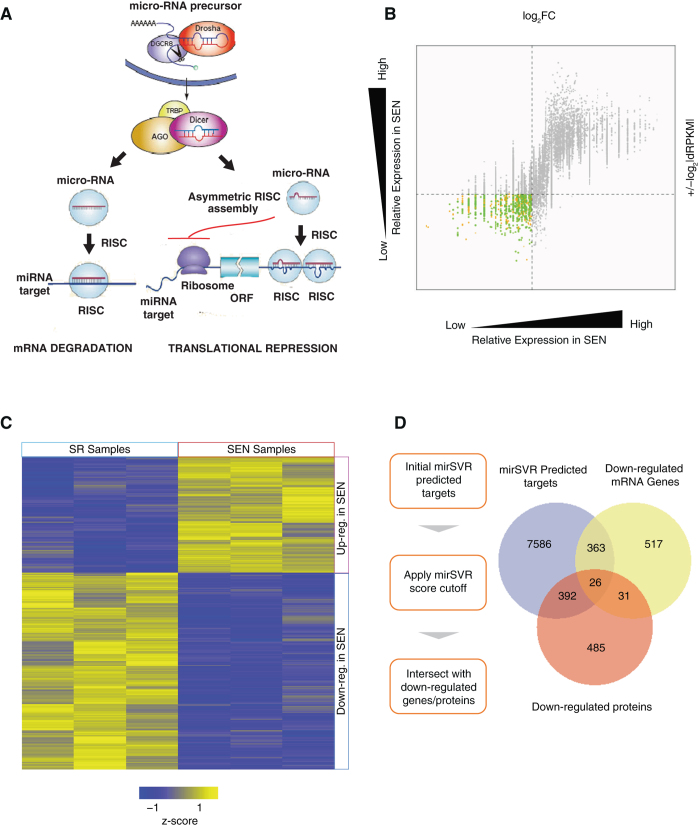
Differential expression of mRNA and protein targets of SA-miRNAs. **a** Schematic showing the mRNA degradation versus translational repression modes of miRNA regulation. **b** Differential expression of protein-coding mRNAs in SR versus SEN hADSCs. Fold-change values (log_2_ SEN/SR) are shown on the *x*-axis, and RPKM differences (log_2_ SEN-SR) are shown on the *y*-axis. SEN downregulated protein-coding mRNAs are shown in *green* (lower left quadrant). **c** Heatmap depicting differential expression of proteins in SR versus SEN hADSCs. Normalized protein expression levels are shown for three replicate samples each of SR versus SEN hADSCs (see *z-score* color scale). SEN downregulated proteins are shown in the lower right quadrant. **d** A flowchart illustrating the approach to identifying downregulated SA-miRNA targets is shown along with a Venn-diagram indicating the numbers of genes or proteins identified via each method and the numbers identified by multiple methods

Next, we have undertaken an integrated approach (illustrated in Fig. 1, Supplementary Figure S3C, S5 and described in materials and methods) to simultaneously explore two mechanisms by which SA-miRNAs might exert their functional effects: (1) mRNA degradation (Fig. 3a-left side of the cartoon), and (2) inhibition of protein translation without triggering mRNA decline (translational repression; Fig. 3a-right side of the cartoon).

Since we wished to relate SA-miRNAs to the downregulation of their target genes at the level of mRNA and/or protein expression, we have focused our analysis on SEN-downregulated mRNAs and proteins. SEN-downregulated mRNAs are characterized as those that have low levels of fold change (log_2_ SEN/SR) and the smallest values for the difference in the normalized number of reads (dRPKM SEN-SR). There are a total of 937 SEN-downregulated mRNAs that have been identified in this way (shown in *green* in the lower left quadrant of Fig. 3b). SEN-downregulated proteins have been identified by comparing protein expression levels across SR versus SEN replicate samples. There are 986 proteins that have shown significantly lower levels of expression among SEN replicates compared to SR replicates (shown in *blue* in the lower right quadrant of Fig. 3c).

Having identified SEN downregulated mRNAs and proteins in this way, we then applied the mirSVR prediction algorithm (Supplementary Figure S6) to search for potential target genes of SA-miRNAs (Fig. 2c and d) among these mRNAs and proteins (materials and methods). The mirSVR algorithm has been chosen because it combines multiple sources of information for miRNA target prediction, including gene expression data from miRNA transfection experiments, thereby allowing for a lower rate of false positive predictions (Supplementary Methods). We have undertaken a conservative approach to miRNA prediction by only selecting targets with a score <−0.2. Among 8367 targets predicted by mirSVR, 389 mRNAs, and 418 proteins have been downregulated upon SEN of hADSCs (shown as Venn diagram in Fig. 3d).

Collectively, the SA-miRNA target genes captured by this approach may represent numerous biological pathways relevant to the establishment and/or maintenance of the SEN phenotype pathways in hADSCs.

### Validity and sensitivity of the integrated transcriptome and proteome approach for the identification of SA-miRNA target genes

To verify the validity and sensitivity of our integrated approach for the identification of SA-miRNA target genes, we evaluated gene expression of two previously established targets of mature mir-100 from the *MIR100HG* locus, the *HOXA1* and *SMARCA5* genes.^42, 56^ Mir-100 directly targets these genes in mammary epithelial cells, imposing epithelial-to-mesenchymal transition (EMT) through downregulation of their expression (Supplementary Figure S7A). Consistent with published findings, our LC-MS/MS proteomic data demonstrates that the protein expression levels of both SMARCA5 and HOXA1 are significantly reduced upon SEN of hADSCs (Supplementary Figure S7B) in accordance with endogenous upregulation of *mir-100-5p* (Fig. 2d). Notably, both *SMARCA5* and *HOXA1* mRNA levels in SEN cells do not show significant downregulation when compared to SR cells, thus suggesting that *mir-100-5p* operates via the translational repression pathway illustrated in Fig. 3a (right panel). These findings provide a proof-of-principle that our approach is valid and reliable for deciphering targets of SA-miRNA action.

### Destabilization of mRNA and translational repression through SA-miRNAs upon SEN of hADSCs

**Fig. 4:**
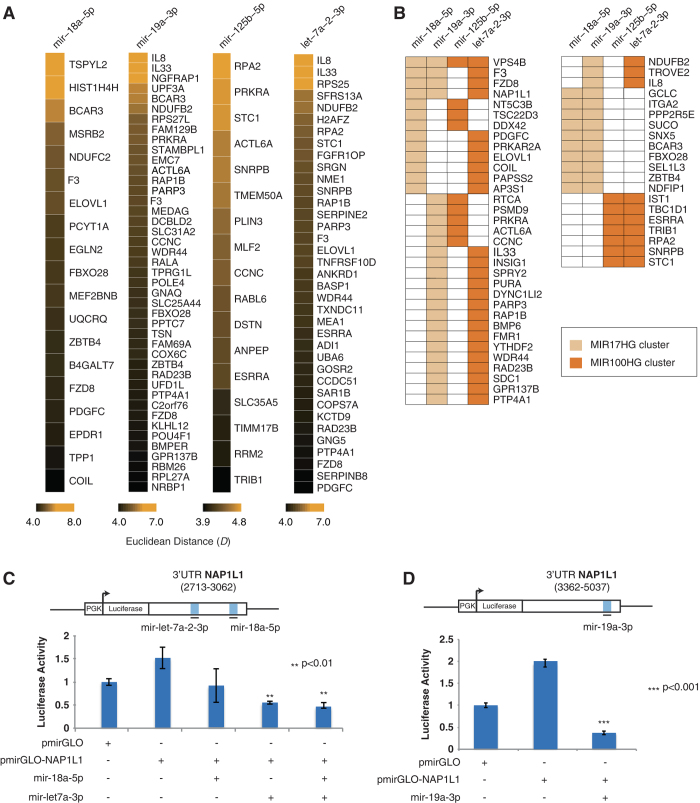
Downregulation of SA-miRNAs target genes via mRNA degradation in SEN. **a** SEN downregulated protein coding mRNAs targeted by SA-associated miRNAs. Differential expression levels are quantified by the Euclidean distance as described in the electronic supplementary materials and Supplementary Figure S2. **b** Coordinated regulation of SEN downregulated protein-coding mRNAs by multiple SA-miRNAs. Individual miRNAs are shown and color-coded according to their miRNA gene cluster. **c**, **d** Coordinated regulation of *NAP1L1* UTRs by SA-miRNAs. Schematic diagrams of predicted target sites of SA-miRNAs in the two distal portions of *NAP1L1* UTRs: A portion from 2713 to 3062 relative to the transcriptional start site TSS (**c**) and a portion from 3362 to 5037 relative to the TSS (**d**). Repression of luciferase reporters bearing the UTRs (pmirGLO-*NAP1L1)* and corresponding control luciferase vector pmirGLO by mimic SA-miRNAs (*n* = 3, mean = +/−SD, two-tailed, type 2, Student *t*-test, compared to the control vector pmirGLO). *P-*value (*p*) related to experimental measurements are listed over the graphs, where ***p *<* 0.001, **p < 0.01

Our transcriptome analysis has revealed 389 downregulated mRNA representing SEN-associated degradation targets of SA-miRNAs in hADSCs (Fig. 3d). Representative heatmaps of transcriptional changes of SA-miRNA mRNA targets for individual SA-miRNA are shown in Fig. 4a and Supplementary Figure S8A, and their expression levels are shown in Supplementary Table S1.

**Fig. 5:**
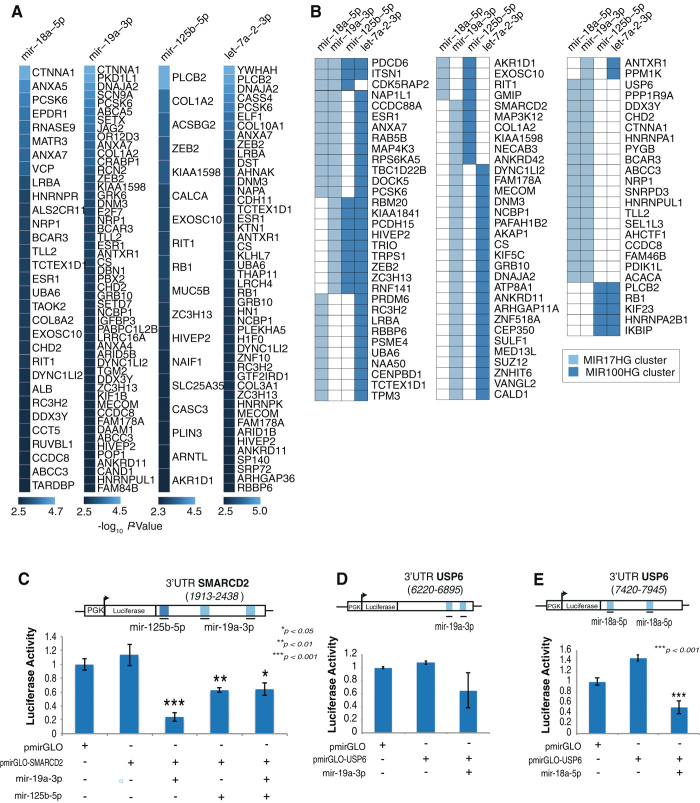
Downregulation of proteins *via* SA-miRNA-based translational repression in SEN. **a** SEN downregulated proteins targeted by SEN upregulated miRNAs. Differential protein expression is quantified by the Students’ *t-*test (-log_10_ *P*-values shown) as described in the electronic supplementary materials. **b** Coordinated regulation of SEN downregulated proteins by multiple SA-associated miRNAs. Individual miRNAs are shown and color-coded according to their miRNA gene cluster. **c** Coordinated regulation of *SMARCD2* UTR by SA-miRNAs. Schematic diagrams of the predicted target sites of SA-miRNAs in the (1913-2438) portion of *SMARCD2* UTR. **d**, **e** Coordinated regulation of *USP6* UTRs by SA-miRNAs. Schematic diagrams of the predicted target sites of SA-miRNAs in the two distal portions of *USP6* UTRs: a portion from 6220 to 6895 relatively to the transcriptional start site TSS (**d**) and a portion from 7420 to 7945 relatively to the TSS (**e**). Repression of luciferase reporters bearing the UTRs (pmirGLO-*SMARCD2* and pmirGLO-*USP6,*) and corresponding control luciferase vector pmirGLO by mimic SA-miRNAs (*n* = 3, mean = +/−SD, two-tailed, type 2, Student *t*-test, compared to the control vector pmirGLO). *P-*value (*p*) related to experimental measurements are listed over the graphs, where ***p < 0.001, **p < 0.01, *p < 0.05.

Similarly, 418 out of 8367 targets predicted by mirSVR targets have shown evidence of downregulation at the level of protein expression based on the results of the LC-MS/MS proteomic analysis (Figs. 3d and 5). The heatmaps of the representative targets of the individual SA-miRNAs are shown in Fig. 5a, Supplementary S9A and detailed in Supplementary Table S2.

Interestingly, both downregulated mRNA (Fig. 4a) and numerous SA-miRNA targets associated with translation repression have shown a trend towards a potential co-regulation by two or more of the SA-mRNAs (Figs. 4b and 5b, Supplementary S8B and S9B). These results, in accordance with previously reported data, suggest that under physiological conditions many of the SA-miRNA target genes might be subjected to concurrent regulation by multiple co-expressing miRNAs from clusters with opposing biological roles: oncogenic versus tumor-suppressive.^31, 57^ This observation raises the following question: what function does the targeting of mRNA by multiple miRNAs from the same cluster or from clusters with opposing biological activity serve?

### Combinatorial regulation of SA-miRNA gene targets NAP1L1, SMARCD2 and USP6 by the miRNA from oncogenic MIR17HG and tumor-suppressive MIR100HG clusters

To answer this question, we tested the hypothesis that co-expression of multiple miRNAs induces stronger downregulation of their common targets. We have focused on three SA-miRNA target genes that exemplify the following co-targeting arrangements: (1) a critical chromatin chaperone, NAP1L1^58^ targeted by multiple miRNAs from antagonistic *MIG17HG* and *MIR100HG* clusters (Fig. 4b), (2) a component of the chromatin remodeling complex, barrier to autointegration factor complex, SMARCD2/BAF60B^59, 60^ targeted by a single miRNA from antagonistic clusters (Fig. 5b), (3) a potent oncogene, USP6/TRE17^61, 62^ targeted by multiple miRNAs with two distinct “seed” sequences from the same cluster (Fig. 5b).

To demonstrate the regulatory effects of *mir-let-7a-5p, 18a-5p and mir-19a-3p, mir-19a-5p* on NAP1L1, SMARCD2 and USP6 expression we performed in vitro luciferase assays (materials and methods). For this purpose, corresponding 3′UTRs of: (1) *NAP1L1* (3′UTR 2713-3062 and a portion of 3′UTR 3362-5037) shown in Fig. 4c and d, (2) *USP6* (portions of 3′UTR 6220-6895 and 3′UTR 7420-7945*)* shown in Fig. 5d and e, and (3) 3′UTR 1913-2438 of *SMARCD2* (Fig. 5c) genes were cloned into the pGL3-promoter vector, immediately downstream of the luciferase gene. These reporter constructs were transfected into 293T cells lacking endogenous expression of mature *mir-let-7p-2-3p*, *mir-18a-5p, mir-19a-3p or mir-125b-5p* miRNAs, either alone or in combination with synthetic small, double-stranded RNA molecules designed to mimic endogenous mature miRNA molecules, *mimic* miRNA (Sigma, St. Louis, MO), as previously described.^63, 64^ To validate our hypothesis the mimic miRNAs were transfected either alone or in combinations as described in materials and methods and shown in Figs. 4c, d and 5c, d, e.

We observed that luciferase activity in cells transfected with pGL3-*NAP1L1*-3′UTR was significantly reduced as compared with cells transfected with the control pGL3 vector only by *mir-let-7a-5p (47%)* and *mir-19a-3p (81%)* as shown in Fig. 4c and d, respectively. No significant downregulation of luciferase activeity of pGL3-*NAP1L1*-3′UTR was observed when the *mir-18a-5p* mimic was used (Fig. 4c); although, the *mir-18a-5p* mimic can efficiently downregulate luciferase activity of pGL3-*USP6* -3′UTR (65%) in similar experiments shown in Fig. 5e. This suggests that *NAP1L1* is efficiently targeted by *mir-let-7a-3p* and *mir-19a-3p*, but not *mir-18a-5p*, which originates from the same cluster, *MIR17HG*. Analysis of pGL3-*SMARCD2*-3′UTR revealed a similar trend and confirmed that *SMARCD2* is a target of two SA-miRNAs: *mir-19a-3p* and *mir-125b-5p* (Fig. 5c).

In a similar experiment, we tested two composite 3′UTR parts of the *USP6* gene (Fig. 5d and e). The 3′UTR of the *USP6* gene responded with statistical significance to only one miRNA from the *MIR17HG* cluster. Transient transfection of the mimic of *mir-18a-5p* resulted in a 65% downregulation of luciferase activity (Fig. 5e), while transfection of the mimic of *mir-19a-3p* showed no significant change (Fig. 5d). These findings argue for the preferential use of SA-miRNAs originating from the same cluster for the concurrent regulation of the same genes.

We also tested the ability of pairs of miRNAs to synergistically regulate mutual targets in order to facilitate more efficient target repression, a phenomenon known as cooperating miRNAs.^31^ Although each single SA-miRNA efficiently downregulated the *NAP1L1*, *SMARCD2* and *USP6* UTRs in transient transfection experiments (Figs. 4c and d, 5c and d), data has shown that simultaneous transfection of multiple micro-RNA mimics targeting the same UTR does not increase the efficiency of target downregulation in all of the tested reporter assay combinations. This argues against the implicit assumption that a stronger downregulation of common gene targets could be achieved by multiple simultaneously co-expressing miRNAs, thus leading to a larger response of the target to miRNA perturbation.

These data suggest that the concept of miRNA cooperativity might imply a much more sophisticated mechanism of regulation of miRNA targets than was initially anticipated. For example, selective, physiologically-relevant expression of cooperating miRNAs could be adopted by cells to facilitate distinctive and fine-tuned gene expression patterns to meet the requirements of different biological scenarios and this phenomenon is unlikely to be appropriately tested in transient transfection experiments.

### Network-based functional enrichment analysis of SA-miRNA targets

Since clustered SA-miRNAs are co-expressed at different levels upon SEN in hADSCs (Fig. 2c and d), one might expect that they jointly regulate distinct molecular pathways not only by co-targeting individual genes but also by targeting differential components of the same pathways as previously shown for *Drosophila melanogaster*.^44, 65^ With this in mind, we developed a network-based functional enrichment analysis method to visually elucidate the potential roles of, and interactions among, integrated molecular networks of functionally related gene targets of SA-miRNAs in hADSCs (see materials and methods for details on the network analysis).

**Fig. 6:**
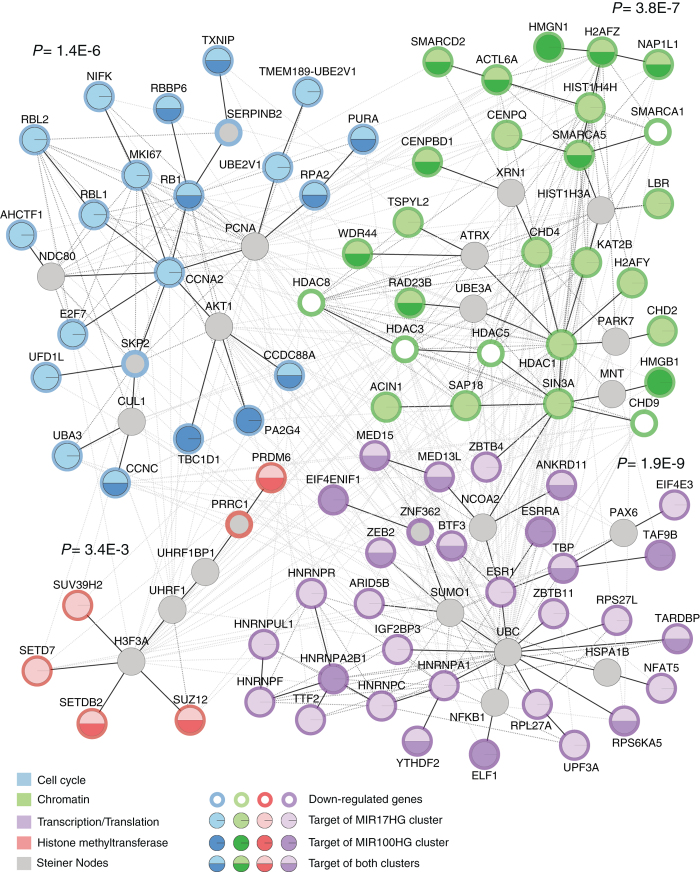
Functional relationships and enrichment of SA-miRNAs targets genes. Four functional categories of genes were evaluated for their relationships and functional enrichment using a network-based approach as described in the experimental procedures. The network nodes represent genes and are color-coded based on their functional category. The color key shows function. Gene nodes are labeled in regards to miRNA seed presence (see node label key in the Figure). Edges represent annotated protein relationships from the STRING database. *Black solid* edges represent connections of the sub-network minimal spanning trees (*i.e.* Steiner trees), *dark gray dashed* edges show additional sub-network connections, and *light gray dashed* edges represent connections between function-specific sub-networks. *P*-values indicate the extent to which each function-specific sub-network is enriched for genes from that particular functional category. Steiner nodes are shown in *gray*. Downregulated genes, which are not targeted by SA-miRNAs are shown with colored rim based on their functional category.

SA-miRNA targets that were found to be downregulated at the mRNA (Fig. 4 and Table S1) or protein level (Fig. 5 and Supplementary Table S2), were interrogated based on their functional annotations and used to seed the network analysis.^66^ Four categories of particular interest have been identified as relevant to the establishment and maintenance of the senescent phenotype: cell cycle, chromatin, transcription/translation and histone methyltransferases. To identify functional interactions among the corresponding SA-miRNA gene targets from these four categories, we then linked these genes in a network by edges that represent known relationships between the genes based on a variety of functional interactions, such as physical protein-protein interactions, gene co-expression and text mining co-relationships. Genes that do not have any direct known relationships of this kind are transitively linked via the minimum number of possible intermediate gene nodes, some of which are not targets of SA-miRNAs but have been downregulated in SEN hADSCs (Fig. 6 open *circles*). The intermediate nodes, which were not initially identified as miRNA targets or downregulated upon SEN, are the so-called Steiner nodes shown in *gray* in Fig. 6 and described in materials and methods.

The network functional enrichment analysis resulted in the elucidation of four clearly defined function-specific sub-networks, each of which corresponds to a distinct functional category, along with the inter-relationships between these functional groups (Fig. 6). The coalescence of genes with the same function into discrete sub-networks supports their close functional relationships and tight interactions, and the statistical significance of the functional enrichment within these groups is represented by *P*-values (Fig. 6) determined via simulation of random Steiner networks with the same number of genes from that particular functional category as described in experimental procedures. The *P*-values represent the probability of reconstructing sub-networks of the observed sizes, or smaller, by chance; in other words, they provide significance levels for the observed functional coherence of the sub-network.

Considering that we started with less than 1% of the total human genes, the large network of functionally related clustered genes identified by this method is surprising but immediately apparent. These data indicate that SA-miRNAs jointly regulate molecular pathways not only by co-targeting individual genes but also by targeting different components of the pathways that interconnect and could be relevant to SEN of hADSCs.

### Functional significance of SA-miRNAs from the *MIR17HG* and *MIR100HG* clusters in establishment of the senescent phenotype in hADSCs

**Fig. 7:**
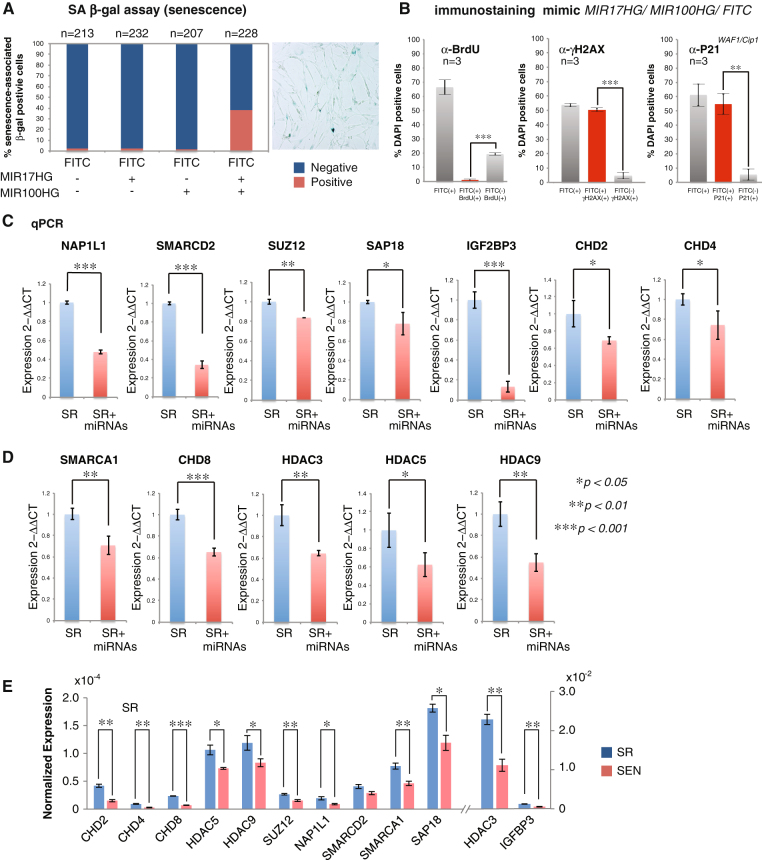
SA-miRNAs from oncogenic *MIR17HG* and tumor-suppressive *MIR100HG* clusters function to establish senescent phenotype in hADSCs. **a** Percentage of SA-β-Gal positive cells among the total amount of cells counted after transient transfection of the mimics of the SA-miRNAs from either the *MIR17HG* (*mir-17-5p*, *mir-18a-5p*, *mir-19a-3p*, *mir-20a-5p* and *mir-92a1-5p*) or the *MIR100HG* (*mir-125b1-5p*, *mir-1let7a-2-3p*, *mir-100-5p*) clusters separately or after simultaneous transfection by a full set of the SA-miRNA mimics from both clusters in SR hADSCs. SA-miRNA mimics were transfected with FITC-labeled control to account for the transfection efficiency as described in material and methods and electronic supplementary materials. The transfection efficiency for each combination is shown in (Supplementary Figure S8D) and is expressed as a percentage of green cells among the total DAPI-positive cells (*n*) counted under the fluorescent microscope. **b** Quantitation of the BrdU incorporation in SA-miRNA mimics-transfected SR hADSCs (FITC+) 72 hrs post-transfection presented as FITC+ BrdU+ (*red bar*). Quantification of cells positive for SEN-associated markers P21^*WAF1/Cip1*^ and γH2AX persistent DDR focal staining is performed under the same conditions in FITC+ (*red bars*) and FITC− cells. Results were plotted on the graphs as the averages of three independent experiments (biological replicates *n* = 3) with the standard deviation of data. Cell counted in each experiments: BrdU staining *n*_*1*_ = 440, *n*_*2*_ = 279, *n*_*3*_ = 334; γH2AX staining *n*_*1*_ = 117, *n*_*2*_ = 109, *n*_*3*_ = 133; P21^*WAF1/Cip1*^ staining *n*_*1*_ = 118, *n*_*2*_ = 135, *n*_*3*_ = 90. *P*-values *(p)* were calculated as Students’ two-tailed test: BrdU labeling experiment ***p < 0.001; γH2AX staining experiment ***p *<* 0.001; P21^*WAF1/Cip1*^ staining **p *<* 0.01. **c** The panels demonstrate direct influences of SA-miRNAs on gene transcription. Expression of the direct SA-miRNA target genes was measured by qPCR analysis in SR hADSCs (SR, *blue bars*) and SR cells transiently transfected with a full set of the SA-miRNA mimics (SR + miRNA, *red bars*). **d** The panel demonstrates indirect influences of the SA-miRNAs on gene transcription. Expression of genes previously shown to be downregulated in replicative senescent hADSCs but not identified as SA-miRNA targets was measured by qPCR analysis in SR hADSC cells (SR, *blue bars*) and in SR cells transiently transfected with the full set of SA-miRNA mimics (SR + miRNA, *red bars*). RNA was isolated from the cells 48hrs post-transfection. Samples were normalized against β-actin. Mean expression levels ± SEM (*n* = 3) are shown as fold change (ΔΔC*τ*). **e** Normalized mean protein expression levels ± SEM (*n* = 3) in SR (*blue bars*) and SEN (*red bars*) hADSCs are shown for SA-miRNA direct and indirect target genes. Statistical differences for the qPCR mRNA (**c**) and (**d**) and protein (**e**) expression comparisons were evaluated by Student’s *t*-test, where ***p *<* 0.0001, **p < 0.01, *p < 0.05.

To support our hypothesis that SA-miRNAs originating from *MIR17HG* and *MIR100HG* clusters may coordinately regulate gene targets that play key functional roles in the transition of hADSCs from self-renewal to replicative SEN, we next investigated whether transient delivery of SA-miRNAs to SR ADSCs is sufficient to cause senescent phenotypes. Our data indicates that delivery of the miRNA mimics of SA-miRNAs from either *MIR100HG* or *MIR17HG* clusters separately does not result in a senescent phenotype as detected by SA-β-Gal (Fig. 7a). And are negative for additional SEN-associated markers such as immunostaining with anti-P21^*WAF1/Cip1*^ antibodies (Supplementary Figure S7F and G) and persistent SEN-associated DNA damage foci (γ−H2AX staining) (Supplementary Figure S7F and G). FITC-labeled random RNAs have been used as a control for the transfection efficiency in all of these experiments. The transfection efficiency ranges from 50 to 60% (Fig. 7d). When SA-miRNAs from both clusters were transfected into SR hADSCs, about 40% of the cells became marked by expression of SA-β-Gal (Fig. 7a), about 98% of FITC positive (FITC+) cells contained senescent-associated γ−H2AX (Fig. 7b and S1B and C), marked by proliferation arrest as detected P21^*WAF1/Cip1*^ staining and quantified by BrdU incorporation in FITC + cells (Fig. 7b and Supplementary Figure S1B). The senescent phenotype can be achieved by a range of concentrations (5pM or 10pM combined SA-miRNA mimics) widely used by others in these types of experiments (Supplementary Figure S7C).

The senescent phenotype under these conditions is similar to replicative SEN as demonstrated by downregulation of a handful of randomly selected genes (Fig. 7c, d and e). In SR hADSCs transfected with a full set of SA-miRNA mimics, we observed downregulation of endogenous mRNA from the enriched functional network that represents *SA-miRNA target genes* such as *SUZ12, NAP1L1, SMARCD2*, *SAP18, IGF2BP3, CHD2* and *CHD4* (Figs. 6 and 7c), as well as a number of the genes *not targeted by SA-miRNAs* but, nevertheless, shown to be downregulated upon replicative SEN*,* such as *SMARCA1, CHD8, HDAC3, HDAC5* and *HDAC9* (Fig. 7d and Supplementary Tables S3 and S4). Our functional test suggests that even transient delivery of a full set of mature SA-miRNA mimics in SR hADSCs is necessary and sufficient to trigger a cascade of primary (through downregulation of SA-miRNA targets) and secondary (downregulation of downstream genes) events resulting in the senescent phenotype of hADSCs.

These results further support the observation that SA-miRNAs from the *MIR17HG* and *MIR100HG* clusters with reported opposing biological activities (Fig. 2c and d) are functionally linked to the process of establishment of the cellular SEN phenotype in hADSCs and identify the direct targets through which these SA-miRNAs could exert their regulatory roles.

## Discussion

Cellular senescence involves a coordination of an extremely broad range of processes from telomere homeostasis, genome instability and DNA damage, to drastic changes in chromatin organization, inflammatory signaling and metabolic, cytoskeletal and paracrine changes.^67^ Due to the profound secretory phenotype of senescent cells, their impact on organs and tissues is far from neutral. The role of senescence in the aging of adult stem cells is tightly linked to tissue maintenance and homeostasis and often viewed as an irreversible barrier to immortalization and tumorigenesis under the assumption that senescence evolved to suppress tumorigenesis.^68, 69^ This view has been intensely debated in recent years.^70, 71^ Contrary to the hypothesis that senescence and tumorigenicity are always permanently connected and mutually exclusive, recent data monitoring *p16INK4a* in mice indicate that the activation of this hallmark of senescence is, in fact, a characteristic of all emerging cancers,^72^ thus suggesting that cellular senescence might be a quasi-stable and/or plastic cellular state prone to cancerogenesis rather than a cancer preventive mechanism.

It has been proposed that in addition to protein-coding oncogenes and tumor suppressor genes, one has to take into account miRNAs and their regulatory networks in order to understand the complex processes underlying malignant transformation.^73–75^ Concurrently, there is a considerable interest in the development of senescence-targeted cancer and metabolic therapeutics. This goal suggests the need for a system-level view of the regulation of senescence in order to identify not only reliable markers of senescence that will translate into human settings, but also to understand the intimate connections between oncogenic events and senescence better. Such approach will allow for defining a set of genetic targets that can elicit, modulate and, most importantly, balance mechanisms involved in tumor initiation, growth and progression. Our study contributes exactly to such initiative and provides measurable and reliable markers (composing SA-miRNAs and their target genes) that can be further deployed to identify mechanistic underpinnings of oncogenic and tumor-suppressive balance. Such resource has not been previously available and technically challenging to obtain.

In this study, we not only have identified a set of SA-miRNAs originating from oncogenic *MIR17HG* and tumor-suppressive *MIR100HG* clusters as potent controllers of complex and coordinated interactions among several cellular sub-processes associated with cellular senescence. Importantly, we have demonstrated functional significance of these SA-miRNAs in establishing senescent phenotype in adult adipose-derived stem cells (Fig. 7a and b, and Supplementary Figure S7E, F and G). In addition, our study functionally defines a set of gene-targets regulated by these SA-miRNAs. These target genes are linked to interconnected biological networks that control cell fate switches and, most importantly, our data suggest that balancing of these network components might be the cause and consequence of SA-miRNA–target interactions. In our proposed model of the action of SA-miRNAs, it remains unclear if forced overexpression of SA-miRNAs in SR hADSCs has physiologically relevant consequences and fully recapitulates the complexity of the hADSCs senescent phenotype in the long run. However, even in transient transfection experiments, the introduction of a set of mimic miRNAs representing SA-miRNAs produces similar to replicative SEN transcriptional outcomes, such as miRNA-induced decay of the direct SA-miRNA targets (Figs. 6 and 7c, e, and Supplementary Tables S1 and S2) and an adequate downstream response recorded as downregulation of the indirect SA-miRNA targets (Fig. 7d and e and Supplementary Table S3). Therefore, we believe that our approach accurately and comprehensively identifies SA-miRNA targets as well as functionally supports the critical role of SA-miRNAs in establishing early stages of cellular senescence. This regulatory network of miRNAs is probably highly dynamic and sensitive to external signaling. Recently, other methods were developed to capture miRNA-target interactions, however, there is still no consensus as to which approach is the most accurate and appropriate for readout of biological function.^31^

While our study does not uncover the underlying molecular mechanisms of how SA-miRNA–target interactions balance the network, particularly when miRNA expression upon SEN originates from the clusters with seemingly opposing biological roles in oncogenesis, one can put forth the following hypothesis, that similar to miRNAs and transcriptional factors that often form feed forward loops,^31, 76–78^ the SA-miRNAs in hADSCs have evolved to provide a precise target gene expression among critical SEN-associated sub-processes, such as networks controlling cell cycle, transcription/translation and, ultimately, chromatin assembly.^70, 79^ In our view, the direct effects of oncogenic SA-miRNAs on target genes might be counter-balanced by the action of tumor-suppressive SA-miRNAs on a set of different targets within the same or an interconnected network, thus buffering stochastic fluctuations^73^ in order to increase the precision of the expression of the entire regulatory network.

Relevant to this discussion is an example of malignant soft tissue tumors-rare, but aggressive malignancies. Liposarcoma is the most common soft tissue sarcoma in adults characterized by a high rate of local recurrence and high metastatic potential. It has been a subject of intensive debate that the local tumor microenvironment, cell-cell, and cell-stromal interactions are inherent in serving biochemical functions. Malignant cells perpetually stimulate host stromal, vascular and tissue-specific adult stem cells to conduct physiological invasion.^73, 74^ Senescent cells through their specific transcriptional programs and senescence-associated secretory phenotype might create a permissive “field” for the malignant cell to grow (reviewed in).^80^ On the other hand, various secreted by the growing tumor signaling molecules, such as cytokines, chemo-tactics and growth factors, also are can modulate the local environment and, in their turn, can feedback on the senescent cells (paracrine loop). Therefore, it is theoretically possible that a shift in the delicate balance between the oncogenic and tumor-suppressive events imposed by SA-miRNAs, as well as transcriptional cascades targeted by these SA-miRNAs could come to play when adipose tissue homeostasis is perturbed in disease. To support such assumption, recent study elegantly demonstrated that, indeed, sarcoma itself, prior to sarcoma invasion, can impose miRNA expression changes in pre-adipocytes and other mesechymal stromal cells through the paracrine effectors.^81^ It is plausible that similar events could be imposed on senescent ADSCs. Therefore, we speculate that the sequence of oncogenic events that can push senescent cells into a cancerous transformation may depend on the prevalence of autocrine or paracrine signals, which when received by senescent cells can shift the balance in levels of mature miRNAs. Such shift in the balancing act of microRNAs might enable underlying changing in epigenetic regulation, similar to the proposed role of the EMT and its reversal mesenchymal-epithelial transition in controlling or avoiding cancer.^70^ These autocrine or paracrine signaling upon senescence of hADSCs are subject to further investigations.

We are aware of the argument that miRNA’s impact on individual targets might depend on many dynamic factors, such as cellular localization of miRNAs and their targets, their relative concentrations, and the context-specific effects of other regulators, including transcriptional factors and RNA-binding proteins.^82–85^ Despite these arguments, the utility of our study is in the computational and functional identification of comprehensive and interconnected SA-miRNA-target regulatory networks describing the phenotypic manifestation of hADSC- SEN. Although cumulative studies of miRNA expression in different models of cellular senescence suggest that miRNA signatures of senescence are likely to be cell-type and trigger dependent with a common core of effectors, we believe that an adequate signature of senescence for adult adipose-derived stem cells would consist of a number of well-established markers, among which, SA-miRNAs and their direct targets may well be a useful addition for assessment of adipose tissue metabolism and regeneration. The SA-miRNAs identified in this study are particularly promising considering that the endogenous circulating miRNAs in human serum^86^ and other body fluids, such as cerebrospinal fluid, colostrum, peritoneal fluid, saliva, seminal fluid and urine^87^ are highly stable. Our study provides a matrix of potentially measurable markers of replicative SEN in hADSCs with a viable hypothesis that the coordinated action of these SA-miRNAs provide for the balance between driving and restraining tumor progression and may be further exploited to define correlations between these senescence-associated miRNAs and other relevant markers of disease progression as well as responses to therapeutic treatments.

## Materials and methods

### Isolation and culture of hMSCs

Human adipose derived stem cells were isolated from human subcutaneous white adipose tissue collected during liposuction procedures. The lipoaspirate was suspended in Hank’s buffered salt solution (Life Technology), 3.5% Bovine Serum Albumin (BSA, Sigma), 1% Collagenase Type II (Sigma) in 1:3 w/v ratio and shaken at 37 °C for 50 min. The cells were filtered through a 70 μm mesh cell strainer (BD Falcon #352350), treated with red blood cell lysis buffer (150 mM NH4Cl, 10 mM KHCO3, 0.1 mM EDTA, pH 7.3), and expanded ex-vivo in DMEM/F12 complete medium (DMEM/F12, 10% FBS, 100 U/ml penicillin, 100 **μ**g/ml streptomycin; Life technology) in 10% CO2 at 37 °C and passaged at 80% confluency, changing medium every 72–96 h. Cumulative population doublings were calculated by summing the population doublings (PD = log(N/N_0_) × 3.33, where N_0_ is the number of cells plated in the flask, and N is the number of cells harvested at this passage) across multiple passages as a function of the number of days it was grown in culture.

### Surface marker characterization

Five × 10^5^ cells either SR (PD8) or SEN (PD40) each were incubated for 30 min on ice in the dark with fluorochrome-conjugated antibodies (CD31, CD44, CD45 and CD105; Invitrogen) in PBS with 1% BSA (Sigma), washed and analyzed in a Guava EasyCyte Mini System (Guava Technologies, Millipore). Data analysis was done with FlowJo software (Tree Star, Ashland, OR).

### SA-β-Gal staining

A SA-β-Gal activity assay was done according to manufacturer’s instructions (BioVision). The cells were washed with PBS and fixed with fixation solution for 15 min at room temperature. The cells were washed with PBS twice and X-Gal staining solution was added with a staining supplement per well and incubated overnight at 37 °C. The cells were washed twice with PBS, and the images were captured using a microscope (Nikon, TE300, DXM1200 Digital Camera, Japan).

### Proteomic analysis and transcriptome analysis with RNA-seq

The details are given in Supplementary Experimental Procedures.

### miRNA target identification

The mRNA targets of differentially expressed miRNAs were identified using the program mirSVR. This program was chosen because it combines miRNA-mRNA binding site sequence analysis with several additional sources of contextual information, including gene expression data from miRNA transfection experiments, in order to make target predictions (Supplementary Figure S6). Accordingly, mirSVR has been shown to yield a relatively low rate of false positive predictions for miRNA target identification.^88^ mirSVR also provides scores to rank the predicted targets, and, for this study, targets with a score <−0.2 were selected for further analysis.

### Network-based functional enrichment analysis

The details of network analysis are given in Supplementary Experimental Procedures.

### RT-PCR

Total cellular RNA was extracted from cells using the TRIzol reagent^™^ (Life Technologies) according to manufacturer’s instructions. The miRNA was isolated using a mirPremier miRNA isolation kit (Sigma-Aldrich), RNA and miRNA were quantified with a NanoDrop ND-2000 Spectrophotometer (Thermo Scientific). The cDNA was synthesized by adding the purified RNA and oligo(dT) primers by using Superscript III reverse transcriptase (Life Technologies). Primers were designed by Primer3 software and shown in Supplementary Table S1. For miRNA cDNA synthesis, the Mystic miRNA cDNA synthesis Mix kit (Sigma-Aldrich) was used. All miRNA assay primers were bought from Sigma-Aldrich.

### Real-time quantitative PCR

Quantification of mRNA and miRNA expression for candidate genes was performed by real-time quantitative PCR (qRT-PCR) using the LightCycler^®^ 480 Real-Time PCR System (Roche). Total RNA and miRNA was reverse transcribed by using the high capacity superscript III reverse transcriptase (Life Technologies) and the Mystic miRNA cDNA synthesis Mix kit (Sigma-Aldrich), respectively. Primers were designed by primer3 software (Supplementary Table S5). All miRNA assay primers were bought from Sigma-Aldrich. qRT-PCR reactions were performed with the power SYBR^®^ green PCR master mix and the mystic miRNA SYBR green qPCR ReadyMix in a MicroAmp optical 96-well reaction plate. The PCR amplification of total RNA was performed in a LightCycler^®^ 480 Real-Time PCR System (Roche) using the following program: Cycle 1, 95 °C for 10 min. Cycle 2, 40 cycles of 95 °C for 15 sec, 60 °C for 60 sec. Cycle threshold (CT) values were automatically obtained. Relative expression values of RNA were obtained by normalizing CT values of the mRNA genes in comparison with CT values of the endogenous control (beta-actin) using the CT method. The PCR amplification of miRNA was performed in a LightCycler^®^ 480 Real-Time PCR System (Roche) using the following program: Cycle 1, 95 °C for 2 min. Cycle 2, 40 cycles of 95 °C for 5 sec, 60 °C for 30 sec. Relative expression values of miRNA were obtained by normalizing CT values of the miRNA genes in comparison with CT values of the endogenous control (U6) using the CT method.

**Table 1:** mRNA targets down-regulated in SEN

mir-17-5p (MIMAT0000070)							
Gene Symbol	RefSeq ID	mirSVR Score	SR	SEN	dRPKM	FC	D
TFPI2	NM_006528	−0.25	528.90	225.72	−303.17	−1.23	8.34
IL8	NM_000584	−1.18	89.60	8.90	−80.69	−3.33	7.16
INHBA	NM_002192	−0.36	282.13	177.25	−104.88	−0.67	6.75
UPF3A	NM_023011	−0.31	18.45	1.27	−17.18	−3.86	5.63
CD36	NM_001127443	−0.28	47.21	11.74	−35.47	−2.01	5.53
MMP1	NM_002421	−0.33	69.30	31.02	−38.28	−1.16	5.38
RPA2	NM_002946	−0.64	16.56	1.76	−14.79	−3.23	5.05
GMPS	NM_003875	−0.29	13.70	1.24	−12.47	−3.47	5.03
STC1	NM_003155	−1.12	39.40	13.31	−26.09	−1.57	4.96
ACTL6A	NM_004301	−0.29	23.69	4.90	−18.79	−2.27	4.80
DCTN3	NM_024348	−0.48	19.61	3.29	−16.33	−2.58	4.78
LANCL1	NM_001136574	−0.63	8.61	0.67	−7.93	−3.68	4.74
PARP3	NM_001003931	−0.44	12.09	1.29	−10.80	−3.23	4.71
F3	NM_001993	−1.18	11.90	1.27	−10.63	−3.23	4.70
DCBLD2	NM_080927	−0.58	63.32	39.36	−23.96	−0.69	4.63
TPRG1L	NM_182752	−1.03	35.20	16.26	−18.95	−1.11	4.39
FBXO28	NM_001136115	−0.50	6.41	0.58	−5.83	−3.47	4.30
URGCP	NM_001077663	−0.31	7.87	0.84	−7.03	−3.23	4.28
SLC25A44	NM_014655	−0.23	7.84	0.84	−7.01	−3.23	4.28
FBXO28	NM_015176	−0.58	6.18	0.56	−5.62	−3.47	4.27
MAF1	NM_032272	−0.35	18.96	5.13	−13.83	−1.89	4.23
SQSTM1	NM_003900	−0.98	27.66	11.51	−16.15	−1.27	4.21
M6PR	NM_002355	−0.85	9.08	1.18	−7.90	−2.94	4.19
SAR1B	NM_016103	−1.28	14.67	3.25	−11.41	−2.17	4.13
ZBTB4	NM_020899	−1.05	12.64	2.55	−10.08	−2.31	4.05
UFD1L	NM_001035247	−0.98	10.48	1.75	−8.72	−2.58	4.05
SLC35A5	NM_017945	−0.76	7.93	1.03	−6.89	−2.94	4.05
RAD23B	NM_002874	−0.76	30.48	15.32	−15.17	−0.99	4.05
MITD1	NM_138798	−0.20	14.23	3.34	−10.89	−2.09	4.03
UFD1L	NM_005659	−0.99	10.16	1.70	−8.46	−2.58	4.02
PTP4A1	NM_003463	−0.46	20.89	7.77	−13.12	−1.43	3.98
RRM2	NM_001034	−0.73	7.13	0.93	−6.21	−2.94	3.95
SERPINB8	NM_002640	−0.55	6.91	0.90	−6.01	−2.94	3.92
SLC16A7	NM_004731	−0.51	6.58	0.86	−5.72	−2.94	3.87
GPR137B	NM_003272	−0.76	8.88	1.49	−7.39	−2.58	3.87
MKRN1	NM_013446	−1.08	12.33	2.89	−9.44	−2.09	3.86
NRBP1	NM_013392	−0.76	34.15	20.71	−13.44	−0.72	3.82
PFKP	NM_002627	−1.29	30.21	17.14	−13.07	−0.82	3.80
CYTSB	NM_001033553	−0.65	5.90	0.77	−5.13	−2.94	3.77
LSM5	NM_012322	−0.42	8.06	1.35	−6.71	−2.58	3.77
FAM103A1	NM_031452	−0.89	11.30	2.65	−8.65	−2.09	3.75
TPP1	NM_000391	−0.68	10.99	2.58	−8.42	−2.09	3.72
GTDC1	NM_001006636	−1.08	6.73	1.13	−5.60	−2.58	3.58
SLC39A6	NM_012319	−0.94	23.60	12.58	−11.02	−0.91	3.58
ELK3	NM_005230	−1.22	13.24	4.23	−9.01	−1.64	3.57
MYO10	NM_012334	−0.28	16.54	6.64	−9.90	−1.32	3.56
VPS45	NM_007259	−0.40	6.43	1.08	−5.36	−2.58	3.54
PURA	NM_005859	−1.02	14.18	5.16	−9.02	−1.46	3.49
MRPL24	NM_145729	−1.24	19.55	9.82	−9.73	−0.99	3.43
MGAT2	NM_002408	−0.21	14.33	5.60	−8.73	−1.36	3.41
ACIN1	NM_014977	−0.21	14.16	5.53	−8.63	−1.36	3.39
TXNIP	NM_006472	−1.27	27.42	17.63	−9.79	−0.64	3.35
COMMD10	NM_016144	−0.30	20.00	10.66	−9.34	−0.91	3.35
C1orf9	NM_014283	−1.12	9.61	2.68	−6.93	−1.84	3.35
CCNDBP1	NM_012142	−0.34	9.22	2.49	−6.73	−1.89	3.33
UBE2B	NM_003337	−0.93	10.83	3.46	−7.36	−1.64	3.32
NTN4	NM_021229	−1.22	23.64	14.28	−9.36	−0.73	3.31
RSU1	NM_012425	−0.21	15.87	7.28	−8.59	−1.12	3.30
CENPQ	NM_018132	−1.27	7.49	1.76	−5.74	−2.09	3.28
SLC35F5	NM_025181	−1.02	22.73	13.86	−8.87	−0.71	3.23
ABR	NM_021962	−0.34	9.37	2.89	−6.48	−1.70	3.18
RAB21	NM_014999	−0.43	18.73	10.40	−8.33	−0.85	3.17
TBP	NM_003194	−0.24	6.80	1.59	−5.20	−2.09	3.17
GNPDA2	NM_138335	−1.25	6.75	1.58	−5.17	−2.09	3.16
CDCA4	NM_145701	−0.22	6.72	1.58	−5.14	−2.09	3.16
ABTB1	NM_172027	−0.27	11.79	4.61	−7.18	−1.36	3.15
AKTIP	NM_022476	−0.47	17.71	9.69	−8.02	−0.87	3.13
YIPF2	NM_024029	−0.25	22.38	14.13	−8.25	−0.66	3.12
MAGT1	NM_032121	−0.29	16.10	8.30	−7.79	−0.95	3.11
GOLGB1	NM_004487	−0.34	12.27	5.16	−7.11	−1.25	3.09
SFRS4	NM_005626	−0.24	13.30	6.00	−7.30	−1.15	3.09
TIPARP	NM_015508	−0.40	12.20	5.27	−6.93	−1.21	3.04
CEP120	NM_153223	−1.19	7.14	1.93	−5.21	−1.89	3.04
CHD9	NM_025134	−1.00	7.91	2.38	−5.52	−1.73	3.01
KDSR	NM_002035	−0.29	10.46	4.09	−6.37	−1.36	3.00
ATMIN	NM_015251	−0.60	9.05	3.12	−5.93	−1.54	2.99
ZFP91	NM_053023	−0.61	11.31	4.77	−6.53	−1.24	2.98
LIN7B	NM_022165	−1.10	10.29	4.02	−6.27	−1.36	2.97
UBE3C	NM_014671	−0.54	14.49	7.62	−6.88	−0.93	2.93
ACBD5	NM_145698	−0.71	9.75	3.81	−5.94	−1.36	2.91
NAP1L1	NM_004537	−0.25	20.71	13.62	−7.09	−0.60	2.89
NBL1	NM_182744	−0.35	13.82	7.36	−6.45	−0.91	2.84
FGL2	NM_006682	−0.77	18.81	12.09	−6.71	−0.64	2.82
RAB11FIP5	NM_015470	−1.13	12.52	6.29	−6.23	−0.99	2.82
VPS26A	NM_001035260	−0.69	9.11	3.56	−5.55	−1.36	2.82
RBL2	NM_005611	−1.08	10.07	4.35	−5.72	−1.21	2.79
PDZD11	NM_016484	−1.15	18.70	12.18	−6.52	−0.62	2.77
SSFA2	NM_006751	−0.80	12.51	6.45	−6.06	−0.95	2.77
NFAT5	NM_138713	−0.21	9.93	4.34	−5.59	−1.20	2.76
VPS26A	NM_004896	−0.71	8.63	3.37	−5.26	−1.36	2.75
SSFA2	NM_001130445	−0.77	12.23	6.31	−5.92	−0.95	2.74
CRK	NM_005206	−0.90	12.79	6.82	−5.97	−0.91	2.73
DPM2	NM_003863	−0.26	11.72	5.89	−5.83	−0.99	2.73
PPP2R5E	NM_006246	−1.19	10.00	4.51	−5.49	−1.15	2.71
ARL1	NM_001177	−0.90	18.55	12.29	−6.26	−0.59	2.71
ATP2B1	NM_001682	−0.50	10.31	4.93	−5.39	−1.07	2.65
CRK	NM_016823	−0.93	11.88	6.33	−5.55	−0.91	2.63
ATP2B1	NM_001001323	−0.44	10.08	4.82	−5.27	−1.07	2.62
MGLL	NM_001003794	−0.24	16.69	10.87	−5.82	−0.62	2.61
DYNC1LI2	NM_006141	−1.14	16.12	10.50	−5.62	−0.62	2.57
RHOT1	NM_001033568	−0.34	11.74	6.42	−5.32	−0.87	2.56
RTCD1	NM_001130841	−0.43	10.85	5.78	−5.07	−0.91	2.51
DNM1L	NM_012062	−0.53	15.16	9.87	−5.28	−0.62	2.48

mir-18a-5p (MIMAT0000072)							
Gene Symbol	RefSeq ID	mirSVR Score	SR	SEN	dRPKM	FC	D
TSPYL2	NM_022117	−0.30	665.60	381.06	−284.54	−0.80	8.19
HIST1H4H	NM_003543	−0.81	62.31	8.12	−54.19	−2.94	6.47
BCAR3	NM_003567	−1.25	15.52	0.96	−14.56	−4.02	5.57
MSRB2	NM_012228	−0.38	15.73	1.68	−14.05	−3.23	5.00
NDUFC2	NM_004549	−0.75	26.15	6.13	−20.02	−2.09	4.80
F3	NM_001993	−0.34	11.90	1.27	−10.63	−3.23	4.70
ELOVL1	NM_022821	−0.72	36.66	14.33	−22.34	−1.36	4.68
PCYT1A	NM_005017	−0.79	21.26	5.75	−15.51	−1.89	4.38
EGLN2	NM_053046	−0.35	10.38	1.35	−9.03	−2.94	4.33
FBXO28	NM_001136115	−0.57	6.41	0.58	−5.83	−3.47	4.30
FBXO28	NM_015176	−0.65	6.18	0.56	−5.62	−3.47	4.27
MEF2BNB	NM_001145784	−0.30	12.65	2.12	−10.53	−2.58	4.26
UQCRQ	NM_014402	−0.79	24.50	9.58	−14.93	−1.36	4.13
ZBTB4	NM_020899	−1.16	12.64	2.55	−10.08	−2.31	4.05
B4GALT7	NM_007255	−0.48	10.49	1.76	−8.74	−2.58	4.05
FZD8	NM_031866	−0.47	7.31	0.95	−6.36	−2.94	3.97
PDGFC	NM_016205	−1.17	12.91	3.03	−9.88	−2.09	3.91
EPDR1	NM_017549	−0.26	30.89	17.53	−13.37	−0.82	3.83
TPP1	NM_000391	−0.41	10.99	2.58	−8.42	−2.09	3.72
COIL	NM_004645	−0.23	6.88	1.15	−5.73	−2.58	3.60
CA12	NM_001218	−0.59	18.89	8.40	−10.49	−1.17	3.59
NT5C3L	NM_052935	−0.24	16.39	6.40	−9.98	−1.36	3.59
NAP1L1	NM_139207	−0.21	23.85	12.96	−10.89	−0.88	3.56
PAPSS2	NM_001015880	−0.99	20.80	10.23	−10.58	−1.02	3.55
AP3S1	NM_001284	−1.26	22.10	11.77	−10.32	−0.91	3.49
ACIN1	NM_014977	−0.24	14.16	5.53	−8.63	−1.36	3.39
SNX5	NM_014426	−0.27	16.36	7.38	−8.98	−1.15	3.37
C1orf9	NM_014283	−1.04	9.61	2.68	−6.93	−1.84	3.35
NAE1	NM_003905	−1.34	12.85	5.02	−7.83	−1.36	3.26
TSC22D3	NM_198057	−1.10	14.84	6.69	−8.15	−1.15	3.24
C9orf114	NM_016390	−0.71	6.98	1.64	−5.34	−2.09	3.20
GNPDA2	NM_138335	−0.87	6.75	1.58	−5.17	−2.09	3.16
DDX42	NM_203499	−1.09	16.50	8.51	−7.99	−0.95	3.15
GCLC	NM_001498	−0.99	12.91	5.57	−7.33	−1.21	3.12
VPS4B	NM_004869	−0.33	11.50	4.49	−7.00	−1.36	3.12
SORBS3	NM_005775	−0.31	9.12	2.92	−6.20	−1.64	3.10
DUSP5	NM_004419	−0.28	13.31	6.00	−7.31	−1.15	3.09
NDFIP1	NM_030571	−0.90	15.12	7.60	−7.52	−0.99	3.08
NT5C2	NM_001134373	−0.31	8.29	2.65	−5.64	−1.64	2.99
HSBP1L1	NM_001136180	−0.30	10.34	4.04	−6.30	−1.36	2.98
ITGA2	NM_002203	−0.63	11.52	5.01	−6.50	−1.20	2.96
MKI67IP	NM_032390	−0.27	19.37	12.23	−7.14	−0.66	2.91
PRKAR2A	NM_004157	−0.47	9.79	3.82	−5.96	−1.36	2.91
RBL2	NM_005611	−0.61	10.07	4.35	−5.72	−1.21	2.79
SEL1L3	NM_015187	−0.80	17.64	11.34	−6.30	−0.64	2.73
PPP2R5E	NM_006246	−0.32	10.00	4.51	−5.49	−1.15	2.71
ENDOD1	NM_015036	−0.24	8.28	3.23	−5.04	−1.36	2.70
XPO6	NM_015171	−0.35	14.64	8.92	−5.71	−0.71	2.61

mir-19a-3p (MIMAT0000073)							
Gene Symbol	RefSeq ID	mirSVR Score	SR	SEN	dRPKM	FC	D
IL8	NM_000584	−0.46	89.60	8.90	−80.69	−3.33	7.16
IL33	NM_033439	−0.59	35.40	1.12	−34.28	−4.98	7.13
NGFRAP1	NM_206915	−0.66	34.92	3.15	−31.77	−3.47	6.08
NGFRAP1	NM_014380	−0.65	31.96	3.41	−28.56	−3.23	5.82
UPF3A	NM_023011	−0.29	18.45	1.27	−17.18	−3.86	5.63
BCAR3	NM_003567	−0.22	15.52	0.96	−14.56	−4.02	5.57
NDUFB2	NM_004546	−0.43	68.00	30.66	−37.34	−1.15	5.35
RPS27L	NM_015920	−0.62	78.82	47.61	−31.22	−0.73	5.02
FAM129B	NM_022833	−1.14	87.20	56.11	−31.09	−0.64	5.00
PRKRA	NM_003690	−0.95	15.60	1.66	−13.93	−3.23	4.99
STAMBPL1	NM_020799	−0.54	14.26	1.52	−12.74	−3.23	4.89
EMC7	NM_020154	−0.53	46.72	20.18	−26.54	−1.21	4.88
ACTL6A	NM_004301	−0.52	23.69	4.90	−18.79	−2.27	4.80
RAP1B	NM_001010942	−1.15	55.53	30.38	−25.15	−0.87	4.73
PARP3	NM_001003931	−0.78	12.09	1.29	−10.80	−3.23	4.71
F3	NM_001993	−1.04	11.90	1.27	−10.63	−3.23	4.70
MEDAG	NM_032849	−1.18	53.08	29.21	−23.87	−0.86	4.66
DCBLD2	NM_080927	−0.32	63.32	39.36	−23.96	−0.69	4.63
SLC31A2	NM_001860	−1.22	13.14	1.71	−11.42	−2.94	4.58
CCNC	NM_005190	−0.41	19.08	3.95	−15.13	−2.27	4.53
WDR44	NM_019045	−1.34	8.12	0.73	−7.39	−3.47	4.51
RALA	NM_005402	−0.42	35.93	16.20	−19.73	−1.15	4.45
TPRG1L	NM_182752	−0.55	35.20	16.26	−18.95	−1.11	4.39
POLE4	NM_019896	−0.75	18.60	4.36	−14.24	−2.09	4.37
GNAQ	NM_002072	−0.27	17.69	4.15	−13.54	−2.09	4.30
SLC25A44	NM_014655	−0.39	7.84	0.84	−7.01	−3.23	4.28
FBXO28	NM_015176	−0.23	6.18	0.56	−5.62	−3.47	4.27
PPTC7	NM_139283	−0.70	7.60	0.81	−6.79	−3.23	4.25
TSN	NM_004622	−0.68	17.48	4.45	−13.02	−1.97	4.20
FAM69A	NM_001006605	−0.96	8.97	1.17	−7.80	−2.94	4.17
COX6C	NM_004374	−0.84	25.30	9.89	−15.41	−1.36	4.17
ZBTB4	NM_020899	−0.90	12.64	2.55	−10.08	−2.31	4.05
UFD1L	NM_001035247	−0.69	10.48	1.75	−8.72	−2.58	4.05
RAD23B	NM_002874	−0.90	30.48	15.32	−15.17	−0.99	4.05
UFD1L	NM_005659	−0.71	10.16	1.70	−8.46	−2.58	4.02
PTP4A1	NM_003463	−0.21	20.89	7.77	−13.12	−1.43	3.98
C2orf76	NM_001017927	−0.26	13.63	3.20	−10.43	−2.09	3.98
FZD8	NM_031866	−0.64	7.31	0.95	−6.36	−2.94	3.97
KLHL12	NM_021633	−0.74	6.98	0.91	−6.07	−2.94	3.93
POU4F1	NM_006237	−0.78	11.54	2.39	−9.15	−2.27	3.92
BMPER	NM_133468	−0.73	6.86	0.89	−5.96	−2.94	3.91
GPR137B	NM_003272	−0.95	8.88	1.49	−7.39	−2.58	3.87
PIK3IP1	NM_001135911	−0.63	14.06	3.80	−10.26	−1.89	3.85
RBM26	NM_022118	−0.27	6.45	0.84	−5.61	−2.94	3.85
RPL27A	NM_000990	−0.20	21.76	9.33	−12.43	−1.22	3.83
NRBP1	NM_013392	−0.96	34.15	20.71	−13.44	−0.72	3.82
PIK3IP1	NM_052880	−0.67	13.61	3.68	−9.93	−1.89	3.81
REP15	NM_001029874	−0.30	11.37	2.67	−8.70	−2.09	3.76
BMP6	NM_001718	−1.04	22.51	10.75	−11.76	−1.07	3.71
TMEM167B	NM_020141	−0.55	12.24	3.31	−8.93	−1.89	3.68
SUPV3L1	NM_003171	−0.43	7.33	1.23	−6.10	−2.58	3.67
RAB3B	NM_002867	−0.90	26.41	14.89	−11.52	−0.83	3.62
GNRH1	NM_001083111	−0.28	10.11	2.37	−7.74	−2.09	3.62
VDAC3	NM_005662	−0.20	30.78	19.10	−11.68	−0.69	3.61
GTDC1	NM_001006636	−0.23	6.73	1.13	−5.60	−2.58	3.58
ELK3	NM_005230	−1.08	13.24	4.23	−9.01	−1.64	3.57
NCBP2	NM_007362	−0.61	13.12	4.20	−8.93	−1.64	3.56
NAP1L1	NM_139207	−0.49	23.85	12.96	−10.89	−0.88	3.56
CCNA2	NM_001237	−0.65	6.48	1.08	−5.39	−2.58	3.54
SDC1	NM_002997	−1.15	18.60	8.53	−10.07	−1.12	3.52
PHLDA1	NM_007350	−0.43	18.83	8.73	−10.10	−1.11	3.52
PURA	NM_005859	−1.19	14.18	5.16	−9.02	−1.46	3.49
RAB13	NM_002870	−1.02	29.19	18.43	−10.76	−0.66	3.49
SAP18	NM_005870	−0.51	12.38	3.96	−8.42	−1.64	3.49
PSMD9	NM_002813	−0.31	12.16	3.89	−8.27	−1.64	3.46
MGAT2	NM_002408	−0.39	14.33	5.60	−8.73	−1.36	3.41
BOLA3	NM_212552	−0.37	14.02	5.48	−8.54	−1.36	3.38
TMEM106C	NM_001143842	−0.41	8.15	1.91	−6.24	−2.09	3.37
SNX5	NM_014426	−0.89	16.36	7.38	−8.98	−1.15	3.37
C1orf9	NM_014283	−1.18	9.61	2.68	−6.93	−1.84	3.35
ANTXR2	NM_058172	−0.40	17.71	8.68	−9.03	−1.03	3.34
UBE2D2	NM_003339	−1.01	10.54	3.37	−7.17	−1.64	3.28
SEC14L1	NM_003003	−0.66	7.07	1.66	−5.41	−2.09	3.21
ABR	NM_021962	−0.97	9.37	2.89	−6.48	−1.70	3.18
ATXN10	NM_013236	−1.11	16.33	8.20	−8.12	−0.99	3.18
RAB21	NM_014999	−0.45	18.73	10.40	−8.33	−0.85	3.17
TROVE2	NM_004600	−0.88	11.98	4.68	−7.30	−1.36	3.17
UBE2V1	NM_001032288	−0.43	18.21	9.96	−8.25	−0.87	3.17
NDFIP2	NM_019080	−1.04	11.68	4.56	−7.12	−1.36	3.14
YTHDF2	NM_016258	−1.11	9.31	2.98	−6.33	−1.64	3.13
AKTIP	NM_022476	−0.36	17.71	9.69	−8.02	−0.87	3.13
GCLC	NM_001498	−0.23	12.91	5.57	−7.33	−1.21	3.12
VPS4B	NM_004869	−1.18	11.50	4.49	−7.00	−1.36	3.12
GLRX5	NM_016417	−1.06	15.48	7.78	−7.70	−0.99	3.11
SETD7	NM_030648	−0.31	12.92	5.63	−7.30	−1.20	3.11
NDFIP1	NM_030571	−1.10	15.12	7.60	−7.52	−0.99	3.08
SPRY2	NM_005842	−0.27	11.04	4.32	−6.73	−1.36	3.07
UBA3	NM_003968	−1.26	20.77	12.89	−7.88	−0.69	3.06
TIPARP	NM_015508	−1.06	12.20	5.27	−6.93	−1.21	3.04
INSIG1	NM_198337	−1.01	12.38	5.58	−6.80	−1.15	2.99
ATMIN	NM_015251	−1.02	9.05	3.12	−5.93	−1.54	2.99
HNRNPF	NM_001098206	−1.10	22.48	14.90	−7.58	−0.59	2.98
ZFP91	NM_053023	−0.65	11.31	4.77	−6.53	−1.24	2.98
ARL6IP1	NM_015161	−0.76	10.29	4.02	−6.27	−1.36	2.98
ITGA2	NM_002203	−0.84	11.52	5.01	−6.50	−1.20	2.96
ACBD5	NM_145698	−1.14	9.75	3.81	−5.94	−1.36	2.91
NAP1L1	NM_004537	−0.36	20.71	13.62	−7.09	−0.60	2.89
INSIG1	NM_005542	−1.02	11.18	5.04	−6.14	−1.15	2.86
FMR1	NM_002024	−1.10	12.33	6.19	−6.13	−0.99	2.80
UTRN	NM_007124	−0.22	11.04	5.13	−5.91	−1.11	2.79
PIP4K2B	NM_003559	−0.30	7.68	2.65	−5.03	−1.54	2.79
SECISBP2L	NM_014701	−0.70	11.40	5.61	−5.80	−1.02	2.73
TMEM189-UBE2V1	NM_199203	−0.43	13.31	7.28	−6.03	−0.87	2.73
SEL1L3	NM_015187	−0.81	17.64	11.34	−6.30	−0.64	2.73
PPP2R5E	NM_006246	−1.23	10.00	4.51	−5.49	−1.15	2.71
IER5L	NM_203434	−0.24	16.24	10.08	−6.16	−0.69	2.71
CS	NM_004077	−0.76	13.05	7.14	−5.91	−0.87	2.71
DYNC1LI2	NM_006141	−0.93	16.12	10.50	−5.62	−0.62	2.57
NECAP1	NM_015509	−0.31	10.98	5.85	−5.13	−0.91	2.53
RTCD1	NM_001130841	−0.29	10.85	5.78	−5.07	−0.91	2.51
RUFY1	NM_025158	−0.52	10.85	5.78	−5.07	−0.91	2.51
DNM1L	NM_012062	−0.27	15.16	9.87	−5.28	−0.62	2.48
TNIP1	NM_006058	−0.48	13.47	8.36	−5.11	−0.69	2.45

mir-20a-5p (MIMAT0000075)							
Gene Symbol	RefSeq ID	mirSVR Score	SR	SEN	dRPKM	FC	D
TFPI2	NM_006528	−1.23	528.90	225.72	−303.17	−1.23	8.34
IL8	NM_000584	−1.18	89.60	8.90	−80.69	−3.33	7.16
INHBA	NM_002192	−0.36	282.13	177.25	−104.88	−0.67	6.75
NGFRAP1	NM_014380	−0.35	31.96	3.41	−28.56	−3.23	5.82
UPF3A	NM_023011	−0.31	18.45	1.27	−17.18	−3.86	5.63
CD36	NM_001127443	−0.28	47.21	11.74	−35.47	−2.01	5.53
MMP1	NM_002421	−0.31	69.30	31.02	−38.28	−1.16	5.38
RPA2	NM_002946	−0.63	16.56	1.76	−14.79	−3.23	5.05
GMPS	NM_003875	−0.29	13.70	1.24	−12.47	−3.47	5.03
STC1	NM_003155	−1.12	39.40	13.31	−26.09	−1.57	4.96
ACTL6A	NM_004301	−0.29	23.69	4.90	−18.79	−2.27	4.80
DCTN3	NM_024348	−0.48	19.61	3.29	−16.33	−2.58	4.78
LANCL1	NM_001136574	−0.63	8.61	0.67	−7.93	−3.68	4.74
PARP3	NM_001003931	−0.43	12.09	1.29	−10.80	−3.23	4.71
F3	NM_001993	−1.18	11.90	1.27	−10.63	−3.23	4.70
DCBLD2	NM_080927	−0.58	63.32	39.36	−23.96	−0.69	4.63
TPRG1L	NM_182752	−1.02	35.20	16.26	−18.95	−1.11	4.39
NDUFA4	NM_002489	−1.00	41.52	22.13	−19.39	−0.91	4.37
ADI1	NM_018269	−0.42	20.08	5.43	−14.65	−1.89	4.31
FBXO28	NM_001136115	−0.50	6.41	0.58	−5.83	−3.47	4.30
URGCP	NM_001077663	−0.31	7.87	0.84	−7.03	−3.23	4.28
SLC25A44	NM_014655	−0.23	7.84	0.84	−7.01	−3.23	4.28
FBXO28	NM_015176	−0.58	6.18	0.56	−5.62	−3.47	4.27
UBA6	NM_018227	−0.35	15.66	3.30	−12.37	−2.25	4.27
MAF1	NM_032272	−0.36	18.96	5.13	−13.83	−1.89	4.23
SQSTM1	NM_003900	−0.98	27.66	11.51	−16.15	−1.27	4.21
M6PR	NM_002355	−0.85	9.08	1.18	−7.90	−2.94	4.19
SAR1B	NM_016103	−1.28	14.67	3.25	−11.41	−2.17	4.13
ZBTB4	NM_020899	−1.05	12.64	2.55	−10.08	−2.31	4.05
UFD1L	NM_001035247	−0.98	10.48	1.75	−8.72	−2.58	4.05
SLC35A5	NM_017945	−0.75	7.93	1.03	−6.89	−2.94	4.05
RAD23B	NM_002874	−0.76	30.48	15.32	−15.17	−0.99	4.05
MITD1	NM_138798	−0.20	14.23	3.34	−10.89	−2.09	4.03
UFD1L	NM_005659	−0.99	10.16	1.70	−8.46	−2.58	4.02
PTP4A1	NM_003463	−0.46	20.89	7.77	−13.12	−1.43	3.98
RRM2	NM_001034	−0.73	7.13	0.93	−6.21	−2.94	3.95
SERPINB8	NM_002640	−0.52	6.91	0.90	−6.01	−2.94	3.92
SLC16A7	NM_004731	−0.51	6.58	0.86	−5.72	−2.94	3.87
GPR137B	NM_003272	−0.76	8.88	1.49	−7.39	−2.58	3.87
ERRFI1	NM_018948	−0.41	12.41	2.91	−9.50	−2.09	3.86
MKRN1	NM_013446	−1.08	12.33	2.89	−9.44	−2.09	3.86
NRBP1	NM_013392	−0.76	34.15	20.71	−13.44	−0.72	3.82
PFKP	NM_002627	−1.29	30.21	17.14	−13.07	−0.82	3.80
CYTSB	NM_001033553	−0.65	5.90	0.77	−5.13	−2.94	3.77
LSM5	NM_012322	−0.42	8.06	1.35	−6.71	−2.58	3.77
LYRM5	NM_001001660	−0.87	11.41	2.67	−8.73	−2.09	3.76
GTDC1	NM_001006636	−1.08	6.73	1.13	−5.60	−2.58	3.58
SLC39A6	NM_012319	−0.94	23.60	12.58	−11.02	−0.91	3.58
ELK3	NM_005230	−1.22	13.24	4.23	−9.01	−1.64	3.57
MYO10	NM_012334	−0.28	16.54	6.64	−9.90	−1.32	3.56
PURA	NM_005859	−1.02	14.18	5.16	−9.02	−1.46	3.49
TXNDC12	NM_015913	−0.64	14.57	5.70	−8.88	−1.36	3.43
MRPL24	NM_145729	−1.24	19.55	9.82	−9.73	−0.99	3.43
MGAT2	NM_002408	−0.21	14.33	5.60	−8.73	−1.36	3.41
ACIN1	NM_014977	−0.21	14.16	5.53	−8.63	−1.36	3.39
C4orf33	NM_001099783	−0.81	8.15	1.91	−6.24	−2.09	3.37
TXNIP	NM_006472	−1.27	27.42	17.63	−9.79	−0.64	3.35
COMMD10	NM_016144	−0.30	20.00	10.66	−9.34	−0.91	3.35
C1orf9	NM_014283	−1.06	9.61	2.68	−6.93	−1.84	3.35
CCNDBP1	NM_012142	−0.34	9.22	2.49	−6.73	−1.89	3.33
UBE2B	NM_003337	−0.93	10.83	3.46	−7.36	−1.64	3.32
NTN4	NM_021229	−1.21	23.64	14.28	−9.36	−0.73	3.31
RSU1	NM_012425	−0.21	15.87	7.28	−8.59	−1.12	3.30
CENPQ	NM_018132	−1.27	7.49	1.76	−5.74	−2.09	3.28
COPG2	NM_012133	−0.46	7.47	1.75	−5.72	−2.09	3.27
SLC35F5	NM_025181	−1.02	22.73	13.86	−8.87	−0.71	3.23
ABR	NM_021962	−0.34	9.37	2.89	−6.48	−1.70	3.18
RAB21	NM_014999	−0.43	18.73	10.40	−8.33	−0.85	3.17
TBP	NM_003194	−0.24	6.80	1.59	−5.20	−2.09	3.17
GNPDA2	NM_138335	−1.25	6.75	1.58	−5.17	−2.09	3.16
CDCA4	NM_145701	−0.22	6.72	1.58	−5.14	−2.09	3.16
ABTB1	NM_172027	−0.27	11.79	4.61	−7.18	−1.36	3.15
HMGN4	NM_006353	−0.78	6.58	1.54	−5.04	−2.09	3.13
AKTIP	NM_022476	−0.47	17.71	9.69	−8.02	−0.87	3.13
YIPF2	NM_024029	−0.25	22.38	14.13	−8.25	−0.66	3.12
MAGT1	NM_032121	−0.29	16.10	8.30	−7.79	−0.95	3.11
GOLGB1	NM_004487	−0.34	12.27	5.16	−7.11	−1.25	3.09
SFRS4	NM_005626	−0.24	13.30	6.00	−7.30	−1.15	3.09
DCAF16	NM_017741	−0.85	7.40	2.00	−5.40	−1.89	3.08
TIPARP	NM_015508	−0.40	12.20	5.27	−6.93	−1.21	3.04
CEP120	NM_153223	−1.19	7.14	1.93	−5.21	−1.89	3.04
CHD9	NM_025134	−1.00	7.91	2.38	−5.52	−1.73	3.01
KDSR	NM_002035	−0.29	10.46	4.09	−6.37	−1.36	3.00
ATMIN	NM_015251	−0.60	9.05	3.12	−5.93	−1.54	2.99
ZFP91	NM_053023	−0.61	11.31	4.77	−6.53	−1.24	2.98
LIN7B	NM_022165	−1.10	10.29	4.02	−6.27	−1.36	2.97
UBE3C	NM_014671	−0.54	14.49	7.62	−6.88	−0.93	2.93
ACBD5	NM_145698	−0.71	9.75	3.81	−5.94	−1.36	2.91
NAP1L1	NM_004537	−0.24	20.71	13.62	−7.09	−0.60	2.89
NBL1	NM_182744	−0.36	13.82	7.36	−6.45	−0.91	2.84
FGL2	NM_006682	−0.77	18.81	12.09	−6.71	−0.64	2.82
RAB11FIP5	NM_015470	−1.13	12.52	6.29	−6.23	−0.99	2.82
VPS26A	NM_001035260	−0.69	9.11	3.56	−5.55	−1.36	2.82
MAPKSP1	NM_021970	−0.30	9.07	3.55	−5.53	−1.36	2.81
RBL2	NM_005611	−1.07	10.07	4.35	−5.72	−1.21	2.79
PDZD11	NM_016484	−1.15	18.70	12.18	−6.52	−0.62	2.77
SSFA2	NM_006751	−0.80	12.51	6.45	−6.06	−0.95	2.77
VPS26A	NM_004896	−0.70	8.63	3.37	−5.26	−1.36	2.75
SSFA2	NM_001130445	−0.77	12.23	6.31	−5.92	−0.95	2.74
CRK	NM_005206	−0.91	12.79	6.82	−5.97	−0.91	2.73
DPM2	NM_003863	−0.26	11.72	5.89	−5.83	−0.99	2.73
PPP2R5E	NM_006246	−1.19	10.00	4.51	−5.49	−1.15	2.71
ARL1	NM_001177	−0.90	18.55	12.29	−6.26	−0.59	2.71
ATP2B1	NM_001682	−0.52	10.31	4.93	−5.39	−1.07	2.65
CRK	NM_016823	−0.93	11.88	6.33	−5.55	v0.91	2.63
ATP2B1	NM_001001323	−0.46	10.08	4.82	−5.27	−1.07	2.62
MGLL	NM_001003794	−0.24	16.69	10.87	−5.82	−0.62	2.61
DYNC1LI2	NM_006141	−1.14	16.12	10.50	−5.62	−0.62	2.57
RHOT1	NM_001033568	−0.34	11.74	6.42	−5.32	−0.87	2.56
RTCD1	NM_001130841	−0.62	10.85	5.78	−5.07	−0.91	2.51
DNM1L	NM_012062	−0.50	15.16	9.87	−5.28	−0.62	2.48

mir-100-5p (MIMAT0000098)							
Gene Symbol	RefSeq ID	mirSVR Score	SR	SEN	dRPKM	FC	D
RAP1B	NM_001010942	−1.14	55.53	30.38	−25.15	−0.87	4.73
FZD8	NM_031866	−1.14	7.31	0.95	−6.36	−2.94	3.97
EPDR1	NM_017549	−0.37	30.89	17.53	−13.37	−0.82	3.83
PL-5283	NM_001130929	−0.39	13.47	3.65	−9.83	−1.89	3.80
SIAH2	NM_005067	−1.15	6.92	1.16	−5.76	−2.58	3.61
TSC22D3	NM_198057	−0.60	14.84	6.69	−8.15	−1.15	3.24
C9orf123	NM_033428	−0.92	9.37	3.66	−5.71	−1.36	2.86

mir-125b-5p (MIMAT0000423)							
Gene Symbol	RefSeq ID	mirSVR Score	SR	SEN	dRPKM	FC	D
RPA2	NM_002946	−0.41	16.56	1.76	−14.79	−3.23	5.05
PRKRA	NM_003690	−0.22	15.60	1.66	−13.93	−3.23	4.99
STC1	NM_003155	−0.56	39.40	13.31	−26.09	−1.57	4.96
ACTL6A	NM_004301	−0.28	23.69	4.90	−18.79	−2.27	4.80
SNRPB	NM_003091	−0.62	29.19	7.90	−21.29	−1.89	4.80
TMEM50A	NM_014313	−0.44	43.86	19.78	−24.09	−1.15	4.73
PLIN3	NM_005817	−0.32	38.73	16.86	−21.86	−1.20	4.61
MLF2	NM_005439	−0.34	24.20	6.55	−17.65	−1.89	4.55
CCNC	NM_005190	−0.42	19.08	3.95	−15.13	−2.27	4.53
RABL6	NM_024718	−0.36	9.10	0.97	−8.13	−3.23	4.42
DSTN	NM_006870	−0.22	56.15	35.73	−20.41	−0.65	4.40
ANPEP	NM_001150	−0.32	58.88	39.09	−19.79	−0.59	4.35
ESRRA	NM_004451	−0.93	10.49	1.37	−9.13	−2.94	4.34
SLC35A5	NM_017945	−0.24	7.93	1.03	−6.89	−2.94	4.05
TIMM17B	NM_005834	−0.28	13.63	3.20	−10.43	−2.09	3.98
RRM2	NM_001034	−0.35	7.13	0.93	−6.21	−2.94	3.95
TRIB1	NM_025195	−0.30	6.42	0.84	−5.58	−2.94	3.85
HAX1	NM_006118	−0.25	19.83	7.75	−12.08	−1.36	3.84
TCTA	NM_022171	−0.26	8.44	1.41	−7.03	−2.58	3.82
ZNF828	NM_001164145	−0.96	6.17	0.80	−5.36	−2.94	3.81
KIAA0174	NM_014761	−0.65	20.94	9.05	−11.90	−1.21	3.77
OSBPL9	NM_148909	−1.22	9.94	2.33	−7.61	−2.09	3.60
NT5C3L	NM_052935	−0.45	16.39	6.40	−9.98	−1.36	3.59
TBC1D1	NM_015173	−0.40	10.47	2.67	−7.80	−1.97	3.56
PSMD9	NM_002813	−0.95	12.16	3.89	−8.27	−1.64	3.46
TXNIP	NM_006472	−0.45	27.42	17.63	−9.79	−0.64	3.35
PRRC1	NM_130809	−0.96	10.49	3.24	−7.25	−1.70	3.32
FIBP	NM_198897	−0.83	19.64	10.47	−9.17	−0.91	3.32
MRPL10	NM_145255	−0.21	7.40	1.74	−5.67	−2.09	3.26
TSC22D3	NM_198057	−0.71	14.84	6.69	−8.15	−1.15	3.24
MED15	NM_015889	−0.22	11.93	4.66	−7.27	−1.36	3.17
ABTB1	NM_172027	−0.30	11.79	4.61	−7.18	−1.36	3.15
DDX42	NM_203499	−0.48	16.50	8.51	−7.99	−0.95	3.15
ZSWIM6	NM_020928	−1.26	8.94	2.76	−6.18	−1.70	3.13
VPS4B	NM_004869	−1.11	11.50	4.49	−7.00	−1.36	3.12
GOLGB1	NM_004487	−0.23	12.27	5.16	−7.11	−1.25	3.09
OAZ2	NM_002537	−0.40	14.86	7.92	−6.94	−0.91	2.94
PSMG3	NM_001134340	−0.22	9.97	3.90	−6.07	−1.36	2.93
TAF9B	NM_015975	−1.04	8.67	3.39	−5.28	−1.36	2.76
HAS1	NM_001523	−0.35	16.13	10.18	−5.95	−0.66	2.66
MRPS10	NM_018141	−0.21	15.73	9.93	−5.80	−0.66	2.62
SLC39A9	NM_018375	−0.84	13.90	8.43	−5.47	−0.72	2.56
RTCD1	NM_001130841	−0.43	10.85	5.78	−5.07	−0.91	2.51

mir-92a-1-5p (MIMAT0004507)							
Gene Symbol	RefSeq ID	mirSVR Score	SR	SEN	dRPKM	FC	D
H2AFZ	NM_002106	−0.30	68.35	35.26	−33.09	−0.95	5.14
RAP1B	NM_001010942	−0.22	55.53	30.38	−25.15	−0.87	4.73
TMEM50A	NM_014313	−1.03	43.86	19.78	−24.09	−1.15	4.73
ATPIF1	NM_178191	−0.24	25.56	7.88	−17.67	−1.70	4.48
MEA1	NM_014623	−0.51	44.85	24.54	−20.31	−0.87	4.43
ZFAND3	NM_021943	−0.20	8.55	0.91	−7.63	−3.23	4.36
EGLN2	NM_053046	−0.37	10.38	1.35	−9.03	−2.94	4.33
LOC729991	NM_001145784	−0.46	12.65	2.12	−10.53	−2.58	4.26
NR3C1	NM_001020825	−0.64	26.80	10.96	−15.84	−1.29	4.19
PA2G4	NM_006191	−0.81	22.68	8.09	−14.59	−1.49	4.14
MRPS18B	NM_014046	−0.25	18.57	5.94	−12.63	−1.64	4.01
PTP4A1	NM_003463	−0.51	20.89	7.77	−13.12	−1.43	3.98
SERPINB8	NM_002640	−0.49	6.91	0.90	−6.01	−2.94	3.92
TCTA	NM_022171	−0.21	8.44	1.41	−7.03	−2.58	3.82
CYTSB	NM_001033553	−0.21	5.90	0.77	−5.13	−2.94	3.77
TPP1	NM_000391	−0.55	10.99	2.58	−8.42	−2.09	3.72
HIST1H2BM	NM_003521	−0.29	17.42	6.81	−10.61	−1.36	3.67
IP6K2	NM_001005911	−0.59	10.42	2.44	−7.98	−2.09	3.65
ABCF1	NM_001025091	−0.27	18.69	7.89	−10.80	−1.24	3.65
SIAH2	NM_005067	−0.63	6.92	1.16	−5.76	−2.58	3.61
OSBPL9	NM_148909	−0.54	9.94	2.33	−7.61	−2.09	3.60
IP6K2	NM_001146179	−0.56	9.89	2.32	−7.57	−2.09	3.59
C17orf49	NM_001142798	−0.24	20.07	10.08	−9.99	−0.99	3.47
AIDA	NM_022831	−0.21	24.98	15.15	−9.83	−0.72	3.38
DPT	NM_001937	−1.04	7.49	1.76	−5.74	−2.09	3.28
CCDC92	NM_025140	−0.33	7.22	1.69	−5.53	−2.09	3.23
NOL10	NM_024894	−0.59	14.08	6.08	−8.00	−1.21	3.23
MED15	NM_015889	−0.24	11.93	4.66	−7.27	−1.36	3.17
C20orf132	NM_213632	−0.24	11.75	4.59	−7.16	−1.36	3.15
AKTIP	NM_022476	−0.25	17.71	9.69	−8.02	−0.87	3.13
TPBG	NM_006670	−0.21	12.98	6.27	−6.71	−1.05	2.94
SRR	NM_021947	−0.95	9.41	3.68	−5.73	−1.36	2.86
POGK	NM_017542	−0.87	7.39	2.36	−5.03	−1.64	2.85
DDX24	NM_020414	−0.26	20.20	13.39	−6.81	−0.59	2.83
DPM2	NM_003863	−0.32	11.72	5.89	−5.83	−0.99	2.73

let-7a-2-3p (MIMAT0010195)							
Gene Symbol	RefSeq ID	mirSVR Score	SR	SEN	dRPKM	FC	D
IL8	NM_000584	−0.31	89.60	8.90	−80.69	−3.33	7.16
IL33	NM_033439	−1.22	35.40	1.12	−34.28	−4.98	7.13
RPS25	NM_001028	−1.27	268.93	142.20	−126.74	−0.92	7.05
SFRS13A	NM_054016	−0.29	14.40	0.99	−13.41	−3.86	5.38
NDUFB2	NM_004546	−0.37	68.00	30.66	−37.34	−1.15	5.35
H2AFZ	NM_002106	−0.26	68.35	35.26	−33.09	−0.95	5.14
RPA2	NM_002946	−0.24	16.56	1.76	−14.79	−3.23	5.05
SNRPB	NM_198216	−0.67	33.43	9.04	−24.38	−1.89	4.98
STC1	NM_003155	−1.19	39.40	13.31	−26.09	−1.57	4.96
FGFR1OP	NM_007045	−1.26	15.11	1.61	−13.50	−3.23	4.95
SRGN	NM_002727	−0.72	64.01	36.31	−27.70	−0.82	4.86
NME1	NM_198175	−0.30	52.74	26.50	−26.24	−0.99	4.82
SNRPB	NM_003091	−0.82	29.19	7.90	−21.29	−1.89	4.80
RAP1B	NM_001010942	−0.48	55.53	30.38	−25.15	−0.87	4.73
SERPINE2	NM_006216	−0.41	69.92	44.34	−25.57	−0.66	4.72
PARP3	NM_001003931	−0.66	12.09	1.29	−10.80	−3.23	4.71
F3	NM_001993	−0.29	11.90	1.27	−10.63	−3.23	4.70
ELOVL1	NM_022821	−0.40	36.66	14.33	−22.34	−1.36	4.68
TNFRSF10D	NM_003840	−0.26	57.91	33.52	−24.39	−0.79	4.68
ANKRD1	NM_014391	−0.21	30.17	10.76	−19.40	−1.49	4.53
BASP1	NM_006317	−0.59	50.43	28.72	−21.71	−0.81	4.51
WDR44	NM_019045	−0.40	8.12	0.73	−7.39	−3.47	4.51
TXNDC11	NM_015914	−0.23	9.35	1.00	−8.35	−3.23	4.45
MEA1	NM_014623	−1.13	44.85	24.54	−20.31	−0.87	4.43
ESRRA	NM_004451	−0.69	10.49	1.37	−9.13	−2.94	4.34
ADI1	NM_018269	−0.52	20.08	5.43	−14.65	−1.89	4.31
UBA6	NM_018227	−0.23	15.66	3.30	−12.37	−2.25	4.27
GOSR2	NM_054022	−0.48	12.34	2.07	−10.27	−2.58	4.24
CCDC51	NM_024661	−0.25	12.03	2.02	−10.02	−2.58	4.21
SAR1B	NM_016103	−0.38	14.67	3.25	−11.41	−2.17	4.13
COPS7A	NM_016319	−0.58	26.65	11.51	−15.14	−1.21	4.10
KCTD9	NM_017634	−0.27	26.65	11.60	−15.04	−1.20	4.09
RAD23B	NM_002874	−0.66	30.48	15.32	−15.17	−0.99	4.05
GNG5	NM_005274	−0.99	41.76	26.36	−15.40	−0.66	4.00
PTP4A1	NM_003463	−0.82	20.89	7.77	−13.12	−1.43	3.98
FZD8	NM_031866	−0.59	7.31	0.95	−6.36	−2.94	3.97
SERPINB8	NM_002640	−0.92	6.91	0.90	−6.01	−2.94	3.92
PDGFC	NM_016205	−0.41	12.91	3.03	−9.88	−2.09	3.91
GPR137B	NM_003272	−0.43	8.88	1.49	−7.39	−2.58	3.87
TRIB1	NM_025195	−0.38	6.42	0.84	−5.58	−2.94	3.85
PTPLAD1	NM_016395	−0.32	15.33	4.73	−10.60	−1.70	3.81
KIAA0174	NM_014761	−0.54	20.94	9.05	−11.90	−1.21	3.77
HMGN1	NM_004965	−0.24	33.75	20.95	−12.80	−0.69	3.74
BMP6	NM_001718	−0.26	22.51	10.75	−11.76	−1.07	3.71
DHX36	NM_020865	−0.64	10.76	2.52	−8.23	−2.09	3.69
TPM3	NM_001043352	−0.57	24.53	12.71	−11.82	−0.95	3.69
BAG3	NM_004281	−1.28	7.05	1.18	−5.87	−2.58	3.63
COIL	NM_004645	−1.02	6.88	1.15	−5.73	−2.58	3.60
WSB1	NM_015626	−0.94	22.86	11.79	−11.07	−0.95	3.60
DENR	NM_003677	−0.85	19.45	8.93	−10.53	−1.12	3.58
TBC1D1	NM_015173	−0.58	10.47	2.67	−7.80	−1.97	3.56
NAP1L1	NM_139207	−0.52	23.85	12.96	−10.89	−0.88	3.56
PAPSS2	NM_001015880	−0.94	20.80	10.23	−10.58	−1.02	3.55
SDC1	NM_002997	−0.48	18.60	8.53	−10.07	−1.12	3.52
PURA	NM_005859	−0.22	14.18	5.16	−9.02	−1.46	3.49
MPPE1	NM_023075	−0.80	6.13	1.03	−5.10	−2.58	3.49
AP3S1	NM_001284	−0.31	22.10	11.77	−10.32	−0.91	3.49
DCTN2	NM_006400	−1.31	22.62	12.38	−10.24	−0.87	3.47
SEC62	NM_003262	−0.46	16.23	6.96	−9.27	−1.22	3.44
SFRS13A	NM_006625	−0.29	17.03	7.68	−9.35	−1.15	3.42
DCAF12	NM_015397	−0.59	9.17	2.48	−6.69	−1.89	3.33
GM2A	NM_000405	−0.37	9.13	2.47	−6.66	−1.89	3.32
UBE2B	NM_003337	−0.41	10.83	3.46	−7.36	−1.64	3.32
FAM24A	NM_001029888	−0.23	13.03	5.09	−7.94	−1.36	3.28
PSMA5	NM_002790	−1.18	8.80	2.38	−6.42	−1.89	3.28
DEPDC7	NM_001077242	−0.23	7.38	1.73	−5.65	−2.09	3.26
RNF152	NM_173557	−1.23	7.17	1.68	−5.49	−2.09	3.23
RHBDL2	NM_017821	−1.28	7.05	1.65	−5.39	−2.09	3.21
ANP32B	NM_006401	−1.34	17.62	9.39	−8.23	−0.91	3.17
TROVE2	NM_004600	−0.92	11.98	4.68	−7.30	−1.36	3.17
TBP	NM_003194	−1.24	6.80	1.59	−5.20	−2.09	3.17
RPF1	NM_025065	−1.25	11.80	4.61	−7.19	−1.36	3.15
METTL3	NM_019852	−0.27	6.62	1.55	−5.07	−2.09	3.14
YTHDF2	NM_016258	−0.27	9.31	2.98	−6.33	−1.64	3.13
VPS4B	NM_004869	−0.77	11.50	4.49	−7.00	−1.36	3.12
SNX2	NM_003100	−1.21	11.27	4.40	−6.86	−1.36	3.09
SFRS4	NM_005626	−1.17	13.30	6.00	−7.30	−1.15	3.09
KLHL7	NM_001031710	−0.94	8.97	2.87	−6.10	−1.64	3.08
SPRY2	NM_005842	−0.31	11.04	4.32	−6.73	−1.36	3.07
INSIG1	NM_198337	−0.75	12.38	5.58	−6.80	−1.15	2.99
CSTF3	NM_001033506	−1.28	10.37	4.05	−6.32	−1.36	2.98
LIN7B	NM_022165	−0.61	10.29	4.02	−6.27	−1.36	2.97
STXBP1	NM_001032221	−0.24	10.11	3.95	−6.16	−1.36	2.95
MFSD1	NM_022736	−1.12	9.87	3.86	−6.01	−1.36	2.92
STXBP1	NM_003165	−0.22	9.79	3.83	−5.96	−1.36	2.91
PRKAR2A	NM_004157	−1.19	9.79	3.82	−5.96	−1.36	2.91
NAP1L1	NM_004537	−0.39	20.71	13.62	−7.09	−0.60	2.89
C1QBP	NM_001212	−1.34	20.04	13.05	−6.99	−0.62	2.87
SLC33A1	NM_004733	−0.22	7.48	2.39	−5.09	−1.64	2.87
INSIG1	NM_005542	−0.76	11.18	5.04	−6.14	−1.15	2.86
LZIC	NM_032368	−0.42	12.88	6.47	−6.41	−0.99	2.86
ANKRD17	NM_032217	−0.50	9.11	3.56	−5.55	−1.36	2.82
FMR1	NM_002024	−1.08	12.33	6.19	−6.13	−0.99	2.80
ATP2B1	NM_001682	−0.24	10.31	4.93	−5.39	−1.07	2.65
ATP2B1	NM_001001323	−0.20	10.08	4.82	−5.27	−1.07	2.62
FAHD1	NM_031208	−0.67	10.76	5.41	−5.35	−0.99	2.62
DYNC1LI2	NM_006141	−0.53	16.12	10.50	−5.62	−0.62	2.57
RHOT1	NM_001033568	−0.21	11.74	6.42	−5.32	−0.87	2.56

### Luciferase assay

The luciferase reporter constructs were built as previously described.^63^ NAP1L1-1 (350bp, 2713-3062) and NAP1L1-2 (675bp, 3362-5037) from the 3′ UTR of human *NAP1L1* gene, USP6-1 (675bp, 6220-6895) and USP6-2 (527bp, 7420-7945) from the 3′UTR of human *USP6* gene and SMARCD2 (525bp, 1913-2438) from the 3′ UTR of human *SMARCD2* gene were amplified using the primer sets (shown in Supplementary Experimental Procedures). Purified PCR products were cloned into multiple cloning sites of the pmirGLO dual-luciferase miRNA target expression vector (Promega) downstream of the firefly luciferase gene. The primer sequences were flanked by SacI and SalI sites to generate pmirGLO-*NAP1L1-1*, pmirGLO-*NAP1L1-2*, pmirGLO-*SMARCD2*, pmirGLO-*USP6-1* and pmirGLO-*USP6-2*. Details are given in Supplementary Experimental Procedures.

### Mimic miRNA transfection studies

The hADSCs (SR (PD8)) were seeded on 4-well slides at a density of 1 × 10^4^ cells/well 1 day before transfection with 5 and 10 pmol each of different miRNA mimics to SA-miRNA using Fugene 6 (Promega). 48 h after transfection, SA-β-Gal staining was performed according to manufacturer’s instructions (BioVision), RNA extraction and the subsequent real-time qPCR were performed to detect target gene expression.

### Statistical analysis

Data points from individual assays represent mean ± SEM. The statistical significance between two conditions was assessed by a two-tailed unpaired *t*-test. ^*^ *p* < 0.05, ^**^ *p* < 0.01, ^***^ *p* < 0.001, and n.s. represents *p* ≥ 0.05.

## Data access

The raw data files are being passed to NCBI’s Sequence Read Archive (SRA).Link from GEO records GSE77284 study at: http://www.ncbi.nlm.nih.gov/geo/query/acc.cgi?acc=GSE77284. A description of the data formats, software tools to manipulate these data formats, and all codes implementing the statistical models described herein can also be found in the supplementary information on line.

## Electronic supplementary material

Supplementary Methods

Supplementary Figure1

Supplementary Figure2

Supplementary Figure3

Supplementary Figure4

Supplementary Figure5

Supplementary Figure6

Supplementary Figure7

Supplementary Figure8

Supplementary Figure9

Supplementary Figure legends

Supplementary Table1

Supplementary Table2

Supplementary Table3

Supplementary Table4

## Electronic supplementary material

Supplementary Information accompanies the paper on the *npj Aging and Mechanisms of Disease* website (doi:10.1038/s41514-017-0006-y).
