# Supplementary material for: Opposing activities of oncogenic MIR17HG and tumor suppressive MIR100HG clusters and their gene targets regulate replicative senescence in human adult stem cells
Source: NPJ Aging Mech Dis. 2017 Apr 20;3:7. doi: 10.1038/s41514-017-0006-y (PMC5460214; doi:10.1038/s41514-017-0006-y)
Supplement: Supplementary file 1 — Supplementary Methods [file 41514_2017_6_MOESM1_ESM.docx]

**SUPPLEMENTARY ON LINE EXPERIMENTAL PROCEDURES**.

**Antibodies**

Primary antibodies used were: 53BP1 (Bethyl Laboratories #A300-273A), BrdU (Bu20a, # 5292, Cell Signalling), PML (N-19; Santa Cruz), P21*^WAF1/CIP^* (# 8587, Cell Signalling) γH2AX (Millipore #05-636, Millipore) CD31 (Invitrogen #HMCD3101), CD44 (Invitrogen #HMCD4401), CD45 (Invitrogen #HMCD4501), CD105 (Invitrogen #HMCD10520) Secondary antibodies were AlexaFlour® conjugated donkey antibodies (Invitrogen and TermoFisher).

**Immunofluorescence**

1–3×10^4^ cells/well in 4-well slides were fixed with 4% paraformaldehyde and permeabilized with PBS, 0.5% Triton X-100. The blocking and antibody incubations were performed in 4% normal donkey serum (NDS; Jackson Immunochemicals) in PBS. The nuclei were counter-stained with 100 ng/ml 4′, 6-diamidino-2-phenylindole (DAPI; Sigma), and the slides were mounted in ProLong® Gold anti-fade aqueous mounting medium (Invitrogen). Epifluorescence images were acquired on an Olympus BX60 fluorescence microscope with Spotfire 3.2.4 software (Diagnostics Instruments).

**Proteomic Analysis**

***Sample Preparation***

Pellets from approximately 10 ^8^ cells were either SR or SEN lysed in 0.4 ml of lysis buffer (8M Gu-HCL + DTT). Samples were subsequently alkylated with 45mM Iodoacetic acid (500mM stock concentration in 1 M Ammonium Bicarbonate) in the dark for 1 hr at room temperature. Residual alkylation agent was then reacted with 15 mM DTT. Samples were then diluted with 25 mM TrisHCl 5 mM CaCl2 to 2.5mL and added to a glass vial of trypsin (Pierce, 20ug, in 250uL of 25mM acetic acid). Samples were allowed to digest for 20 hrs at room temperature. Samples were quenched with formic acid and introduced into the mass spectrometer.

***Liquid Chromatography and High-resolution Mass Spectrometry***

Stem cell samples were prepared as described above and 1ug was injected onto a Thermo Scientific Easy nLC system configured with a 10 cm x 100 um trap column and a 25 cm x 100 um ID resolving column. Buffer A was 98% water, 2% methanol, and 0.2% formic acid. Buffer B was 10% water, 10% isopropanol, 80% acetonitrile, and 0.2% formic acid. Samples were loaded at 4 uL/min for 10 min, and a gradient from 0-45% B at 375 nL/min was run over 130 min, for a total run time of 150 min (including regeneration and sample loading). The Thermo Scientific LTQ Orbitrap Velos mass-spectrometer was run in a standard Top-10 data-dependent configuration except that a higher trigger threshold (20 K) was used to ensure that the MS2 did not interfere with the full-scan duty cycle. This ensured optimal full-scan data for quantification. MS2 fragmentation and analysis were performed in the ion trap mass analyzer. Samples were run in triplicate.

***MS Data Analysis***

Protein identification was performed using ThermoScientific Proteome Discoverer version 1.4 (including Sequest and Percolator algorithms) using the RefSeqHuman sequence database. The Percolator peptide confidence filter was set to “high”. Protein quantification was performed using Pinpoint version 1.4 software. The Pinpoint quantification workflow included importing the Proteome Discoverer.msf files as spectral libraries. Identified peptides were subsequently quantified in MS.raw files using the Pinpoint peak finding, chromatographic alignment, and area calculation algorithms.

***LC-MS/MS Proteome Expression Analysis***

Peptide expression levels are taken as the total area under the LC-MS/MS relative intensity curve (Supplementary Figure 5A), and individual peptides were unambiguously assigned to proteins using the Pinpoint software, version 1.4 (ThermoScientific) as described above. The areas *A* of all peptides *i* assigned to an individual protein *I* were summed to yield raw (non-normalized) protein expression levels: $I= \sum_{i=1}^{n} A_{i}$.

The raw protein expression levels *I* for each individual library, characterized as described in the previous section, were normalized against the total size of the library. For each protein *i* from library *j*, the normalized expression level *I’* is calculated as: $I^{'}= \frac{I_{i,j}}{\sum_{k=1}^{N} I_{k}}$, where *N* is the total number of proteins from library *j* (Supplementary Figure 5). Normalized protein expression levels for the three individual SR libraries and the three individual SEN libraries were compared using the Student’s *t-*test with a *P*-value cutoff of 0.05 to identify proteins that are differentially expressed between SR and SEN hADSCs.

***Transcriptome Analysis with RNA-seq***

Characterization of gene expression levels with RNA-seq was performed on replicates of self-renewing (SR) and senescent (SEN) human adult adipose- derived mesenchymal stem cells (hADSCs) using the Roche 454 sequencing platform. Individual sequence reads were mapped to the human genome reference sequence (UCSC hg18; NCBI build 36.1) using the program BLAT. (Kent 2002) BLAT was used in light of the relatively long sequence reads provided by 454 (avg=216bp; Supplementary Figure 3A), and the program was run with default settings with the exception that the minimal sequence identity was set to 99%. Ties between multi-mapping sequence reads were broken by selecting the mapping location where the read was maximally covered by NCBI RefSeq annotated exons.

Analysis of differential gene expression levels between SR and SEN cells was performed using an approach adopted from a recently developed method that was designed to be accurate at the relatively low sequencing depth provided by 454 and for single replicate experiments (Tarazona, Garcia-Alcalde et al. 2011). Our adoption of this approach employs a combination of two-parameters in order to define differential expression levels between genes: 1) the difference in the number reads *per* kilobase *per* million mapped reads ($dRPKM$) and 2) the expression fold-change ($FC$) level. This approach controls for liabilities of each individual metric; in particular, $dRPKM$ is biased towards highly expressed genes, whereas $FC$ is biased towards lowly expressed genes. In this approach, $dRPKM$ is defined as: ${RPKM}_{SR}-{RPKM}_{SEN}$, and $FC$ is defined as: $\log_{2} {{RPKM}_{SR}}/{{RPKM}_{SEN}}$. For each locus, $dRPKM$ and $FC$ are plotted as a point in two-dimensional Euclidean space, and the Euclidean Distance ($D$) between the origin and the point is taken to represent the differential expression level. This approach was used separately to evaluate the differential expression of mRNAs and non-coding RNAs, including miRNAs, which are typically expressed at lower levels. For each class of RNA, empirical distributions of $Ed$ were evaluated to call genes as differentially expressed. For non-coding RNAs, differentially expressed genes are considered as those with |*FC*| > 0.95 and |*dRPKM*| > 4.07, and for mRNAs differentially expressed genes are considered as those with|*FC*| > 0.58 and |*dRPKM*| > 2.32 (Supplementary Figure 3B).

***Network-based Functional Enrichment Analysis***

The set of genes that were characterized as both targets of SEN upregulated miRNAs (Figure 2) and found to be downregulated in SEN hADSCs were manually analyzed based on functional annotations in the STRING database (Szklarczyk, Franceschini et al. 2011). Proteins from four annotation categories of interest – cell cycle, chromatin, transcription/translation and histone methyltransferases – were selected for functional enrichment analysis using a network-based approach. The network enrichment approach developed and applied here yields function-specific sub-networks based on the functional interactions in the STRING database, with edge confidence levels >0.4. For each set of functionally annotated proteins, a Steiner tree was built; the Steiner tree is the minimal spanning tree that connects all of the functionally annotated seed proteins by introducing the fewest number of intermediate proteins (*i.e.* Steiner nodes). Functional enrichment for these sub-networks was evaluated *via* the implementation of a previously described simulation approach (Talkowski, Rosenfeld et al. 2012). For each function-specific sub-network, the observed score (*NS*) is computed $NS=G/T$ where *G* is the number of functionally annotated seed proteins and *T* is the total number of proteins in the network. A null set of expected NS scores is then simulated by randomly selecting *G* seed proteins from the same underlying degree distribution and then constructing the Steiner tree of size *T* from these random seeds. The *P*-value for each sub-network is computed via a *z*-test comparing the observed *NS* score versus the expected *NS* score distribution.

***RT-PCR***

Primers were designed by Primer3 software and shown in Table 1.

**Table 1. mRNA qPCR primers**

| Gene | Forward Primer | Reverse Primer |
| --- | --- | --- |
| NAP1L1 | 5’ CTGGCTCCCCATACTAGTCG 3’ | 5’CTTGAAGGGCTGCAAGAATC 3’ |
| USP6 | 5’ ACCATCACAGGCTCTTCA CC 3’ | 5’ AACGATCAATGCTGCTGTTG 3’ |
| SMARCD2 | 5’ ACCCCATTGTCATCAACCAT 3’ | 5’ TCTCTGGGTCTTCAGCTGGT 3’ |
| CHD2 | 5’GATGACGAAGCTCCCAAAG 3’ | 5’TAGATGCTCCAGTGGCTCCT 3’ |
| CHD4 | 5’CATCGATGGTGGAATCACTG 3’ | 5’ATCCGGTGAGCTCTGCTAAA 3’ |
| CHD8 | 5’ATGCGGATTGTGAAGAAGGA3’ | 5’GGCTCTTCATCCTCATGGAA3’ |
| HDAC3 | 5’TGGCTTCTGCTATGTCAACG 3’ | 5’TCTCTGCCCCGACTTCATAC3’ |
| HDAC5 | 5’TCTGAACCACTGCATTTCCA 3’ | 5’GCCTGGACCGTAATTTCAGA 3’ |
| HDAC9 | 5’CAGGCGGAAGGATGGAAATG3’ | 5’ATGCGTTGCTGTGAAACCAT3’ |
| WDR44 | 5’TCTCTCCTAACCGCAAGCAT3’ | 5’AGCTCTCTCCCAGAGTTGGA 3’ |
| SAP18 | 5’CCACTGTTGCTACGGGTCTT 3’ | 5’CCACTGTTGCTACGGGTCTT 3’ |
| SUZ12 | 5’GCCTTTGAGAAGCCAACACA3’ | 5’CTGCAAATGAGCTGACAAGC3’ |
| SMARCA1 | 5’AGGGCGAGAAGAAGAAGGAG3’ | 5’TCTGTGCTGAAGGCTGAATG3’ |
| IGF2BP3 | 5’TCCAAGCAGAAACCATGTGA3’ | 5’ACTTACAAGCCGCAGAGGTG3’ |

***Luciferase assay***

**Table 2. Luciferase vector pmirGLO construction primers**

| 3’UTR of gene | Primers |
| --- | --- |
| NAP1L1-1  (2713-3062) | Forward 5’ CCC GAG CTC GCT TAA AGT ATG AGT ATGTCA CT 3’  Reverse 5’ CCC GTC GAC AAA ACA AAT CTT GGA CCT TGT GA 3’ |
| NAP1L1-2  (3362-5037) | Forward 5’ CCC GAG CTC TGA AGC AGT ATT AGC ATC ACT3’  Reverse 5’ CCC GTC GAC TAT TAT TTC ACC ATC ACC ATT TAC A 3’ |
| SMARCD2  (1913-2438) | Forward 5’ CCC GAG CTC CTG CTC AGG GAT CTT TCT TCC C 3’  Reverse 5’ CCC GTC GAC AAA AAA AGT GGC TCC CAC ATA GA 3’ |
| USP6-1  (6220-6895) | Forward 5’CCC GAG CTC ATA TGT AGT GAG TAT AGA GTT TAC CCA A 3’  Reverse 5’ CCC GTC GAC TTT GCA TGT GTT CTC TCT TTT TTA AAG T3’ |
| USP6-2  (7420-7945) | Forward 5’ CCC GAG CTC AAA TTG AAA TCC TTT TCA GAA AAA A 3’  Reverse 5’ CCC GTC GAC AAA AAC AGC ACA TAG AGG C 3’ |

The ready-to-use microRNA mimics are small, double-stranded RNA molecules designed to mimic endogenous mature microRNA (miRNA) molecules. When transfected into cells, they can regulate gene expression in different manners, including translational repression, mRNA cleavage, and deadenylation, imitating the native miRNA. The relative luciferase activity was measured as previously described (Anbazhagan, Priyamvada et al. 2014). 1X10^4^ 293T cells were seeded per well into 96-well plates one day before transfection with 500ng pmirGLO/pmirGLO-UTR constructs alone or in combination with 1 pmol different microRNA mimics to SA-miRNA (Sigma, MISSION^®^ microRNA Mimic), using Fugene6 (Promega) according to manufacturer’s instructions. Forty-eight hours post-transfection, cells were lysed in a passive lysis buffer (Promega). The luciferase activity was then determined using the Dual Luciferase Assay Kit (Promega). Renilla luciferase activity was used as a control. Subsequently, the firefly luciferase activity was normalized to renilla luciferase activity. The 3’-UTR activity was calculated as a ratio of firefly luciferase to renilla luciferase. All of the experiments were repeated three times.
