# Supplementary figures and images for: Opposing activities of oncogenic MIR17HG and tumor suppressive MIR100HG clusters and their gene targets regulate replicative senescence in human adult stem cells
Source: NPJ Aging Mech Dis. 2017 Apr 20;3:7. doi: 10.1038/s41514-017-0006-y (PMC5460214; doi:10.1038/s41514-017-0006-y)

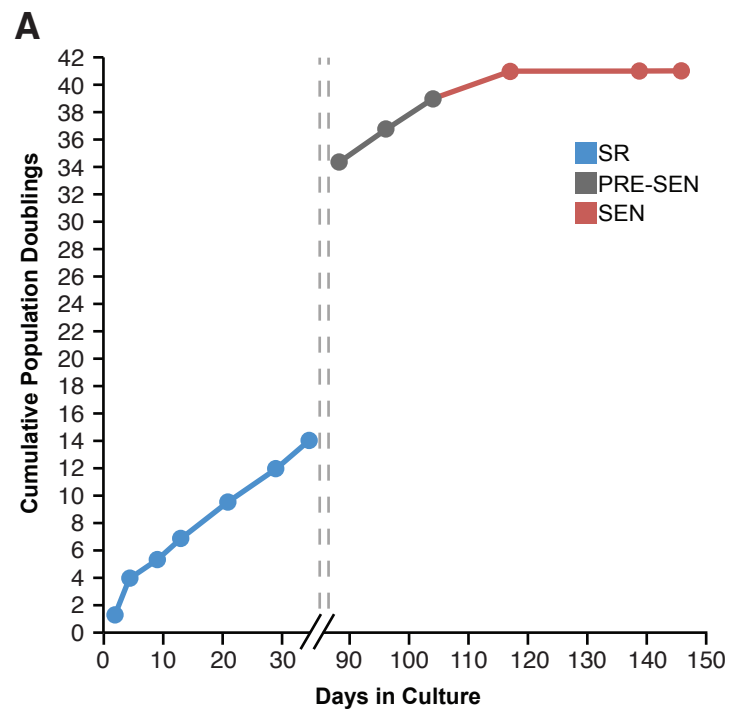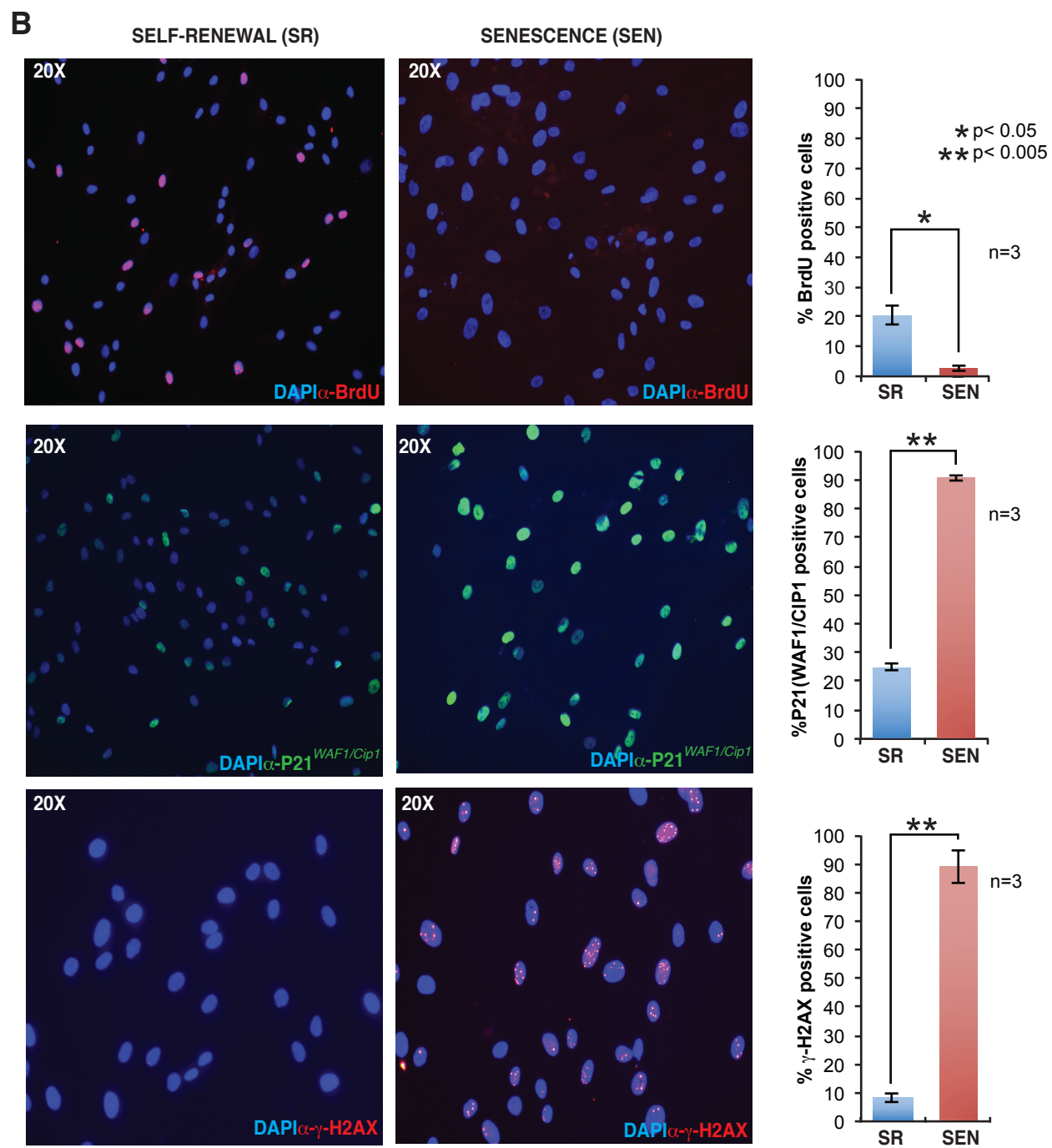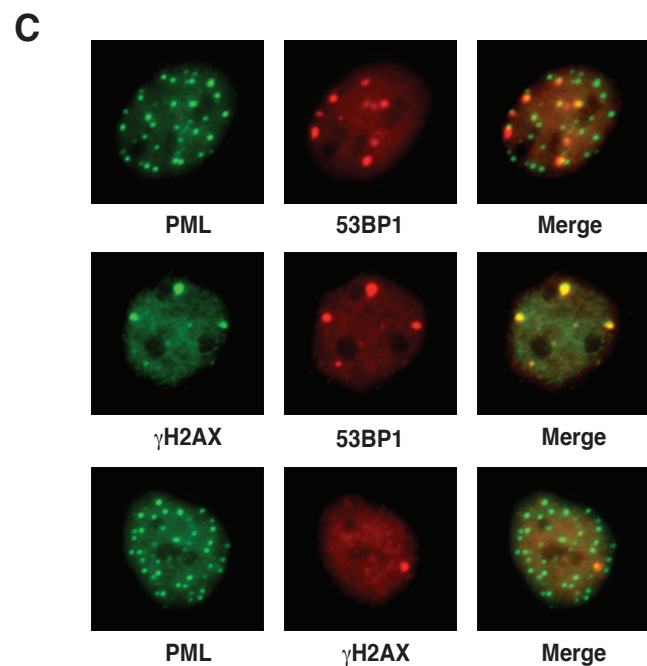

Supl. Fig.1

Supplement: Supplementary file 2 — Supplementary Figure1 [file 41514_2017_6_MOESM2_ESM.pdf]

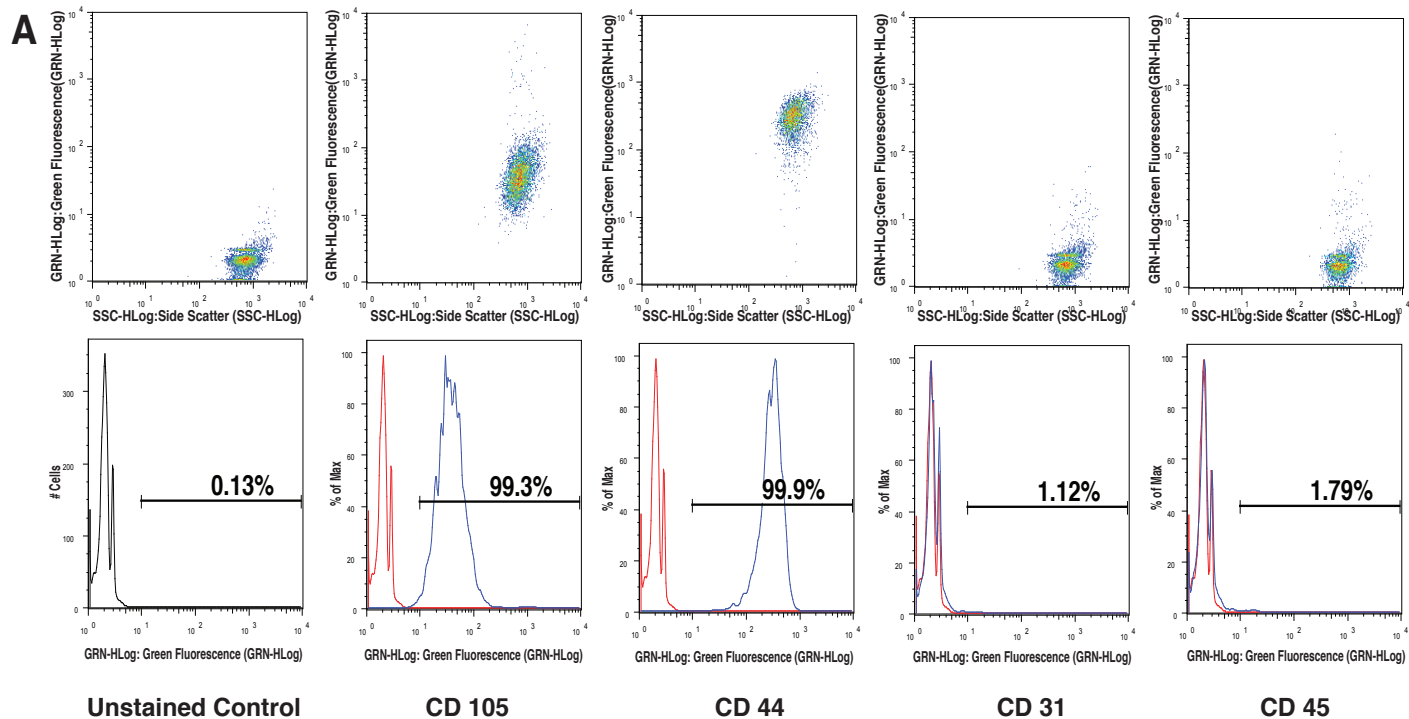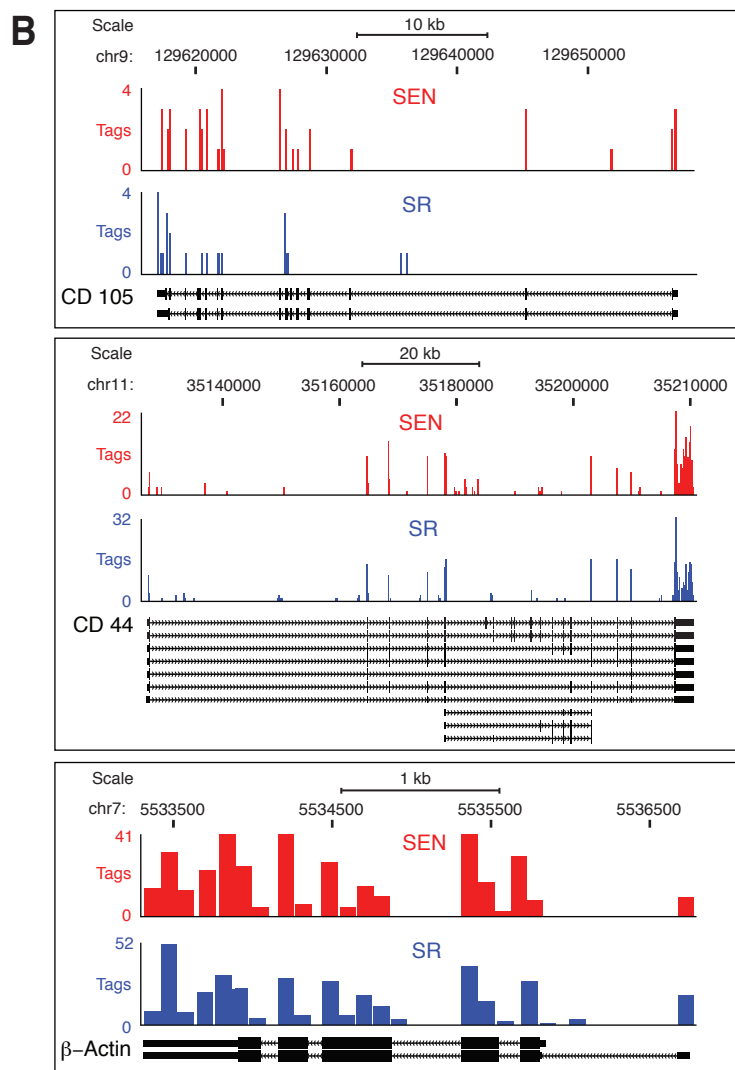

Supl. Fig.2

Supplement: Supplementary file 3 — Supplementary Figure2 [file 41514_2017_6_MOESM3_ESM.pdf]

**A**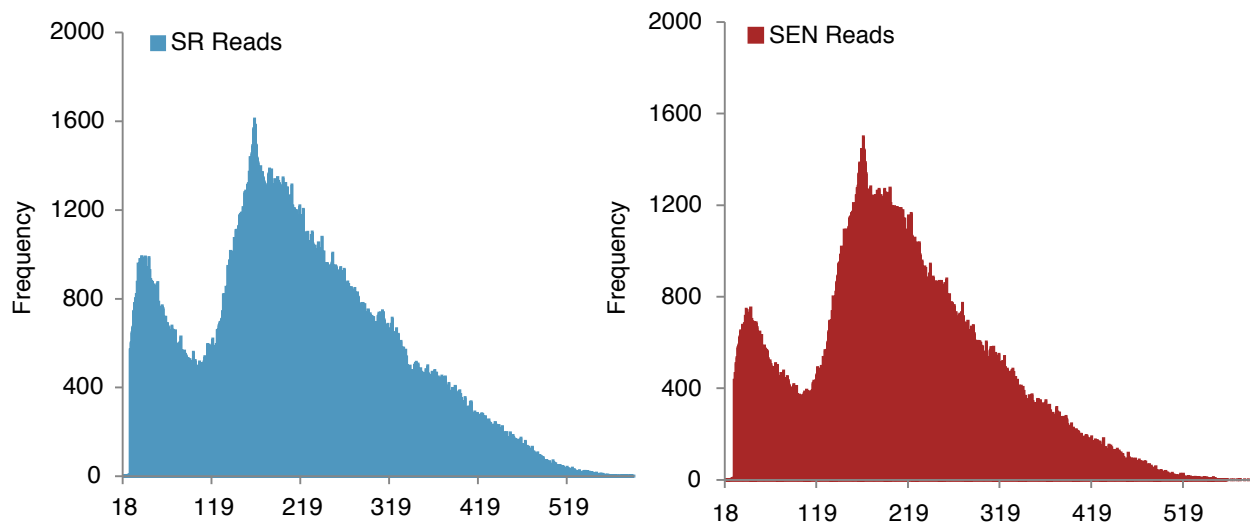**B**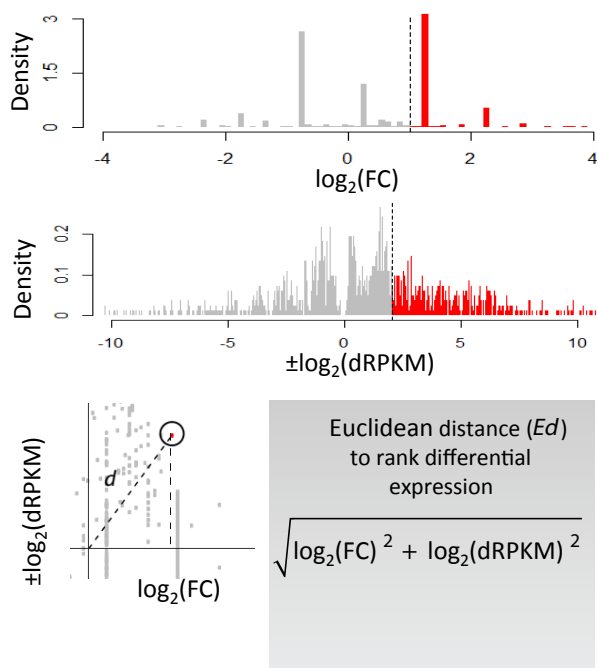**C**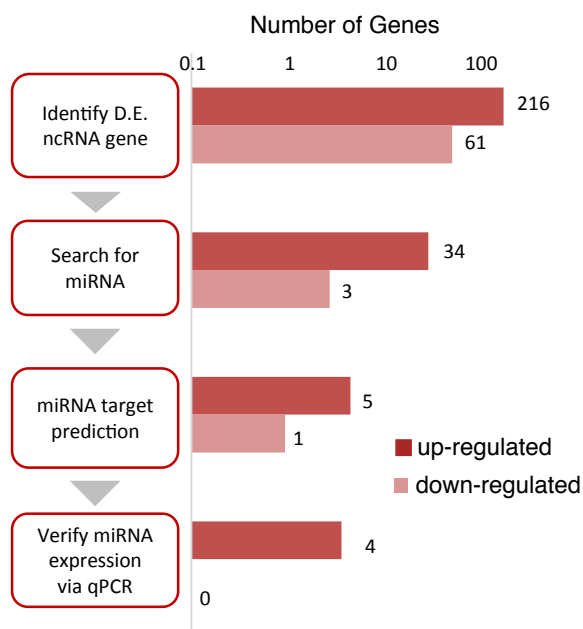

Supl. Fig. 3

Supplement: Supplementary file 4 — Supplementary Figure3 [file 41514_2017_6_MOESM4_ESM.pdf]

**A**

qPCR analysis of microRNA

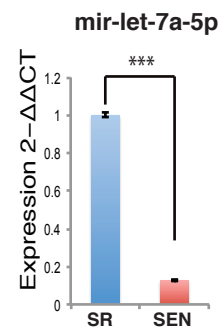**B**

Protein Level

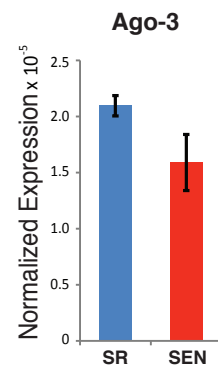

Supplement: Supplementary file 5 — Supplementary Figure4 [file 41514_2017_6_MOESM5_ESM.pdf]

**A**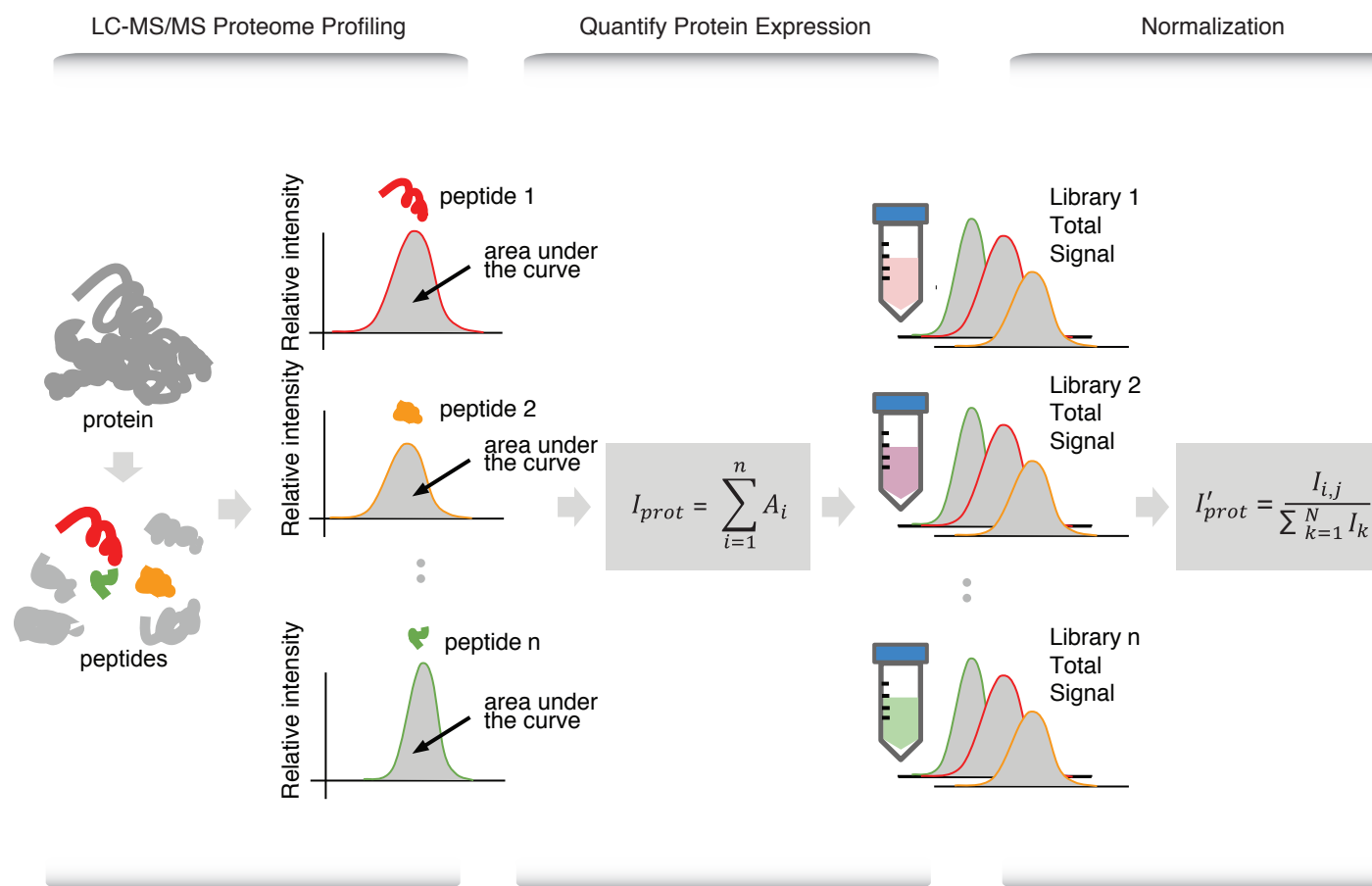**B**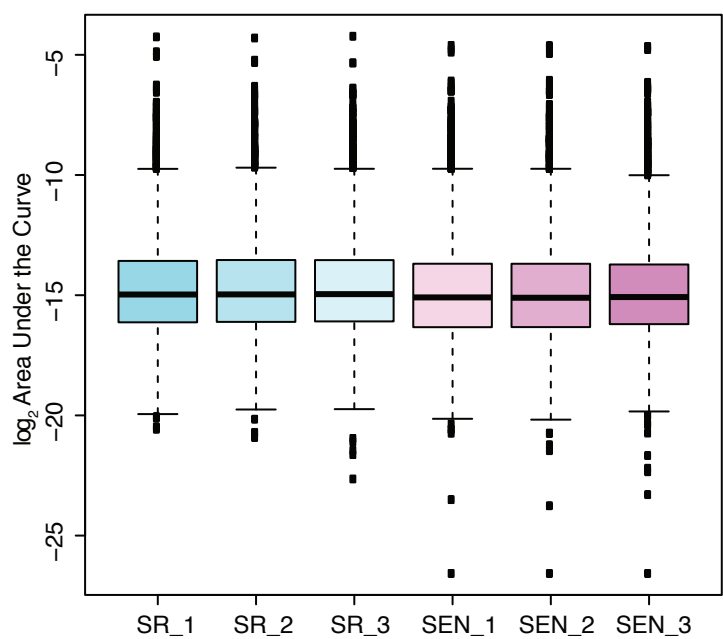**C**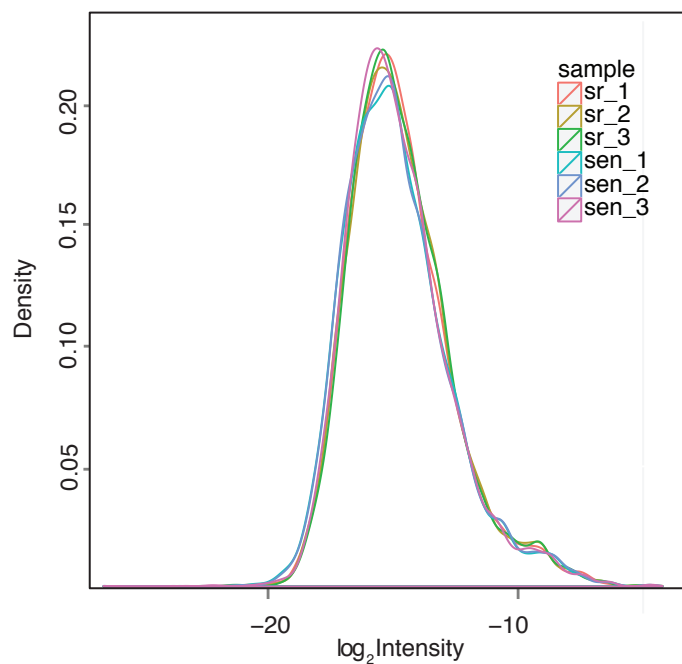

Supplement: Supplementary file 6 — Supplementary Figure5 [file 41514_2017_6_MOESM6_ESM.pdf]

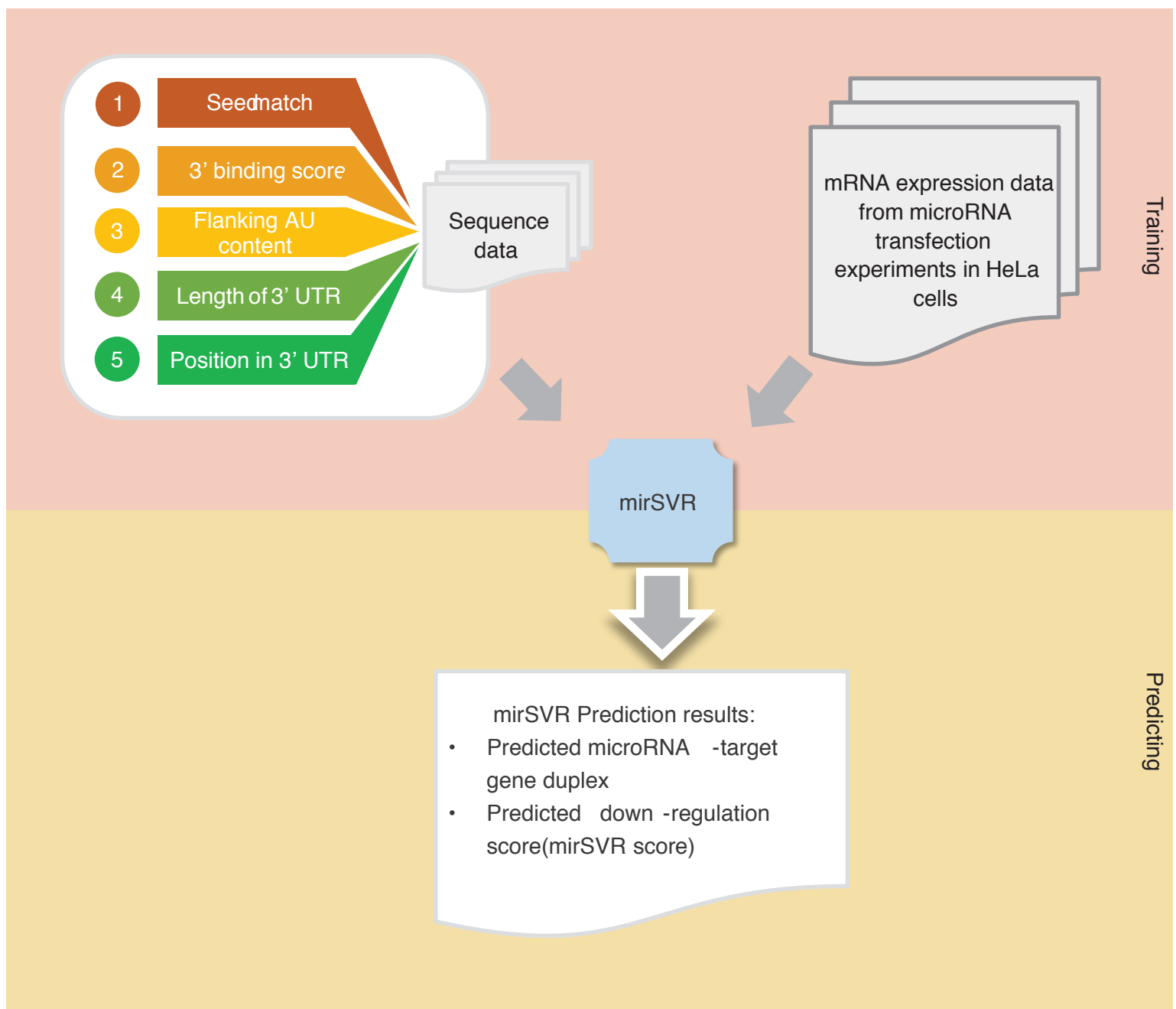

Suppl. Fig.6

Supplement: Supplementary file 7 — Supplementary Figure6 [file 41514_2017_6_MOESM7_ESM.pdf]

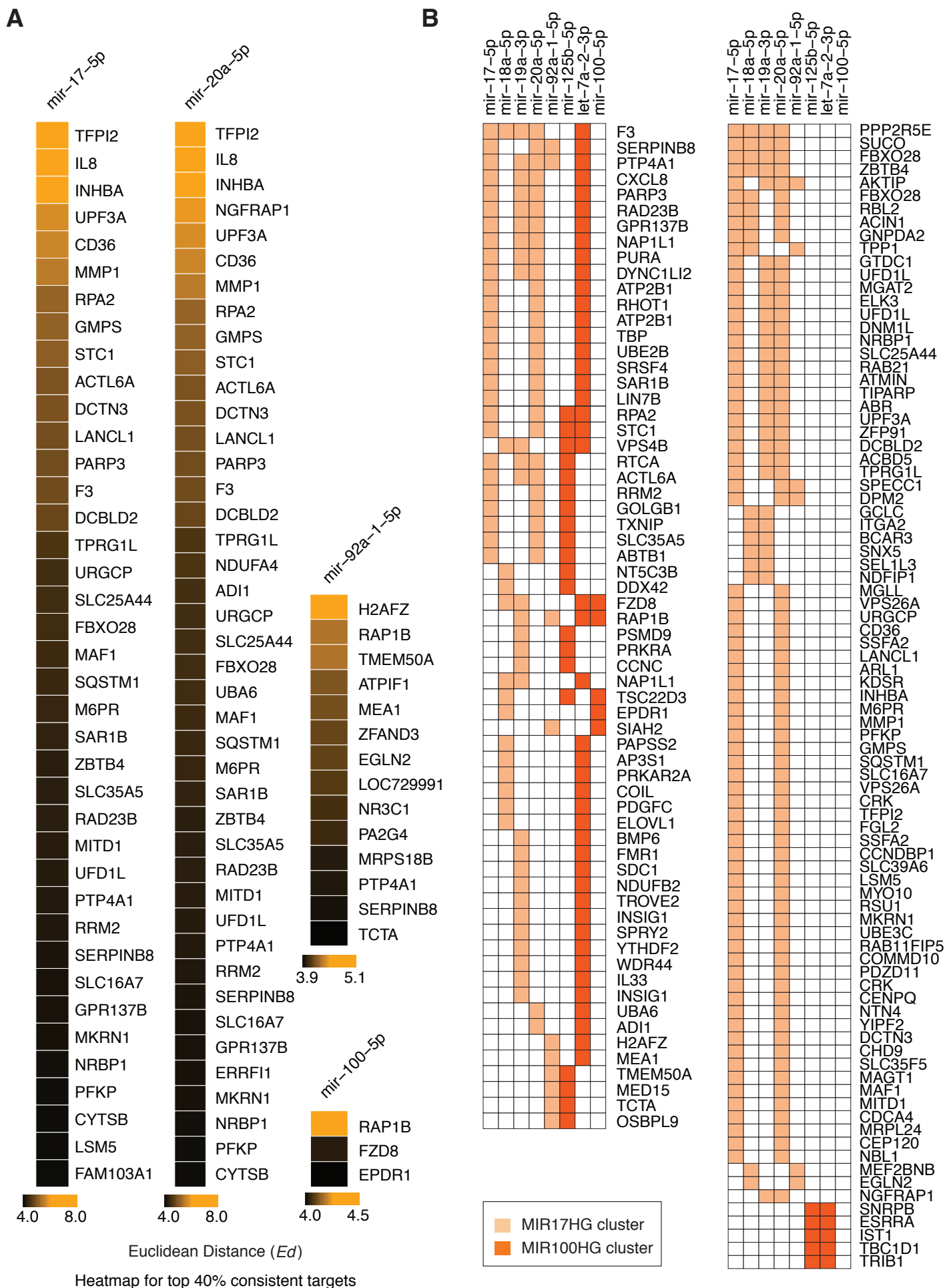

Suppl. Fig.8

Supplement: Supplementary file 9 — Supplementary Figure8 [file 41514_2017_6_MOESM9_ESM.pdf]
