# Supplementary material for: Opposing activities of oncogenic MIR17HG and tumor suppressive MIR100HG clusters and their gene targets regulate replicative senescence in human adult stem cells
Source: NPJ Aging Mech Dis. 2017 Apr 20;3:7. doi: 10.1038/s41514-017-0006-y (PMC5460214; doi:10.1038/s41514-017-0006-y)
Supplement: Supplementary file 8 — Supplementary Figure7 [file 41514_2017_6_MOESM8_ESM.pdf]

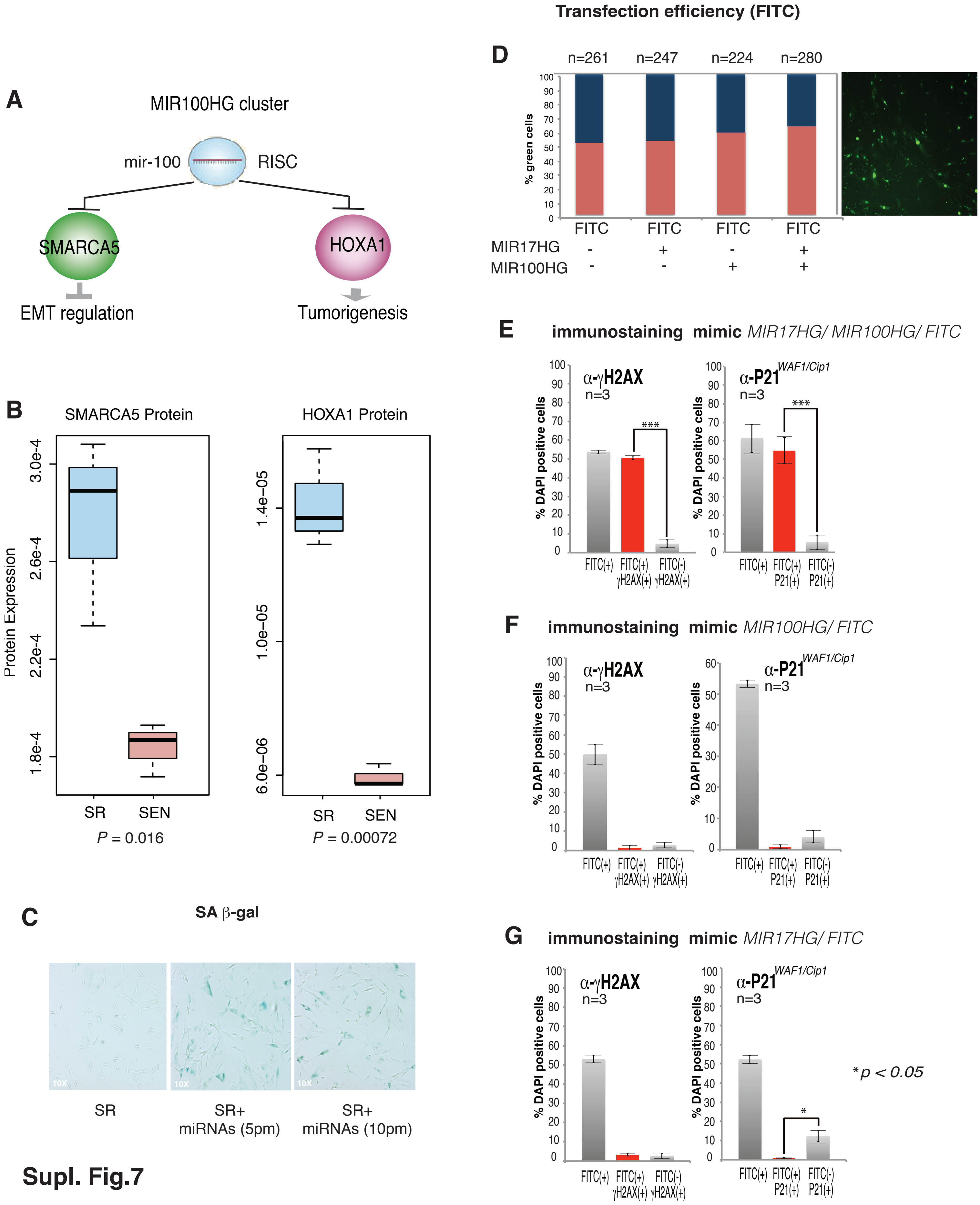
A
MIR100HG cluster
mir-100
RISC
SMARCA5
HOXA1
EMT regulation
Tumorigenesis
B
SMARCA5 Protein
HOXA1 Protein
Protein Expression
3.0e-4
2.6e-4
2.2e-4
1.8e-4
1.4e-05
1.0e-05
6.0e-06
SR
SEN
P = 0.016
P = 0.00072
C
SA β-gal
10X
SR
SR+ miRNAs (5pm)
SR+ miRNAs (10pm)
Transfection efficiency (FITC)
D
n=261
n=247
n=224
n=280
% green cells
FITC
MIR17HG
MIR100HG
E
immunostaining mimic MIR17HG/ MIR100HG/ FITC
α-γH2AX
α-P21 WAF1/Cip1
n=3
% DAPI positive cells
***
FITC(+)
FITC(+) γH2AX(+)
FITC(-) γH2AX(+)
FITC(+) P21(+)
FITC(-) P21(+)
F
immunostaining mimic MIR100HG/ FITC
G
immunostaining mimic MIR17HG/ FITC
*
*p < 0.05

Supl. Fig.7
