# Supplementary material for: Opposing activities of oncogenic MIR17HG and tumor suppressive MIR100HG clusters and their gene targets regulate replicative senescence in human adult stem cells
Source: NPJ Aging Mech Dis. 2017 Apr 20;3:7. doi: 10.1038/s41514-017-0006-y (PMC5460214; doi:10.1038/s41514-017-0006-y)
Supplement: Supplementary file 11 — Supplementary Figure legends [file 41514_2017_6_MOESM11_ESM.docx]

**SUPPLEMENTARY FIGURES LEGENDS**

**Supplementary Figure S1**. ***Ex-vivo* senescence of hADSCs is associated with formation of persistent DNA damage foci and** P21^WAF1/Cip1^ **upregulation.** (A) Representative growth curve of hADSCs. Two distinct states are shown: SR- self-renewing (population doubling <12); SEN- senescent (population doubling >39), gray line depicts preSEN- pre-senescent (population doubling 29-39) that were not included in our analysis. Cumulative population doublings PD were described in *^13^*. (B) Immunohistochemical detection of 5’-bromo-2’deoxyuridine (BrdU) incorporation, P21^WAF1/Cip1^ and γH2AX in senescent (SEN) and self-renewing (SR) populations of hADSCs. Examples (10X and 20X magnification) are shown in inserts. DNA was detected with DAPI. Quantification of senescent phenotype in hADSC: bar graphs correspond to a percentage of BrdU, P21^WAF1/Cip1^ and γH2AX positive cells in DAPI-stained total cell population, based on three independent experiments (n=3). Total amount of the cells counted in three experiments: BrdU staining- (SR) *n_1_*=61, *n_2_*=62, *n_3_*=76 (SEN) *n_1_*=56, *n_2_*=133, *n_3_*=88; P21^WAF1/Cip1^ staining- (SR: *n_1_*=193, *n_2_*=143, *n_3_*=179) (SEN: *n_1_*=180, *n_2_*=156, *n_3_*=173); γH2AX immunostaining- (SR *n_1_*=207, *n_2_*=208, *n_3_*=152) (SEN *n_1_*=142, *n_2_*=215, *n_3_*=142). Bars are the standard deviations from the mean. (C) DNA damage response (DDR) in senescent (SEN) hADSCs. Representative immunostaining for the persistent DNA damage response upon senescence of hADSCs. Representative image of a single nucleus is shown. Spatial relationship between 53BP1(red) and PML bodies (green), γH2AX (green) and 53BP1 (red) and PML bodies (green) and γH2AX (red) confirms the formation of persistent DNA damage foci associated with hADSCs senescence.

**Supplementary Figure S2**. **Immunophenotype of hADSCs upon senescence.** (A) Representative FACS analysis of hADSCs. SR (PD8) hADSCs were stained with FITC (CD 31, CD44 and CD 45) or AlexaFlour-488 (CD105) conjugated antibodies against cell surface markers and subjected to flow cytometry analysis. The cell populations are shown as fluorescence to side scatter graphs (top), and the histograms (bottom) of stained cells (blue line) compared to unstained cells (red line); with the percentage of positive cells indicated. (B) Representative analysis of *CD105*, *CD 44* and *β-actin* gene transcription by RNa-seq (SR (PD8), SEN (PD40)). Sequencing tracks are uploaded to the UCSC genome browser and details of the analysis are shown in the on line Experimental Procedures and Supplementary Table S3.

**Supplementary Figure S3**. **RNA-seq analysis for evaluating differential gene expression between SR versus SEN hADSCs.** (A) Length distributions of RNA-seq reads for SR (blue) and SEN (red) hADSCs. The distributions are bimodal owing to the RNA isolation approach used to enrich for small non-coding RNA species (see Experimental Procedures). (B) The approach used to identify SEN upregulated genes. A combination of fold-change (*FC*) and RPKM differences (*dRPKM*) was used as described in on line Experimental Procedures. *FC* and *dRPKM* distributions are shown along with the empirically determined cut-offs above which genes are considered to be SEN upregulated (red). *FC* and *dRPKM* are jointly analyzed with the Euclidean distance (E*d*) to quantify the extent of differential expression. (C) The overall scheme used to find differentially expressed miRNAs, which have mRNA target predictions from the mirSVR program, is shown along with the corresponding numbers of miRNAs identified at each step.

**Supplementary Figure S4**. **Mir-let7a-5p and AGO-3 analysis in hADSCs upon senescence.** (A) qPCR analysis of mature miRNA expression in self-renewing (SR, blue bar) and senescent (SEN, red bar) states of hADSCs. Relative expression guide strand of mature microRNA mir-let7a- (depicted in the graphs as *-5p*) to U6 small RNA was measured. Data are shown as fold change $\left( \Delta\Delta∁\tau\right).$ Mean$\pm$ SD from three independent experiments is shown. The statistical difference was evaluated by Student’s *t-*test and *P-*values (*p*) related to experimental measurements are listed under the graphs, where ****p* *< 0.001*. (B) Protein levels of AGO-3 protein measured in the proteomic study as described in on line Experimental Procedures. Differential expression of proteins upon self-renewal (SR) and senescence (SEN) of hADSCs. Normalized protein expression levels are shown for three replicate samples each of SR versus SEN hADSCs.

**Supplementary Figure S5. Proteomic analysis scheme for evaluating differential expression between SR versus SEN hADSCs.** (A) Schematic illustrating the proteomic analysis workflow and the approach used to quantify and normalize protein expression levels based on the LC-MS/MS proteome profiling. (B) Distributions of normalized protein expression levels for the three SR and three SEN libraries analyzed here.

**Supplementary Figure S6.** **Overview of the approach used to predict miRNA targets *via* the mirSVR program.** The mirSRV program’s approach to identifying potential miRNA targets is distinguished by its use of mRNA expression data from miRNA transfection experiments in HeLa cells along with sequence and contextual data for miRNA-mRNA seed matching regions.

**Supplementary Figure S7**. **Regulation of SMARCA5 and HOXA1 by the MIR100HG cluster and assessment of senescence-associated markers in transfection experiments with mimic miRNAs.** (A) Schematic of previously demonstrated *mir-100* regulation of SMARCA5 and HOXA1 along with the downstream functional effects. (B) SR versus SEN protein expression levels for SMARCA5 and HOXA1. Protein expression values are shown for three replicates each for SR and SEN along with the significance of the differences (Student’s *t-*test). (C) Representative field depicting SA-β-Gal positivity of self-renewing hADSCs (SR) and self-renewing hADSCs after transfection by 5pM or 10pM of the full set of mimics of SA-miRNAs (SR+miRNAs). (D) The transfection efficiency was assessed by co-transfecting mimics with FITC-labelled control as described in on line Experimental Procedures. Green cells were calculated 72 hrs after transfection of the mimics of MIR17HG (mir-17-5p, mir-18a-5p, mir-19a-3p, mir-20a-5p and mir-92a1-5p) or the MIR100HG (mir-125b1-5p, mir-1let7a-2-3p, mir-100-5p) clusters separately or after simultaneous transfection by the full set of SA-miRNA mimics from both clusters in SR hADSCs. DAPI was used for staining cell nuclei. Transfection efficiency for each combination is expressed as a percentage of green cells among the total amount of cells (DAPI) (n) counted under the fluorescent microscope. (E) Quantitation of the cell containing γH2AX persistent DDR focal staining in SA-miRNA mimics-transfected SR hADSCs (FITC+) at 72 hrs post-transfection presented as FITC^+^γH2AX^+^ (red bar). Quantification of FITC^+^  (red bar) and FITC^-^ cells positive for senescence-associated markers P21^WAF1/Cip1^ staining is performed in similar fashion. Results were plotted on graphs as averages of three independent experiments (biological replicates n=3) with the standard deviation of data. Amount of total cell counted in each experiments; γH2AX staining *n_1_*=117, *n_2_*=109, *n_3_*=133; P21^WAF1/Cip1^ staining *n_1_*=118, *n_2_*=135, *n_3_*=90. (F) Quantitation of cells containing γH2AX persistent DDR focal staining in MIR100HG mimics-transfected SR hADSCs (FITC+) at 72 hrs post-transfection is presented as FITC^+^γH2AX^+^ (red bar). Quantification of cells positive for senescence-associated markers P21^WAF1/Cip1^ staining is performed in separate experiments under the same conditions in FITC^+^ and FITC^-^ cells. Results were plotted on graphs as averages of three independent experiments (biological replicates n=3) with the standard deviation of data. Amount of total cell counted in each experiments; γH2AX staining *n_1_*=112, *n_2_*=90, *n_3_*=111; P21^WAF1/Cip1^ staining *n_1_*=117, *n_2_*=145, *n_3_*=224. (G) Similar quantifications as in (F) were performed in SR hADSCs transfected with MIR17HG mimics. Results were plotted on graphs as averages of three independent experiments (biological replicates n=3) with the standard deviation of data. Amount of total cell counted in each experiments; γH2AX staining *n_1_*=80, *n_2_*=90, *n_3_*=113; P21^WAF1/Cip1^ staining *n_1_*=134, *n_2_*=147, *n_3_*=144. Statistical differences in (E), (F) and (G) for senescence–associated markers comparisons in FITC^+^ and FITC^-^ cells were evaluated by Student’s *t*-test, where ****p* *< 0.0001*, **p* *<0.05*.

**Supplementary Figure S8**. **Downregulation of genes via SA-miRNA-based mRNA degradation.** (A) SEN downregulated protein-coding mRNAs targeted by SA-miRNAs. Differential expression levels are quantified by the Euclidean distance (*Ed*) as described in on line Experimental Procedures and Supplementary Figure S3. (B) SA-miRNA seed analysis in downregulated upon senescence mRNAs reveled that these genes might be regulated by coordinate action of multiple SA-miRNAs from two independent clusters. Individual miRNAs are shown and color-coded according to their miRNA gene cluster.

**Supplementary Figure S9**. **Downregulation of SEN proteins via miRNA-based translational repression.** (A) SEN down-regulated proteins targeted by SA-miRNAs. Differential protein expression is quantified by the Students’ *t-*test (-log_10_ *P*-values shown) as described in on line Experimental Procedures. (B) Coordinate regulation of SEN downregulated proteins by multiple SA-miRNAs. Individual miRNAs are shown and color-coded according to their miRNA gene cluster.

**Supplementary Table S1**. **List of mRNA targets downregulated in SEN hADSCs.** Data are shown for targets of eight SA-miRNAs from the MIR17HG and MIR100HG clusters. For each downregulated target, a gene symbol and NCBI Reference Sequence identifier are shown along with a mirSVR score and expression data. The mirSVR score indicates the predicted downregulation of the target gene by its cognate miRNA; lower scores indicate stronger predictions. RPKM expression levels are shown for SR and SEN hADSCs along with the expression level differences (*dRPKM*) for SEN-SR and the fold-change (*FC*=log_2_SEN/SR). *Ed* is the Euclidean distance measure of differential expression described in on line Experimental Procedures and Supplementary Figure S3.

**Supplementary Table S2**. **List of protein targets downregulated in SEN hADSCs.** Data are shown for targets of eight SA-miRNAs from the MIR17HG and MIR100HG clusters, and for each downregulated target a gene symbol and NCBI Reference Sequence identifier are shown along with a mirSVR score and expression data. Protein expression levels are shown as the mean normalized values (n=3 replicates), and the difference in the mean normalized values (Diff Mean=SEN-SR) are shown. *P*-values were computed using the Students’ *t-*test as described in on line Experimental Procedures.

**Supplementary Table S3**. **Results of the RNA-seq transcriptomic analysis.** Gene symbols and Reference Sequence identifiers are shown for all genes analyzed along with the RPKM expression levels for SR and SEN hADSCs. Expression level differences (*dRPKM*) for SEN-SR and fold-change (*FC*=log_2_SEN/SR) values are shown for all genes along with *Ed*, which is the Euclidean distance measure of differential expression described in on line Experimental Procedures and Supplementary Figure S3.

**Supplementary Table S4**. **Results of the proteomic analysis.** Gene symbols and Reference Sequence identifiers are shown for all proteins analyzed along with the mean normalized protein expression levels (n=3 replicates) for SR and SEN hADSCs. Differences in the mean normalized protein expression levels (Diff Mean=SEN-SR) are shown along with *P*-values computed using the Students’ *t-test* as described in on line Experimental Procedures and shown in the Figure 3C heatmap.
