# Supplementary material for: Opposing activities of oncogenic MIR17HG and tumor suppressive MIR100HG clusters and their gene targets regulate replicative senescence in human adult stem cells
Source: NPJ Aging Mech Dis. 2017 Apr 20;3:7. doi: 10.1038/s41514-017-0006-y (PMC5460214; doi:10.1038/s41514-017-0006-y)
Supplement: Supplementary file 12 — Supplementary Table1 [file 41514_2017_6_MOESM12_ESM.pdf]

Table S1 mRNA Targets Down-regulated in SEN

mir-17-5p (MIMAT0000070)							
**Gene Symbol**	**RefSeq ID**	**mirSVR Score**	**SR**	**SEN**	**dRPKM**	**FC**	**D**
TFPI2	NM_006528	-0.25	528.90	225.72	-303.17	-1.23	8.34
IL8	NM_000584	-1.18	89.60	8.90	-80.69	-3.33	7.16
INHBA	NM_002192	-0.36	282.13	177.25	-104.88	-0.67	6.75
UPF3A	NM_023011	-0.31	18.45	1.27	-17.18	-3.86	5.63
CD36	NM_001127443	-0.28	47.21	11.74	-35.47	-2.01	5.53
MMP1	NM_002421	-0.33	69.30	31.02	-38.28	-1.16	5.38
RPA2	NM_002946	-0.64	16.56	1.76	-14.79	-3.23	5.05
GMPS	NM_003875	-0.29	13.70	1.24	-12.47	-3.47	5.03
STC1	NM_003155	-1.12	39.40	13.31	-26.09	-1.57	4.96
ACTL6A	NM_004301	-0.29	23.69	4.90	-18.79	-2.27	4.80
DCTN3	NM_024348	-0.48	19.61	3.29	-16.33	-2.58	4.78
LANCL1	NM_001136574	-0.63	8.61	0.67	-7.93	-3.68	4.74
PARP3	NM_001003931	-0.44	12.09	1.29	-10.80	-3.23	4.71
F3	NM_001993	-1.18	11.90	1.27	-10.63	-3.23	4.70
DCBLD2	NM_080927	-0.58	63.32	39.36	-23.96	-0.69	4.63
TPRG1L	NM_182752	-1.03	35.20	16.26	-18.95	-1.11	4.39
FBXO28	NM_001136115	-0.50	6.41	0.58	-5.83	-3.47	4.30
URGCP	NM_001077663	-0.31	7.87	0.84	-7.03	-3.23	4.28
SLC25A44	NM_014655	-0.23	7.84	0.84	-7.01	-3.23	4.28
FBXO28	NM_015176	-0.58	6.18	0.56	-5.62	-3.47	4.27
MAF1	NM_032272	-0.35	18.96	5.13	-13.83	-1.89	4.23
SQSTM1	NM_003900	-0.98	27.66	11.51	-16.15	-1.27	4.21
M6PR	NM_002355	-0.85	9.08	1.18	-7.90	-2.94	4.19
SAR1B	NM_016103	-1.28	14.67	3.25	-11.41	-2.17	4.13
ZBTB4	NM_020899	-1.05	12.64	2.55	-10.08	-2.31	4.05
UFD1L	NM_001035247	-0.98	10.48	1.75	-8.72	-2.58	4.05
SLC35A5	NM_017945	-0.76	7.93	1.03	-6.89	-2.94	4.05
RAD23B	NM_002874	-0.76	30.48	15.32	-15.17	-0.99	4.05
MITD1	NM_138798	-0.20	14.23	3.34	-10.89	-2.09	4.03
UFD1L	NM_005659	-0.99	10.16	1.70	-8.46	-2.58	4.02
PTP4A1	NM_003463	-0.46	20.89	7.77	-13.12	-1.43	3.98
RRM2	NM_001034	-0.73	7.13	0.93	-6.21	-2.94	3.95
SERPINB8	NM_002640	-0.55	6.91	0.90	-6.01	-2.94	3.92
SLC16A7	NM_004731	-0.51	6.58	0.86	-5.72	-2.94	3.87
GPR137B	NM_003272	-0.76	8.88	1.49	-7.39	-2.58	3.87
MKRN1	NM_013446	-1.08	12.33	2.89	-9.44	-2.09	3.86
NRBP1	NM_013392	-0.76	34.15	20.71	-13.44	-0.72	3.82
PFKP	NM_002627	-1.29	30.21	17.14	-13.07	-0.82	3.80

CYTSB	NM_001033553	-0.65	5.90	0.77	-5.13	-2.94	3.77
LSM5	NM_012322	-0.42	8.06	1.35	-6.71	-2.58	3.77
FAM103A1	NM_031452	-0.89	11.30	2.65	-8.65	-2.09	3.75
TPP1	NM_000391	-0.68	10.99	2.58	-8.42	-2.09	3.72
GTDC1	NM_001006636	-1.08	6.73	1.13	-5.60	-2.58	3.58
SLC39A6	NM_012319	-0.94	23.60	12.58	-11.02	-0.91	3.58
ELK3	NM_005230	-1.22	13.24	4.23	-9.01	-1.64	3.57
MYO10	NM_012334	-0.28	16.54	6.64	-9.90	-1.32	3.56
VPS45	NM_007259	-0.40	6.43	1.08	-5.36	-2.58	3.54
PURA	NM_005859	-1.02	14.18	5.16	-9.02	-1.46	3.49
MRPL24	NM_145729	-1.24	19.55	9.82	-9.73	-0.99	3.43
MGAT2	NM_002408	-0.21	14.33	5.60	-8.73	-1.36	3.41
ACIN1	NM_014977	-0.21	14.16	5.53	-8.63	-1.36	3.39
TXNIP	NM_006472	-1.27	27.42	17.63	-9.79	-0.64	3.35
COMMD10	NM_016144	-0.30	20.00	10.66	-9.34	-0.91	3.35
C1orf9	NM_014283	-1.12	9.61	2.68	-6.93	-1.84	3.35
CCNDBP1	NM_012142	-0.34	9.22	2.49	-6.73	-1.89	3.33
UBE2B	NM_003337	-0.93	10.83	3.46	-7.36	-1.64	3.32
NTN4	NM_021229	-1.22	23.64	14.28	-9.36	-0.73	3.31
RSU1	NM_012425	-0.21	15.87	7.28	-8.59	-1.12	3.30
CENPQ	NM_018132	-1.27	7.49	1.76	-5.74	-2.09	3.28
SLC35F5	NM_025181	-1.02	22.73	13.86	-8.87	-0.71	3.23
ABR	NM_021962	-0.34	9.37	2.89	-6.48	-1.70	3.18
RAB21	NM_014999	-0.43	18.73	10.40	-8.33	-0.85	3.17
TBP	NM_003194	-0.24	6.80	1.59	-5.20	-2.09	3.17
GNPDA2	NM_138335	-1.25	6.75	1.58	-5.17	-2.09	3.16
CDCA4	NM_145701	-0.22	6.72	1.58	-5.14	-2.09	3.16
ABTB1	NM_172027	-0.27	11.79	4.61	-7.18	-1.36	3.15
AKTIP	NM_022476	-0.47	17.71	9.69	-8.02	-0.87	3.13
YIPF2	NM_024029	-0.25	22.38	14.13	-8.25	-0.66	3.12
MAGT1	NM_032121	-0.29	16.10	8.30	-7.79	-0.95	3.11
GOLGB1	NM_004487	-0.34	12.27	5.16	-7.11	-1.25	3.09
SFRS4	NM_005626	-0.24	13.30	6.00	-7.30	-1.15	3.09
TIPARP	NM_015508	-0.40	12.20	5.27	-6.93	-1.21	3.04
CEP120	NM_153223	-1.19	7.14	1.93	-5.21	-1.89	3.04
CHD9	NM_025134	-1.00	7.91	2.38	-5.52	-1.73	3.01
KDSR	NM_002035	-0.29	10.46	4.09	-6.37	-1.36	3.00
ATMIN	NM_015251	-0.60	9.05	3.12	-5.93	-1.54	2.99
ZFP91	NM_053023	-0.61	11.31	4.77	-6.53	-1.24	2.98
LIN7B	NM_022165	-1.10	10.29	4.02	-6.27	-1.36	2.97
UBE3C	NM_014671	-0.54	14.49	7.62	-6.88	-0.93	2.93
ACBD5	NM_145698	-0.71	9.75	3.81	-5.94	-1.36	2.91
NAP1L1	NM_004537	-0.25	20.71	13.62	-7.09	-0.60	2.89

NBL1	NM_182744	-0.35	13.82	7.36	-6.45	-0.91	2.84
FGL2	NM_006682	-0.77	18.81	12.09	-6.71	-0.64	2.82
RAB11FIP5	NM_015470	-1.13	12.52	6.29	-6.23	-0.99	2.82
VPS26A	NM_001035260	-0.69	9.11	3.56	-5.55	-1.36	2.82
RBL2	NM_005611	-1.08	10.07	4.35	-5.72	-1.21	2.79
PDZD11	NM_016484	-1.15	18.70	12.18	-6.52	-0.62	2.77
SSFA2	NM_006751	-0.80	12.51	6.45	-6.06	-0.95	2.77
NFAT5	NM_138713	-0.21	9.93	4.34	-5.59	-1.20	2.76
VPS26A	NM_004896	-0.71	8.63	3.37	-5.26	-1.36	2.75
SSFA2	NM_001130445	-0.77	12.23	6.31	-5.92	-0.95	2.74
CRK	NM_005206	-0.90	12.79	6.82	-5.97	-0.91	2.73
DPM2	NM_003863	-0.26	11.72	5.89	-5.83	-0.99	2.73
PPP2R5E	NM_006246	-1.19	10.00	4.51	-5.49	-1.15	2.71
ARL1	NM_001177	-0.90	18.55	12.29	-6.26	-0.59	2.71
ATP2B1	NM_001682	-0.50	10.31	4.93	-5.39	-1.07	2.65
CRK	NM_016823	-0.93	11.88	6.33	-5.55	-0.91	2.63
ATP2B1	NM_001001323	-0.44	10.08	4.82	-5.27	-1.07	2.62
MGLL	NM_001003794	-0.24	16.69	10.87	-5.82	-0.62	2.61
DYNC1LI2	NM_006141	-1.14	16.12	10.50	-5.62	-0.62	2.57
RHOT1	NM_001033568	-0.34	11.74	6.42	-5.32	-0.87	2.56
RTCD1	NM_001130841	-0.43	10.85	5.78	-5.07	-0.91	2.51
DNM1L	NM_012062	-0.53	15.16	9.87	-5.28	-0.62	2.48

mir-18a-5p (MIMAT0000072)							
**Gene Symbol**	**RefSeq ID**	**mirSVR Score**	**SR**	**SEN**	**dRPKM**	**FC**	**D**
TSPYL2	NM_022117	-0.30	665.60	381.06	-284.54	-0.80	8.19
HIST1H4H	NM_003543	-0.81	62.31	8.12	-54.19	-2.94	6.47
BCAR3	NM_003567	-1.25	15.52	0.96	-14.56	-4.02	5.57
MSRB2	NM_012228	-0.38	15.73	1.68	-14.05	-3.23	5.00
NDUFC2	NM_004549	-0.75	26.15	6.13	-20.02	-2.09	4.80
F3	NM_001993	-0.34	11.90	1.27	-10.63	-3.23	4.70
ELOVL1	NM_022821	-0.72	36.66	14.33	-22.34	-1.36	4.68
PCYT1A	NM_005017	-0.79	21.26	5.75	-15.51	-1.89	4.38
EGLN2	NM_053046	-0.35	10.38	1.35	-9.03	-2.94	4.33
FBXO28	NM_001136115	-0.57	6.41	0.58	-5.83	-3.47	4.30
FBXO28	NM_015176	-0.65	6.18	0.56	-5.62	-3.47	4.27
MEF2BNB	NM_001145784	-0.30	12.65	2.12	-10.53	-2.58	4.26
UQCRQ	NM_014402	-0.79	24.50	9.58	-14.93	-1.36	4.13
ZBTB4	NM_020899	-1.16	12.64	2.55	-10.08	-2.31	4.05
B4GALT7	NM_007255	-0.48	10.49	1.76	-8.74	-2.58	4.05
FZD8	NM_031866	-0.47	7.31	0.95	-6.36	-2.94	3.97

PDGFC	NM_016205	-1.17	12.91	3.03	-9.88	-2.09	3.91
EPDR1	NM_017549	-0.26	30.89	17.53	-13.37	-0.82	3.83
TPP1	NM_000391	-0.41	10.99	2.58	-8.42	-2.09	3.72
COIL	NM_004645	-0.23	6.88	1.15	-5.73	-2.58	3.60
CA12	NM_001218	-0.59	18.89	8.40	-10.49	-1.17	3.59
NT5C3L	NM_052935	-0.24	16.39	6.40	-9.98	-1.36	3.59
NAP1L1	NM_139207	-0.21	23.85	12.96	-10.89	-0.88	3.56
PAPSS2	NM_001015880	-0.99	20.80	10.23	-10.58	-1.02	3.55
AP3S1	NM_001284	-1.26	22.10	11.77	-10.32	-0.91	3.49
ACIN1	NM_014977	-0.24	14.16	5.53	-8.63	-1.36	3.39
SNX5	NM_014426	-0.27	16.36	7.38	-8.98	-1.15	3.37
C1orf9	NM_014283	-1.04	9.61	2.68	-6.93	-1.84	3.35
NAE1	NM_003905	-1.34	12.85	5.02	-7.83	-1.36	3.26
TSC22D3	NM_198057	-1.10	14.84	6.69	-8.15	-1.15	3.24
C9orf114	NM_016390	-0.71	6.98	1.64	-5.34	-2.09	3.20
GNPDA2	NM_138335	-0.87	6.75	1.58	-5.17	-2.09	3.16
DDX42	NM_203499	-1.09	16.50	8.51	-7.99	-0.95	3.15
GCLC	NM_001498	-0.99	12.91	5.57	-7.33	-1.21	3.12
VPS4B	NM_004869	-0.33	11.50	4.49	-7.00	-1.36	3.12
SORBS3	NM_005775	-0.31	9.12	2.92	-6.20	-1.64	3.10
DUSP5	NM_004419	-0.28	13.31	6.00	-7.31	-1.15	3.09
NDFIP1	NM_030571	-0.90	15.12	7.60	-7.52	-0.99	3.08
NT5C2	NM_001134373	-0.31	8.29	2.65	-5.64	-1.64	2.99
HSBP1L1	NM_001136180	-0.30	10.34	4.04	-6.30	-1.36	2.98
ITGA2	NM_002203	-0.63	11.52	5.01	-6.50	-1.20	2.96
MKI67IP	NM_032390	-0.27	19.37	12.23	-7.14	-0.66	2.91
PRKAR2A	NM_004157	-0.47	9.79	3.82	-5.96	-1.36	2.91
RBL2	NM_005611	-0.61	10.07	4.35	-5.72	-1.21	2.79
SEL1L3	NM_015187	-0.80	17.64	11.34	-6.30	-0.64	2.73
PPP2R5E	NM_006246	-0.32	10.00	4.51	-5.49	-1.15	2.71
ENDOD1	NM_015036	-0.24	8.28	3.23	-5.04	-1.36	2.70
XPO6	NM_015171	-0.35	14.64	8.92	-5.71	-0.71	2.61

**mir-19a-3p (MIMAT0000073)**							
**Gene Symbol**	**RefSeq ID**	**mirSVR Score**	**SR**	**SEN**	**dRPKM**	**FC**	**D**
IL8	NM_000584	-0.46	89.60	8.90	-80.69	-3.33	7.16
IL33	NM_033439	-0.59	35.40	1.12	-34.28	-4.98	7.13
NGFRAP1	NM_206915	-0.66	34.92	3.15	-31.77	-3.47	6.08
NGFRAP1	NM_014380	-0.65	31.96	3.41	-28.56	-3.23	5.82
UPF3A	NM_023011	-0.29	18.45	1.27	-17.18	-3.86	5.63
BCAR3	NM_003567	-0.22	15.52	0.96	-14.56	-4.02	5.57

NDUFB2	NM_004546	-0.43	68.00	30.66	-37.34	-1.15	5.35
RPS27L	NM_015920	-0.62	78.82	47.61	-31.22	-0.73	5.02
FAM129B	NM_022833	-1.14	87.20	56.11	-31.09	-0.64	5.00
PRKRA	NM_003690	-0.95	15.60	1.66	-13.93	-3.23	4.99
STAMBPL1	NM_020799	-0.54	14.26	1.52	-12.74	-3.23	4.89
EMC7	NM_020154	-0.53	46.72	20.18	-26.54	-1.21	4.88
ACTL6A	NM_004301	-0.52	23.69	4.90	-18.79	-2.27	4.80
RAP1B	NM_001010942	-1.15	55.53	30.38	-25.15	-0.87	4.73
PARP3	NM_001003931	-0.78	12.09	1.29	-10.80	-3.23	4.71
F3	NM_001993	-1.04	11.90	1.27	-10.63	-3.23	4.70
MEDAG	NM_032849	-1.18	53.08	29.21	-23.87	-0.86	4.66
DCBLD2	NM_080927	-0.32	63.32	39.36	-23.96	-0.69	4.63
SLC31A2	NM_001860	-1.22	13.14	1.71	-11.42	-2.94	4.58
CCNC	NM_005190	-0.41	19.08	3.95	-15.13	-2.27	4.53
WDR44	NM_019045	-1.34	8.12	0.73	-7.39	-3.47	4.51
RALA	NM_005402	-0.42	35.93	16.20	-19.73	-1.15	4.45
TPRG1L	NM_182752	-0.55	35.20	16.26	-18.95	-1.11	4.39
POLE4	NM_019896	-0.75	18.60	4.36	-14.24	-2.09	4.37
GNAQ	NM_002072	-0.27	17.69	4.15	-13.54	-2.09	4.30
SLC25A44	NM_014655	-0.39	7.84	0.84	-7.01	-3.23	4.28
FBXO28	NM_015176	-0.23	6.18	0.56	-5.62	-3.47	4.27
PPTC7	NM_139283	-0.70	7.60	0.81	-6.79	-3.23	4.25
TSN	NM_004622	-0.68	17.48	4.45	-13.02	-1.97	4.20
FAM69A	NM_001006605	-0.96	8.97	1.17	-7.80	-2.94	4.17
COX6C	NM_004374	-0.84	25.30	9.89	-15.41	-1.36	4.17
ZBTB4	NM_020899	-0.90	12.64	2.55	-10.08	-2.31	4.05
UFD1L	NM_001035247	-0.69	10.48	1.75	-8.72	-2.58	4.05
RAD23B	NM_002874	-0.90	30.48	15.32	-15.17	-0.99	4.05
UFD1L	NM_005659	-0.71	10.16	1.70	-8.46	-2.58	4.02
PTP4A1	NM_003463	-0.21	20.89	7.77	-13.12	-1.43	3.98
C2orf76	NM_001017927	-0.26	13.63	3.20	-10.43	-2.09	3.98
FZD8	NM_031866	-0.64	7.31	0.95	-6.36	-2.94	3.97
KLHL12	NM_021633	-0.74	6.98	0.91	-6.07	-2.94	3.93
POU4F1	NM_006237	-0.78	11.54	2.39	-9.15	-2.27	3.92
BMPER	NM_133468	-0.73	6.86	0.89	-5.96	-2.94	3.91
GPR137B	NM_003272	-0.95	8.88	1.49	-7.39	-2.58	3.87
PIK3IP1	NM_001135911	-0.63	14.06	3.80	-10.26	-1.89	3.85
RBM26	NM_022118	-0.27	6.45	0.84	-5.61	-2.94	3.85
RPL27A	NM_000990	-0.20	21.76	9.33	-12.43	-1.22	3.83
NRBP1	NM_013392	-0.96	34.15	20.71	-13.44	-0.72	3.82
PIK3IP1	NM_052880	-0.67	13.61	3.68	-9.93	-1.89	3.81
REP15	NM_001029874	-0.30	11.37	2.67	-8.70	-2.09	3.76
BMP6	NM_001718	-1.04	22.51	10.75	-11.76	-1.07	3.71

TMEM167B	NM_020141	-0.55	12.24	3.31	-8.93	-1.89	3.68
SUPV3L1	NM_003171	-0.43	7.33	1.23	-6.10	-2.58	3.67
RAB3B	NM_002867	-0.90	26.41	14.89	-11.52	-0.83	3.62
GNRH1	NM_001083111	-0.28	10.11	2.37	-7.74	-2.09	3.62
VDAC3	NM_005662	-0.20	30.78	19.10	-11.68	-0.69	3.61
GTDC1	NM_001006636	-0.23	6.73	1.13	-5.60	-2.58	3.58
ELK3	NM_005230	-1.08	13.24	4.23	-9.01	-1.64	3.57
NCBP2	NM_007362	-0.61	13.12	4.20	-8.93	-1.64	3.56
NAP1L1	NM_139207	-0.49	23.85	12.96	-10.89	-0.88	3.56
CCNA2	NM_001237	-0.65	6.48	1.08	-5.39	-2.58	3.54
SDC1	NM_002997	-1.15	18.60	8.53	-10.07	-1.12	3.52
PHLDA1	NM_007350	-0.43	18.83	8.73	-10.10	-1.11	3.52
PURA	NM_005859	-1.19	14.18	5.16	-9.02	-1.46	3.49
RAB13	NM_002870	-1.02	29.19	18.43	-10.76	-0.66	3.49
SAP18	NM_005870	-0.51	12.38	3.96	-8.42	-1.64	3.49
PSMD9	NM_002813	-0.31	12.16	3.89	-8.27	-1.64	3.46
MGAT2	NM_002408	-0.39	14.33	5.60	-8.73	-1.36	3.41
BOLA3	NM_212552	-0.37	14.02	5.48	-8.54	-1.36	3.38
TMEM106C	NM_001143842	-0.41	8.15	1.91	-6.24	-2.09	3.37
SNX5	NM_014426	-0.89	16.36	7.38	-8.98	-1.15	3.37
C1orf9	NM_014283	-1.18	9.61	2.68	-6.93	-1.84	3.35
ANTXR2	NM_058172	-0.40	17.71	8.68	-9.03	-1.03	3.34
UBE2D2	NM_003339	-1.01	10.54	3.37	-7.17	-1.64	3.28
SEC14L1	NM_003003	-0.66	7.07	1.66	-5.41	-2.09	3.21
ABR	NM_021962	-0.97	9.37	2.89	-6.48	-1.70	3.18
ATXN10	NM_013236	-1.11	16.33	8.20	-8.12	-0.99	3.18
RAB21	NM_014999	-0.45	18.73	10.40	-8.33	-0.85	3.17
TROVE2	NM_004600	-0.88	11.98	4.68	-7.30	-1.36	3.17
UBE2V1	NM_001032288	-0.43	18.21	9.96	-8.25	-0.87	3.17
NDFIP2	NM_019080	-1.04	11.68	4.56	-7.12	-1.36	3.14
YTHDF2	NM_016258	-1.11	9.31	2.98	-6.33	-1.64	3.13
AKTIP	NM_022476	-0.36	17.71	9.69	-8.02	-0.87	3.13
GCLC	NM_001498	-0.23	12.91	5.57	-7.33	-1.21	3.12
VPS4B	NM_004869	-1.18	11.50	4.49	-7.00	-1.36	3.12
GLRX5	NM_016417	-1.06	15.48	7.78	-7.70	-0.99	3.11
SETD7	NM_030648	-0.31	12.92	5.63	-7.30	-1.20	3.11
NDFIP1	NM_030571	-1.10	15.12	7.60	-7.52	-0.99	3.08
SPRY2	NM_005842	-0.27	11.04	4.32	-6.73	-1.36	3.07
UBA3	NM_003968	-1.26	20.77	12.89	-7.88	-0.69	3.06
TIPARP	NM_015508	-1.06	12.20	5.27	-6.93	-1.21	3.04
INSIG1	NM_198337	-1.01	12.38	5.58	-6.80	-1.15	2.99
ATMIN	NM_015251	-1.02	9.05	3.12	-5.93	-1.54	2.99
HNRNPF	NM_001098206	-1.10	22.48	14.90	-7.58	-0.59	2.98

ZFP91	NM_053023	-0.65	11.31	4.77	-6.53	-1.24	2.98
ARL6IP1	NM_015161	-0.76	10.29	4.02	-6.27	-1.36	2.98
ITGA2	NM_002203	-0.84	11.52	5.01	-6.50	-1.20	2.96
ACBD5	NM_145698	-1.14	9.75	3.81	-5.94	-1.36	2.91
NAP1L1	NM_004537	-0.36	20.71	13.62	-7.09	-0.60	2.89
INSIG1	NM_005542	-1.02	11.18	5.04	-6.14	-1.15	2.86
FMR1	NM_002024	-1.10	12.33	6.19	-6.13	-0.99	2.80
UTRN	NM_007124	-0.22	11.04	5.13	-5.91	-1.11	2.79
PIP4K2B	NM_003559	-0.30	7.68	2.65	-5.03	-1.54	2.79
SECISBP2L	NM_014701	-0.70	11.40	5.61	-5.80	-1.02	2.73
TMEM189-UBE2V1	NM_199203	-0.43	13.31	7.28	-6.03	-0.87	2.73
SEL1L3	NM_015187	-0.81	17.64	11.34	-6.30	-0.64	2.73
PPP2R5E	NM_006246	-1.23	10.00	4.51	-5.49	-1.15	2.71
IER5L	NM_203434	-0.24	16.24	10.08	-6.16	-0.69	2.71
CS	NM_004077	-0.76	13.05	7.14	-5.91	-0.87	2.71
DYNC1LI2	NM_006141	-0.93	16.12	10.50	-5.62	-0.62	2.57
NECAP1	NM_015509	-0.31	10.98	5.85	-5.13	-0.91	2.53
RTCD1	NM_001130841	-0.29	10.85	5.78	-5.07	-0.91	2.51
RUFY1	NM_025158	-0.52	10.85	5.78	-5.07	-0.91	2.51
DNM1L	NM_012062	-0.27	15.16	9.87	-5.28	-0.62	2.48
TNIP1	NM_006058	-0.48	13.47	8.36	-5.11	-0.69	2.45

**mir-20a-5p (MIMAT0000075)**							
**Gene Symbol**	**RefSeq ID**	**mirSVR Score**	**SR**	**SEN**	**dRPKM**	**FC**	**D**
TFPI2	NM_006528	-1.23	528.90	225.72	-303.17	-1.23	8.34
IL8	NM_000584	-1.18	89.60	8.90	-80.69	-3.33	7.16
INHBA	NM_002192	-0.36	282.13	177.25	-104.88	-0.67	6.75
NGFRAP1	NM_014380	-0.35	31.96	3.41	-28.56	-3.23	5.82
UPF3A	NM_023011	-0.31	18.45	1.27	-17.18	-3.86	5.63
CD36	NM_001127443	-0.28	47.21	11.74	-35.47	-2.01	5.53
MMP1	NM_002421	-0.31	69.30	31.02	-38.28	-1.16	5.38
RPA2	NM_002946	-0.63	16.56	1.76	-14.79	-3.23	5.05
GMPS	NM_003875	-0.29	13.70	1.24	-12.47	-3.47	5.03
STC1	NM_003155	-1.12	39.40	13.31	-26.09	-1.57	4.96
ACTL6A	NM_004301	-0.29	23.69	4.90	-18.79	-2.27	4.80
DCTN3	NM_024348	-0.48	19.61	3.29	-16.33	-2.58	4.78
LANCL1	NM_001136574	-0.63	8.61	0.67	-7.93	-3.68	4.74
PARP3	NM_001003931	-0.43	12.09	1.29	-10.80	-3.23	4.71
F3	NM_001993	-1.18	11.90	1.27	-10.63	-3.23	4.70
DCBLD2	NM_080927	-0.58	63.32	39.36	-23.96	-0.69	4.63
TPRG1L	NM_182752	-1.02	35.20	16.26	-18.95	-1.11	4.39

NDUFA4	NM_002489	-1.00	41.52	22.13	-19.39	-0.91	4.37
ADI1	NM_018269	-0.42	20.08	5.43	-14.65	-1.89	4.31
FBXO28	NM_001136115	-0.50	6.41	0.58	-5.83	-3.47	4.30
URGCP	NM_001077663	-0.31	7.87	0.84	-7.03	-3.23	4.28
SLC25A44	NM_014655	-0.23	7.84	0.84	-7.01	-3.23	4.28
FBXO28	NM_015176	-0.58	6.18	0.56	-5.62	-3.47	4.27
UBA6	NM_018227	-0.35	15.66	3.30	-12.37	-2.25	4.27
MAF1	NM_032272	-0.36	18.96	5.13	-13.83	-1.89	4.23
SQSTM1	NM_003900	-0.98	27.66	11.51	-16.15	-1.27	4.21
M6PR	NM_002355	-0.85	9.08	1.18	-7.90	-2.94	4.19
SAR1B	NM_016103	-1.28	14.67	3.25	-11.41	-2.17	4.13
ZBTB4	NM_020899	-1.05	12.64	2.55	-10.08	-2.31	4.05
UFD1L	NM_001035247	-0.98	10.48	1.75	-8.72	-2.58	4.05
SLC35A5	NM_017945	-0.75	7.93	1.03	-6.89	-2.94	4.05
RAD23B	NM_002874	-0.76	30.48	15.32	-15.17	-0.99	4.05
MITD1	NM_138798	-0.20	14.23	3.34	-10.89	-2.09	4.03
UFD1L	NM_005659	-0.99	10.16	1.70	-8.46	-2.58	4.02
PTP4A1	NM_003463	-0.46	20.89	7.77	-13.12	-1.43	3.98
RRM2	NM_001034	-0.73	7.13	0.93	-6.21	-2.94	3.95
SERPINB8	NM_002640	-0.52	6.91	0.90	-6.01	-2.94	3.92
SLC16A7	NM_004731	-0.51	6.58	0.86	-5.72	-2.94	3.87
GPR137B	NM_003272	-0.76	8.88	1.49	-7.39	-2.58	3.87
ERRFI1	NM_018948	-0.41	12.41	2.91	-9.50	-2.09	3.86
MKRN1	NM_013446	-1.08	12.33	2.89	-9.44	-2.09	3.86
NRBP1	NM_013392	-0.76	34.15	20.71	-13.44	-0.72	3.82
PFKP	NM_002627	-1.29	30.21	17.14	-13.07	-0.82	3.80
CYTSB	NM_001033553	-0.65	5.90	0.77	-5.13	-2.94	3.77
LSM5	NM_012322	-0.42	8.06	1.35	-6.71	-2.58	3.77
LYRM5	NM_001001660	-0.87	11.41	2.67	-8.73	-2.09	3.76
GTDC1	NM_001006636	-1.08	6.73	1.13	-5.60	-2.58	3.58
SLC39A6	NM_012319	-0.94	23.60	12.58	-11.02	-0.91	3.58
ELK3	NM_005230	-1.22	13.24	4.23	-9.01	-1.64	3.57
MYO10	NM_012334	-0.28	16.54	6.64	-9.90	-1.32	3.56
PURA	NM_005859	-1.02	14.18	5.16	-9.02	-1.46	3.49
TXNDC12	NM_015913	-0.64	14.57	5.70	-8.88	-1.36	3.43
MRPL24	NM_145729	-1.24	19.55	9.82	-9.73	-0.99	3.43
MGAT2	NM_002408	-0.21	14.33	5.60	-8.73	-1.36	3.41
ACIN1	NM_014977	-0.21	14.16	5.53	-8.63	-1.36	3.39
C4orf33	NM_001099783	-0.81	8.15	1.91	-6.24	-2.09	3.37
TXNIP	NM_006472	-1.27	27.42	17.63	-9.79	-0.64	3.35
COMMD10	NM_016144	-0.30	20.00	10.66	-9.34	-0.91	3.35
C1orf9	NM_014283	-1.06	9.61	2.68	-6.93	-1.84	3.35
CCNDBP1	NM_012142	-0.34	9.22	2.49	-6.73	-1.89	3.33

UBE2B	NM_003337	-0.93	10.83	3.46	-7.36	-1.64	3.32
NTN4	NM_021229	-1.21	23.64	14.28	-9.36	-0.73	3.31
RSU1	NM_012425	-0.21	15.87	7.28	-8.59	-1.12	3.30
CENPQ	NM_018132	-1.27	7.49	1.76	-5.74	-2.09	3.28
COPG2	NM_012133	-0.46	7.47	1.75	-5.72	-2.09	3.27
SLC35F5	NM_025181	-1.02	22.73	13.86	-8.87	-0.71	3.23
ABR	NM_021962	-0.34	9.37	2.89	-6.48	-1.70	3.18
RAB21	NM_014999	-0.43	18.73	10.40	-8.33	-0.85	3.17
TBP	NM_003194	-0.24	6.80	1.59	-5.20	-2.09	3.17
GNPDA2	NM_138335	-1.25	6.75	1.58	-5.17	-2.09	3.16
CDCA4	NM_145701	-0.22	6.72	1.58	-5.14	-2.09	3.16
ABTB1	NM_172027	-0.27	11.79	4.61	-7.18	-1.36	3.15
HMGN4	NM_006353	-0.78	6.58	1.54	-5.04	-2.09	3.13
AKTIP	NM_022476	-0.47	17.71	9.69	-8.02	-0.87	3.13
YIPF2	NM_024029	-0.25	22.38	14.13	-8.25	-0.66	3.12
MAGT1	NM_032121	-0.29	16.10	8.30	-7.79	-0.95	3.11
GOLGB1	NM_004487	-0.34	12.27	5.16	-7.11	-1.25	3.09
SFRS4	NM_005626	-0.24	13.30	6.00	-7.30	-1.15	3.09
DCAF16	NM_017741	-0.85	7.40	2.00	-5.40	-1.89	3.08
TIPARP	NM_015508	-0.40	12.20	5.27	-6.93	-1.21	3.04
CEP120	NM_153223	-1.19	7.14	1.93	-5.21	-1.89	3.04
CHD9	NM_025134	-1.00	7.91	2.38	-5.52	-1.73	3.01
KDSR	NM_002035	-0.29	10.46	4.09	-6.37	-1.36	3.00
ATMIN	NM_015251	-0.60	9.05	3.12	-5.93	-1.54	2.99
ZFP91	NM_053023	-0.61	11.31	4.77	-6.53	-1.24	2.98
LIN7B	NM_022165	-1.10	10.29	4.02	-6.27	-1.36	2.97
UBE3C	NM_014671	-0.54	14.49	7.62	-6.88	-0.93	2.93
ACBD5	NM_145698	-0.71	9.75	3.81	-5.94	-1.36	2.91
NAP1L1	NM_004537	-0.24	20.71	13.62	-7.09	-0.60	2.89
NBL1	NM_182744	-0.36	13.82	7.36	-6.45	-0.91	2.84
FGL2	NM_006682	-0.77	18.81	12.09	-6.71	-0.64	2.82
RAB11FIP5	NM_015470	-1.13	12.52	6.29	-6.23	-0.99	2.82
VPS26A	NM_001035260	-0.69	9.11	3.56	-5.55	-1.36	2.82
MAPKSP1	NM_021970	-0.30	9.07	3.55	-5.53	-1.36	2.81
RBL2	NM_005611	-1.07	10.07	4.35	-5.72	-1.21	2.79
PDZD11	NM_016484	-1.15	18.70	12.18	-6.52	-0.62	2.77
SSFA2	NM_006751	-0.80	12.51	6.45	-6.06	-0.95	2.77
VPS26A	NM_004896	-0.70	8.63	3.37	-5.26	-1.36	2.75
SSFA2	NM_001130445	-0.77	12.23	6.31	-5.92	-0.95	2.74
CRK	NM_005206	-0.91	12.79	6.82	-5.97	-0.91	2.73
DPM2	NM_003863	-0.26	11.72	5.89	-5.83	-0.99	2.73
PPP2R5E	NM_006246	-1.19	10.00	4.51	-5.49	-1.15	2.71
ARL1	NM_001177	-0.90	18.55	12.29	-6.26	-0.59	2.71

ATP2B1	NM_001682	-0.52	10.31	4.93	-5.39	-1.07	2.65
CRK	NM_016823	-0.93	11.88	6.33	-5.55	-0.91	2.63
ATP2B1	NM_001001323	-0.46	10.08	4.82	-5.27	-1.07	2.62
MGLL	NM_001003794	-0.24	16.69	10.87	-5.82	-0.62	2.61
DYNC1LI2	NM_006141	-1.14	16.12	10.50	-5.62	-0.62	2.57
RHOT1	NM_001033568	-0.34	11.74	6.42	-5.32	-0.87	2.56
RTCD1	NM_001130841	-0.62	10.85	5.78	-5.07	-0.91	2.51
DNM1L	NM_012062	-0.50	15.16	9.87	-5.28	-0.62	2.48

mir-100-5p (MIMAT0000098)							
**Gene Symbol**	**RefSeq ID**	**mirSVR Score**	**SR**	**SEN**	**dRPKM**	**FC**	**D**
RAP1B	NM_001010942	-1.14	55.53	30.38	-25.15	-0.87	4.73
FZD8	NM_031866	-1.14	7.31	0.95	-6.36	-2.94	3.97
EPDR1	NM_017549	-0.37	30.89	17.53	-13.37	-0.82	3.83
PL-5283	NM_001130929	-0.39	13.47	3.65	-9.83	-1.89	3.80
SIAH2	NM_005067	-1.15	6.92	1.16	-5.76	-2.58	3.61
TSC22D3	NM_198057	-0.60	14.84	6.69	-8.15	-1.15	3.24
C9orf123	NM_033428	-0.92	9.37	3.66	-5.71	-1.36	2.86

mir-125b-5p (MIMAT0000423)							
**Gene Symbol**	**RefSeq ID**	**mirSVR Score**	**SR**	**SEN**	**dRPKM**	**FC**	**D**
RPA2	NM_002946	-0.41	16.56	1.76	-14.79	-3.23	5.05
PRKRA	NM_003690	-0.22	15.60	1.66	-13.93	-3.23	4.99
STC1	NM_003155	-0.56	39.40	13.31	-26.09	-1.57	4.96
ACTL6A	NM_004301	-0.28	23.69	4.90	-18.79	-2.27	4.80
SNRPB	NM_003091	-0.62	29.19	7.90	-21.29	-1.89	4.80
TMEM50A	NM_014313	-0.44	43.86	19.78	-24.09	-1.15	4.73
PLIN3	NM_005817	-0.32	38.73	16.86	-21.86	-1.20	4.61
MLF2	NM_005439	-0.34	24.20	6.55	-17.65	-1.89	4.55
CCNC	NM_005190	-0.42	19.08	3.95	-15.13	-2.27	4.53
RABL6	NM_024718	-0.36	9.10	0.97	-8.13	-3.23	4.42
DSTN	NM_006870	-0.22	56.15	35.73	-20.41	-0.65	4.40
ANPEP	NM_001150	-0.32	58.88	39.09	-19.79	-0.59	4.35
ESRRA	NM_004451	-0.93	10.49	1.37	-9.13	-2.94	4.34
SLC35A5	NM_017945	-0.24	7.93	1.03	-6.89	-2.94	4.05
TIMM17B	NM_005834	-0.28	13.63	3.20	-10.43	-2.09	3.98
RRM2	NM_001034	-0.35	7.13	0.93	-6.21	-2.94	3.95
TRIB1	NM_025195	-0.30	6.42	0.84	-5.58	-2.94	3.85

HAX1	NM_006118	-0.25	19.83	7.75	-12.08	-1.36	3.84
TCTA	NM_022171	-0.26	8.44	1.41	-7.03	-2.58	3.82
ZNF828	NM_001164145	-0.96	6.17	0.80	-5.36	-2.94	3.81
KIAA0174	NM_014761	-0.65	20.94	9.05	-11.90	-1.21	3.77
OSBPL9	NM_148909	-1.22	9.94	2.33	-7.61	-2.09	3.60
NT5C3L	NM_052935	-0.45	16.39	6.40	-9.98	-1.36	3.59
TBC1D1	NM_015173	-0.40	10.47	2.67	-7.80	-1.97	3.56
PSMD9	NM_002813	-0.95	12.16	3.89	-8.27	-1.64	3.46
TXNIP	NM_006472	-0.45	27.42	17.63	-9.79	-0.64	3.35
PRRC1	NM_130809	-0.96	10.49	3.24	-7.25	-1.70	3.32
FIBP	NM_198897	-0.83	19.64	10.47	-9.17	-0.91	3.32
MRPL10	NM_145255	-0.21	7.40	1.74	-5.67	-2.09	3.26
TSC22D3	NM_198057	-0.71	14.84	6.69	-8.15	-1.15	3.24
MED15	NM_015889	-0.22	11.93	4.66	-7.27	-1.36	3.17
ABTB1	NM_172027	-0.30	11.79	4.61	-7.18	-1.36	3.15
DDX42	NM_203499	-0.48	16.50	8.51	-7.99	-0.95	3.15
ZSWIM6	NM_020928	-1.26	8.94	2.76	-6.18	-1.70	3.13
VPS4B	NM_004869	-1.11	11.50	4.49	-7.00	-1.36	3.12
GOLGB1	NM_004487	-0.23	12.27	5.16	-7.11	-1.25	3.09
OAZ2	NM_002537	-0.40	14.86	7.92	-6.94	-0.91	2.94
PSMG3	NM_001134340	-0.22	9.97	3.90	-6.07	-1.36	2.93
TAF9B	NM_015975	-1.04	8.67	3.39	-5.28	-1.36	2.76
HAS1	NM_001523	-0.35	16.13	10.18	-5.95	-0.66	2.66
MRPS10	NM_018141	-0.21	15.73	9.93	-5.80	-0.66	2.62
SLC39A9	NM_018375	-0.84	13.90	8.43	-5.47	-0.72	2.56
RTCD1	NM_001130841	-0.43	10.85	5.78	-5.07	-0.91	2.51

mir-92a-1-5p (MIMAT0004507)							
**Gene Symbol**	**RefSeq ID**	**mirSVR Score**	**SR**	**SEN**	**dRPKM**	**FC**	**D**
H2AFZ	NM_002106	-0.30	68.35	35.26	-33.09	-0.95	5.14
RAP1B	NM_001010942	-0.22	55.53	30.38	-25.15	-0.87	4.73
TMEM50A	NM_014313	-1.03	43.86	19.78	-24.09	-1.15	4.73
ATPIF1	NM_178191	-0.24	25.56	7.88	-17.67	-1.70	4.48
MEA1	NM_014623	-0.51	44.85	24.54	-20.31	-0.87	4.43
ZFAND3	NM_021943	-0.20	8.55	0.91	-7.63	-3.23	4.36
EGLN2	NM_053046	-0.37	10.38	1.35	-9.03	-2.94	4.33
LOC729991	NM_001145784	-0.46	12.65	2.12	-10.53	-2.58	4.26
NR3C1	NM_001020825	-0.64	26.80	10.96	-15.84	-1.29	4.19
PA2G4	NM_006191	-0.81	22.68	8.09	-14.59	-1.49	4.14
MRPS18B	NM_014046	-0.25	18.57	5.94	-12.63	-1.64	4.01
PTP4A1	NM_003463	-0.51	20.89	7.77	-13.12	-1.43	3.98

SERPINB8	NM_002640	-0.49	6.91	0.90	-6.01	-2.94	3.92
TCTA	NM_022171	-0.21	8.44	1.41	-7.03	-2.58	3.82
CYTSB	NM_001033553	-0.21	5.90	0.77	-5.13	-2.94	3.77
TPP1	NM_000391	-0.55	10.99	2.58	-8.42	-2.09	3.72
HIST1H2BM	NM_003521	-0.29	17.42	6.81	-10.61	-1.36	3.67
IP6K2	NM_001005911	-0.59	10.42	2.44	-7.98	-2.09	3.65
ABCF1	NM_001025091	-0.27	18.69	7.89	-10.80	-1.24	3.65
SIAH2	NM_005067	-0.63	6.92	1.16	-5.76	-2.58	3.61
OSBPL9	NM_148909	-0.54	9.94	2.33	-7.61	-2.09	3.60
IP6K2	NM_001146179	-0.56	9.89	2.32	-7.57	-2.09	3.59
C17orf49	NM_001142798	-0.24	20.07	10.08	-9.99	-0.99	3.47
AIDA	NM_022831	-0.21	24.98	15.15	-9.83	-0.72	3.38
DPT	NM_001937	-1.04	7.49	1.76	-5.74	-2.09	3.28
CCDC92	NM_025140	-0.33	7.22	1.69	-5.53	-2.09	3.23
NOL10	NM_024894	-0.59	14.08	6.08	-8.00	-1.21	3.23
MED15	NM_015889	-0.24	11.93	4.66	-7.27	-1.36	3.17
C20orf132	NM_213632	-0.24	11.75	4.59	-7.16	-1.36	3.15
AKTIP	NM_022476	-0.25	17.71	9.69	-8.02	-0.87	3.13
TPBG	NM_006670	-0.21	12.98	6.27	-6.71	-1.05	2.94
SRR	NM_021947	-0.95	9.41	3.68	-5.73	-1.36	2.86
POGK	NM_017542	-0.87	7.39	2.36	-5.03	-1.64	2.85
DDX24	NM_020414	-0.26	20.20	13.39	-6.81	-0.59	2.83
DPM2	NM_003863	-0.32	11.72	5.89	-5.83	-0.99	2.73

**let-7a-2-3p (MIMAT0010195)**							
**Gene Symbol**	**RefSeq ID**	**mirSVR Score**	**SR**	**SEN**	**dRPKM**	**FC**	**D**
IL8	NM_000584	-0.31	89.60	8.90	-80.69	-3.33	7.16
IL33	NM_033439	-1.22	35.40	1.12	-34.28	-4.98	7.13
RPS25	NM_001028	-1.27	268.93	142.20	-126.74	-0.92	7.05
SFRS13A	NM_054016	-0.29	14.40	0.99	-13.41	-3.86	5.38
NDUFB2	NM_004546	-0.37	68.00	30.66	-37.34	-1.15	5.35
H2AFZ	NM_002106	-0.26	68.35	35.26	-33.09	-0.95	5.14
RPA2	NM_002946	-0.24	16.56	1.76	-14.79	-3.23	5.05
SNRPB	NM_198216	-0.67	33.43	9.04	-24.38	-1.89	4.98
STC1	NM_003155	-1.19	39.40	13.31	-26.09	-1.57	4.96
FGFR1OP	NM_007045	-1.26	15.11	1.61	-13.50	-3.23	4.95
SRGN	NM_002727	-0.72	64.01	36.31	-27.70	-0.82	4.86
NME1	NM_198175	-0.30	52.74	26.50	-26.24	-0.99	4.82
SNRPB	NM_003091	-0.82	29.19	7.90	-21.29	-1.89	4.80
RAP1B	NM_001010942	-0.48	55.53	30.38	-25.15	-0.87	4.73
SERPINE2	NM_006216	-0.41	69.92	44.34	-25.57	-0.66	4.72

PARP3	NM_001003931	-0.66	12.09	1.29	-10.80	-3.23	4.71
F3	NM_001993	-0.29	11.90	1.27	-10.63	-3.23	4.70
ELOVL1	NM_022821	-0.40	36.66	14.33	-22.34	-1.36	4.68
TNFRSF10D	NM_003840	-0.26	57.91	33.52	-24.39	-0.79	4.68
ANKRD1	NM_014391	-0.21	30.17	10.76	-19.40	-1.49	4.53
BASP1	NM_006317	-0.59	50.43	28.72	-21.71	-0.81	4.51
WDR44	NM_019045	-0.40	8.12	0.73	-7.39	-3.47	4.51
TXNDC11	NM_015914	-0.23	9.35	1.00	-8.35	-3.23	4.45
MEA1	NM_014623	-1.13	44.85	24.54	-20.31	-0.87	4.43
ESRRA	NM_004451	-0.69	10.49	1.37	-9.13	-2.94	4.34
ADI1	NM_018269	-0.52	20.08	5.43	-14.65	-1.89	4.31
UBA6	NM_018227	-0.23	15.66	3.30	-12.37	-2.25	4.27
GOSR2	NM_054022	-0.48	12.34	2.07	-10.27	-2.58	4.24
CCDC51	NM_024661	-0.25	12.03	2.02	-10.02	-2.58	4.21
SAR1B	NM_016103	-0.38	14.67	3.25	-11.41	-2.17	4.13
COPS7A	NM_016319	-0.58	26.65	11.51	-15.14	-1.21	4.10
KCTD9	NM_017634	-0.27	26.65	11.60	-15.04	-1.20	4.09
RAD23B	NM_002874	-0.66	30.48	15.32	-15.17	-0.99	4.05
GNG5	NM_005274	-0.99	41.76	26.36	-15.40	-0.66	4.00
PTP4A1	NM_003463	-0.82	20.89	7.77	-13.12	-1.43	3.98
FZD8	NM_031866	-0.59	7.31	0.95	-6.36	-2.94	3.97
SERPINB8	NM_002640	-0.92	6.91	0.90	-6.01	-2.94	3.92
PDGFC	NM_016205	-0.41	12.91	3.03	-9.88	-2.09	3.91
GPR137B	NM_003272	-0.43	8.88	1.49	-7.39	-2.58	3.87
TRIB1	NM_025195	-0.38	6.42	0.84	-5.58	-2.94	3.85
PTPLAD1	NM_016395	-0.32	15.33	4.73	-10.60	-1.70	3.81
KIAA0174	NM_014761	-0.54	20.94	9.05	-11.90	-1.21	3.77
HMGN1	NM_004965	-0.24	33.75	20.95	-12.80	-0.69	3.74
BMP6	NM_001718	-0.26	22.51	10.75	-11.76	-1.07	3.71
DHX36	NM_020865	-0.64	10.76	2.52	-8.23	-2.09	3.69
TPM3	NM_001043352	-0.57	24.53	12.71	-11.82	-0.95	3.69
BAG3	NM_004281	-1.28	7.05	1.18	-5.87	-2.58	3.63
COIL	NM_004645	-1.02	6.88	1.15	-5.73	-2.58	3.60
WSB1	NM_015626	-0.94	22.86	11.79	-11.07	-0.95	3.60
DENR	NM_003677	-0.85	19.45	8.93	-10.53	-1.12	3.58
TBC1D1	NM_015173	-0.58	10.47	2.67	-7.80	-1.97	3.56
NAP1L1	NM_139207	-0.52	23.85	12.96	-10.89	-0.88	3.56
PAPSS2	NM_001015880	-0.94	20.80	10.23	-10.58	-1.02	3.55
SDC1	NM_002997	-0.48	18.60	8.53	-10.07	-1.12	3.52
PURA	NM_005859	-0.22	14.18	5.16	-9.02	-1.46	3.49
MPPE1	NM_023075	-0.80	6.13	1.03	-5.10	-2.58	3.49
AP3S1	NM_001284	-0.31	22.10	11.77	-10.32	-0.91	3.49
DCTN2	NM_006400	-1.31	22.62	12.38	-10.24	-0.87	3.47

SEC62	NM_003262	-0.46	16.23	6.96	-9.27	-1.22	3.44
SFRS13A	NM_006625	-0.29	17.03	7.68	-9.35	-1.15	3.42
DCAF12	NM_015397	-0.59	9.17	2.48	-6.69	-1.89	3.33
GM2A	NM_000405	-0.37	9.13	2.47	-6.66	-1.89	3.32
UBE2B	NM_003337	-0.41	10.83	3.46	-7.36	-1.64	3.32
FAM24A	NM_001029888	-0.23	13.03	5.09	-7.94	-1.36	3.28
PSMA5	NM_002790	-1.18	8.80	2.38	-6.42	-1.89	3.28
DEPDC7	NM_001077242	-0.23	7.38	1.73	-5.65	-2.09	3.26
RNF152	NM_173557	-1.23	7.17	1.68	-5.49	-2.09	3.23
RHBDL2	NM_017821	-1.28	7.05	1.65	-5.39	-2.09	3.21
ANP32B	NM_006401	-1.34	17.62	9.39	-8.23	-0.91	3.17
TROVE2	NM_004600	-0.92	11.98	4.68	-7.30	-1.36	3.17
TBP	NM_003194	-1.24	6.80	1.59	-5.20	-2.09	3.17
RPF1	NM_025065	-1.25	11.80	4.61	-7.19	-1.36	3.15
METTL3	NM_019852	-0.27	6.62	1.55	-5.07	-2.09	3.14
YTHDF2	NM_016258	-0.27	9.31	2.98	-6.33	-1.64	3.13
VPS4B	NM_004869	-0.77	11.50	4.49	-7.00	-1.36	3.12
SNX2	NM_003100	-1.21	11.27	4.40	-6.86	-1.36	3.09
SFRS4	NM_005626	-1.17	13.30	6.00	-7.30	-1.15	3.09
KLHL7	NM_001031710	-0.94	8.97	2.87	-6.10	-1.64	3.08
SPRY2	NM_005842	-0.31	11.04	4.32	-6.73	-1.36	3.07
INSIG1	NM_198337	-0.75	12.38	5.58	-6.80	-1.15	2.99
CSTF3	NM_001033506	-1.28	10.37	4.05	-6.32	-1.36	2.98
LIN7B	NM_022165	-0.61	10.29	4.02	-6.27	-1.36	2.97
STXBP1	NM_001032221	-0.24	10.11	3.95	-6.16	-1.36	2.95
MFSD1	NM_022736	-1.12	9.87	3.86	-6.01	-1.36	2.92
STXBP1	NM_003165	-0.22	9.79	3.83	-5.96	-1.36	2.91
PRKAR2A	NM_004157	-1.19	9.79	3.82	-5.96	-1.36	2.91
NAP1L1	NM_004537	-0.39	20.71	13.62	-7.09	-0.60	2.89
C1QBP	NM_001212	-1.34	20.04	13.05	-6.99	-0.62	2.87
SLC33A1	NM_004733	-0.22	7.48	2.39	-5.09	-1.64	2.87
INSIG1	NM_005542	-0.76	11.18	5.04	-6.14	-1.15	2.86
LZIC	NM_032368	-0.42	12.88	6.47	-6.41	-0.99	2.86
ANKRD17	NM_032217	-0.50	9.11	3.56	-5.55	-1.36	2.82
FMR1	NM_002024	-1.08	12.33	6.19	-6.13	-0.99	2.80
ATP2B1	NM_001682	-0.24	10.31	4.93	-5.39	-1.07	2.65
ATP2B1	NM_001001323	-0.20	10.08	4.82	-5.27	-1.07	2.62
FAHD1	NM_031208	-0.67	10.76	5.41	-5.35	-0.99	2.62
DYNC1LI2	NM_006141	-0.53	16.12	10.50	-5.62	-0.62	2.57
RHOT1	NM_001033568	-0.21	11.74	6.42	-5.32	-0.87	2.56
