# Supplementary material for: Opposing activities of oncogenic MIR17HG and tumor suppressive MIR100HG clusters and their gene targets regulate replicative senescence in human adult stem cells
Source: NPJ Aging Mech Dis. 2017 Apr 20;3:7. doi: 10.1038/s41514-017-0006-y (PMC5460214; doi:10.1038/s41514-017-0006-y)
Supplement: Supplementary file 13 — Supplementary Table2 [file 41514_2017_6_MOESM13_ESM.pdf]

Table S2 Protein Targets Down-regulated in SEN

mir-17-5p (MIMAT0000070)						
**Gene Symbol**	**RefSeq ID**	**mirSVR Score**	**SR**	**SEN**	**Diff Mean**	***P*-value**
FPR1	NP_002020	-0.21	9.26E-06	2.59E-06	-6.67E-06	7.93E-05
SH3GLB1	NP_057093	-0.44	1.92E-05	6.38E-06	-1.28E-05	1.18E-04
DNALI1	NP_003453	-0.20	3.16E-05	1.51E-05	-1.65E-05	1.61E-04
RCN2	NP_002893	-0.21	1.11E-05	6.78E-06	-4.30E-06	3.58E-04
IGFBP7	NP_001544	-0.23	1.20E-05	4.12E-06	-7.89E-06	3.70E-04
CA10	NP_001076002	-0.67	2.84E-05	7.68E-06	-2.08E-05	4.35E-04
ZEB2	NP_055610	-0.54	3.21E-05	8.87E-06	-2.32E-05	5.33E-04
KIAA1598	NP_060800	-1.25	5.37E-05	3.34E-05	-2.03E-05	5.48E-04
NUP205	NP_055950	-1.28	2.54E-05	1.12E-05	-1.43E-05	5.66E-04
MAP2	NP_002365	-0.33	8.61E-05	3.58E-05	-5.03E-05	5.76E-04
FGD5	NP_689749	-1.18	1.52E-05	9.77E-06	-5.40E-06	6.26E-04
COL15A1	NP_001846	-0.83	3.44E-05	1.26E-05	-2.18E-05	7.09E-04
AHNAK	NP_001611	-1.29	2.98E-03	2.02E-03	-9.63E-04	7.28E-04
U2AF1	NP_001020374	-0.24	7.00E-05	3.10E-05	-3.90E-05	9.26E-04
TCTEX1D1	NP_689878	-0.89	1.36E-05	5.81E-06	-7.81E-06	1.10E-03
ESR1	NP_000116	-0.40	3.86E-05	1.47E-05	-2.39E-05	1.11E-03
KTN1	NP_001072989	-0.32	2.25E-04	1.51E-04	-7.40E-05	1.18E-03
ANTXR1	NP_444262	-0.48	1.15E-05	5.19E-06	-6.26E-06	1.19E-03
TAOK2	NP_057235	-1.12	1.14E-04	7.57E-05	-3.85E-05	1.25E-03
VASP	NP_003361	-0.22	1.75E-05	8.17E-06	-9.37E-06	1.26E-03
ATXN2	NP_002964	-1.08	1.17E-05	5.04E-06	-6.65E-06	1.31E-03
SIN3A	NP_001138829	-0.54	1.10E-04	8.09E-05	-2.93E-05	1.35E-03
CHD2	NP_001262	-0.78	4.19E-05	1.49E-05	-2.70E-05	1.49E-03
RIT1	NP_008843	-0.50	1.67E-05	7.30E-06	-9.41E-06	1.52E-03
RB1	NP_000312	-0.72	3.99E-04	2.18E-04	-1.81E-04	1.56E-03
HN1	NP_057269	-1.29	1.86E-05	9.90E-06	-8.73E-06	1.64E-03
SUV39H2	NP_001180353	-0.48	4.11E-04	2.37E-04	-1.74E-04	1.80E-03
CHD4	NP_001264	-0.73	9.12E-06	2.78E-06	-6.34E-06	1.97E-03
ARID5B	NP_115575	-0.30	2.46E-05	1.97E-05	-4.90E-06	2.14E-03
DYNC1LI2	NP_006132	-1.14	2.42E-05	1.46E-05	-9.64E-06	2.14E-03
FTL	NP_000137	-0.64	8.54E-06	3.95E-06	-4.59E-06	2.24E-03
TGM2	NP_004604	-1.09	2.95E-04	2.21E-04	-7.42E-05	2.46E-03
ETF1	NP_004721	-0.31	3.52E-05	2.30E-05	-1.22E-05	2.47E-03
CCT5	NP_036205	-0.44	3.76E-05	2.72E-05	-1.04E-05	2.60E-03
NEB	NP_001157980	-0.23	4.45E-04	2.92E-04	-1.53E-04	2.63E-03
ARID1B	NP_059989	-0.40	2.15E-04	1.20E-04	-9.52E-05	2.93E-03
DSTYK	NP_955749	-0.23	1.00E-04	4.89E-05	-5.15E-05	3.28E-03
ANKRD11	NP_037407	-0.23	4.89E-05	3.12E-05	-1.78E-05	3.56E-03

DDX43	NP_061135	-0.39	5.40E-05	3.45E-05	-1.95E-05	3.62E-03
TRIM22	NP_006065	-0.99	8.68E-06	3.86E-06	-4.82E-06	3.69E-03
CHD9	NP_079410	-1.00	4.29E-04	9.94E-05	-3.30E-04	3.92E-03
PDE8A	NP_002596	-0.24	8.89E-05	6.25E-05	-2.64E-05	4.05E-03
RBBP6	NP_008841	-0.46	2.61E-05	1.61E-05	-1.00E-05	4.07E-03
GPD2	NP_001076581	-0.29	7.59E-05	2.22E-05	-5.37E-05	4.20E-03
ZHX2	NP_055758	-0.75	5.23E-05	3.02E-05	-2.21E-05	4.43E-03
CTSA	NP_001121167	-0.94	4.21E-05	3.13E-05	-1.08E-05	4.60E-03
CCDC88A	NP_001129069	-0.83	9.08E-05	6.46E-05	-2.62E-05	4.77E-03
TIAM1	NP_003244	-1.25	6.62E-05	3.46E-05	-3.16E-05	4.82E-03
TOPORS	NP_005793	-1.08	2.14E-04	1.49E-04	-6.42E-05	4.91E-03
ACCS	NP_115981	-0.47	1.63E-05	9.45E-06	-6.80E-06	4.91E-03
NPM1	NP_002511	-1.06	5.48E-05	2.47E-05	-3.01E-05	5.35E-03
JAK3	NP_000206	-0.65	2.73E-05	1.11E-05	-1.62E-05	5.41E-03
MSR1	NP_619729	-1.11	3.63E-05	2.64E-05	-9.85E-06	5.53E-03
RPS6KA5	NP_004746	-1.31	5.24E-05	1.51E-05	-3.73E-05	6.19E-03
CIR1	NP_004873	-0.22	2.56E-05	9.07E-06	-1.65E-05	6.52E-03
HNRNPC	NP_112604	-0.97	1.38E-04	6.56E-05	-7.23E-05	6.56E-03
HSPA8	NP_006588	-1.28	7.15E-04	5.35E-04	-1.79E-04	7.09E-03
SEPT2	NP_004395	-1.02	1.94E-04	1.50E-04	-4.31E-05	7.52E-03
FLG2	NP_001014364	-0.33	1.98E-05	1.42E-05	-5.58E-06	7.83E-03
COQ2	NP_056512	-0.62	6.27E-05	4.40E-05	-1.86E-05	8.19E-03
PAFAH1B2	NP_002563	-0.94	2.13E-05	1.48E-05	-6.49E-06	8.43E-03
NKTR	NP_005376	-0.31	1.04E-04	7.38E-05	-3.07E-05	8.45E-03
PLEKHO2	NP_001181988	-0.53	4.92E-05	3.09E-05	-1.83E-05	8.52E-03
SEC24D	NP_055637	-0.62	3.76E-05	2.56E-05	-1.21E-05	8.52E-03
LMAN1	NP_005561	-0.37	2.14E-04	1.42E-04	-7.19E-05	9.04E-03
ITSN1	NP_001001132	-0.41	1.95E-05	7.95E-06	-1.15E-05	9.45E-03
ZNF362	NP_689706	-0.84	2.62E-05	8.99E-06	-1.73E-05	9.47E-03
GKAP1	NP_001129425	-0.70	2.58E-05	1.20E-05	-1.38E-05	9.93E-03
RBL1	NP_899662	-0.77	2.40E-05	1.08E-05	-1.32E-05	9.96E-03
MAGI3	NP_001136254	-1.21	6.99E-05	4.91E-05	-2.08E-05	1.00E-02
RAB5B	NP_002859	-0.98	4.41E-04	3.03E-04	-1.38E-04	1.03E-02
PDIK1L	NP_690048	-0.92	1.71E-05	6.95E-06	-1.02E-05	1.08E-02
CALD1	NP_149129	-1.32	4.01E-04	2.30E-04	-1.71E-04	1.13E-02
IKBIP	NP_710154	-0.85	1.08E-04	6.84E-05	-3.96E-05	1.15E-02
H2AFV	NP_619541	-0.31	7.13E-06	3.77E-06	-3.36E-06	1.17E-02
TMCC1	NP_001017395	-0.47	9.96E-05	1.66E-05	-8.31E-05	1.24E-02
SORL1	NP_003096	-0.81	3.49E-05	2.19E-05	-1.29E-05	1.35E-02
KIAA1109	NP_056127	-0.21	2.99E-04	2.46E-04	-5.31E-05	1.40E-02
KCNJ8	NP_004973	-0.85	1.61E-05	8.29E-06	-7.83E-06	1.46E-02
VASH2	NP_001129947	-0.47	1.92E-04	9.82E-05	-9.34E-05	1.51E-02
IQCE	NP_689771	-0.20	8.70E-05	4.53E-05	-4.18E-05	1.53E-02

LATS2	NP_055387	-0.21	1.61E-04	1.04E-04	-5.65E-05	1.57E-02
IFRD1	NP_001541	-0.91	1.05E-04	8.19E-05	-2.34E-05	1.57E-02
TRPS1	NP_054831	-0.25	6.73E-05	5.49E-05	-1.24E-05	1.60E-02
NEFH	NP_066554	-0.21	2.58E-05	1.94E-05	-6.45E-06	1.65E-02
ACSM5	NP_060358	-0.37	8.77E-05	5.29E-05	-3.47E-05	1.68E-02
BDH1	NP_976059	-0.50	3.36E-05	2.66E-05	-6.98E-06	1.74E-02
MTHFD1	NP_005947	-0.98	5.37E-05	4.33E-05	-1.03E-05	1.76E-02
CCDC150	NP_001074008	-0.27	5.16E-05	4.72E-05	-4.38E-06	1.77E-02
TACC2	NP_008928	-0.91	2.17E-05	1.54E-05	-6.30E-06	1.82E-02
SULF1	NP_055985	-0.65	1.11E-05	6.48E-06	-4.57E-06	1.85E-02
SMARCA5	NP_003592	-0.32	2.66E-04	1.80E-04	-8.64E-05	1.89E-02
ERG	NP_001129626	-0.79	2.57E-05	1.33E-05	-1.24E-05	1.92E-02
EPB41L4B	NP_060894	-0.70	2.20E-05	1.54E-05	-6.58E-06	1.94E-02
MAP3K12	NP_006292	-0.63	1.80E-05	1.04E-05	-7.59E-06	1.97E-02
ANKRD42	NP_872409	-0.44	3.12E-05	2.07E-05	-1.05E-05	2.04E-02
SYNC	NP_001155180	-0.44	2.68E-04	1.83E-04	-8.50E-05	2.05E-02
SYNE2	NP_878918	-0.51	1.38E-04	1.02E-04	-3.66E-05	2.08E-02
PLIN1	NP_001138783	-0.51	5.44E-06	3.29E-06	-2.16E-06	2.14E-02
UBE3C	NP_055486	-1.29	2.87E-05	2.02E-05	-8.44E-06	2.15E-02
PXDN	NP_036425	-0.98	5.53E-06	3.89E-06	-1.64E-06	2.31E-02
NAP1L1	NP_004528	-0.25	1.93E-05	8.63E-06	-1.07E-05	2.35E-02
CD109	NP_598000	-0.44	3.01E-05	1.72E-05	-1.28E-05	2.44E-02
ANKS1B	NP_690001	-1.06	2.50E-04	2.06E-04	-4.33E-05	2.51E-02
SEZ6	NP_849191	-0.47	4.24E-05	2.42E-05	-1.81E-05	2.58E-02
C3orf70	NP_001020437	-0.42	5.92E-05	1.97E-05	-3.95E-05	2.64E-02
WAC	NP_567823	-0.92	1.16E-03	7.24E-04	-4.41E-04	2.65E-02
CYP2U1	NP_898898	-0.58	3.03E-05	2.67E-05	-3.66E-06	2.78E-02
HNRNPA1	NP_002127	-0.61	6.01E-05	4.06E-05	-1.94E-05	2.80E-02
MYO5B	NP_001073936	-0.79	4.18E-04	1.79E-04	-2.38E-04	2.82E-02
DPP8	NP_569118	-0.38	5.25E-05	2.21E-05	-3.04E-05	2.83E-02
NECAB3	NP_112509	-0.21	5.21E-06	4.05E-06	-1.16E-06	2.96E-02
GOLGB1	NP_004478	-0.34	1.10E-05	5.72E-06	-5.31E-06	2.96E-02
AP4E1	NP_031373	-0.99	1.63E-05	9.88E-06	-6.44E-06	2.97E-02
KAT2B	NP_003875	-1.10	4.13E-04	3.74E-04	-3.89E-05	3.12E-02
DDX5	NP_004387	-0.89	3.92E-05	2.32E-05	-1.60E-05	3.15E-02
PDIA5	NP_006801	-0.40	3.02E-05	1.97E-05	-1.06E-05	3.28E-02
SETDB2	NP_001153780	-0.30	9.39E-05	6.74E-05	-2.64E-05	3.37E-02
CLUL1	NP_055225	-0.44	1.31E-05	9.54E-06	-3.52E-06	3.38E-02
KIF5C	NP_004513	-0.65	3.74E-05	2.77E-05	-9.65E-06	3.40E-02
DOCK5	NP_079216	-0.37	1.15E-05	6.57E-06	-4.96E-06	3.42E-02
ZMYM1	NP_079048	-0.70	9.78E-05	5.86E-05	-3.92E-05	3.44E-02
SNRPD3	NP_004166	-0.32	5.81E-05	3.88E-05	-1.93E-05	3.45E-02
CPNE1	NP_690904	-0.88	2.21E-05	1.71E-05	-4.99E-06	3.47E-02

KIF23	NP_004847	-1.22	3.96E-06	1.79E-06	-2.17E-06	3.52E-02
ZNF462	NP_067047	-0.68	2.38E-04	1.93E-04	-4.48E-05	3.55E-02
TAGAP	NP_473455	-0.94	4.99E-05	3.21E-05	-1.78E-05	3.69E-02
BCL6	NP_001124317	-0.32	2.67E-05	2.01E-05	-6.50E-06	3.80E-02
ZEB1	NP_001121600	-0.32	7.96E-06	5.67E-06	-2.29E-06	3.82E-02
RBM20	NP_001127835	-0.82	2.52E-05	1.78E-05	-7.44E-06	4.00E-02
AHCTF1	NP_056261	-1.06	1.25E-04	1.01E-04	-2.43E-05	4.08E-02
LASP1	NP_006139	-0.36	5.47E-05	4.39E-05	-1.09E-05	4.14E-02
TLR8	NP_619542	-0.72	8.64E-06	4.37E-06	-4.27E-06	4.20E-02
KLHL2	NP_001154993	-1.08	4.48E-05	1.86E-05	-2.62E-05	4.33E-02
CAB39L	NP_112187	-0.44	1.99E-05	1.25E-05	-7.32E-06	4.40E-02
RALYL	NP_001093861	-1.20	3.22E-05	1.78E-05	-1.45E-05	4.43E-02
PIP4K2C	NP_001139731	-0.52	4.63E-05	3.60E-05	-1.03E-05	4.54E-02
SKI	NP_003027	-0.59	1.92E-05	1.43E-05	-4.92E-06	4.56E-02
ZC3H11A	NP_055642	-0.23	1.57E-04	8.50E-05	-7.22E-05	4.59E-02
DPY19L1	NP_056098	-0.23	3.63E-05	2.16E-05	-1.47E-05	4.62E-02
PCDH15	NP_001136239	-0.77	1.09E-05	7.87E-06	-3.00E-06	4.72E-02
PRDM6	NP_001129711	-1.22	1.93E-04	1.51E-04	-4.26E-05	4.72E-02
FAF2	NP_055428	-0.46	9.62E-06	8.20E-06	-1.42E-06	4.90E-02
TMEM87A	NP_056312	-0.47	2.92E-05	2.12E-05	-8.01E-06	4.95E-02

mir-18a-5p (MIMAT0000072)						
**Gene Symbol**	**RefSeq ID**	**mirSVR Score**	**SR**	**SEN**	**Diff Mean**	***P*-value**
CTNNA1	NP_001894	-0.52	8.46E-05	6.23E-05	-2.23E-05	1.01E-05
ANXA5	NP_001145	-0.36	7.40E-04	3.08E-04	-4.32E-04	4.28E-05
PCSK6	NP_612193	-0.24	3.60E-05	1.05E-05	-2.55E-05	6.86E-05
EPDR1	NP_060019	-0.26	2.78E-05	1.43E-05	-1.35E-05	9.83E-05
RNASE9	NP_001103831	-1.12	1.94E-05	7.13E-06	-1.22E-05	1.41E-04
MATR3	NP_061322	-1.21	4.91E-05	2.33E-05	-2.57E-05	1.49E-04
ANXA7	NP_001147	-0.72	3.71E-05	7.51E-06	-2.96E-05	1.93E-04
VCP	NP_009057	-0.67	1.82E-04	1.41E-04	-4.14E-05	2.31E-04
LRBA	NP_006717	-0.74	5.36E-05	3.31E-05	-2.05E-05	5.55E-04
HNRNPR	NP_005817	-0.32	9.09E-05	5.69E-05	-3.40E-05	8.82E-04
ALS2CR11	NP_001161693	-0.39	2.70E-05	1.68E-05	-1.02E-05	9.11E-04
NRP1	NP_003864	-0.30	5.15E-05	2.84E-05	-2.31E-05	9.36E-04
BCAR3	NP_003558	-1.25	5.82E-05	4.24E-05	-1.57E-05	1.02E-03
TLL2	NP_036597	-0.64	1.37E-05	8.27E-06	-5.39E-06	1.09E-03
TCTEX1D1	NP_689878	-1.06	1.36E-05	5.81E-06	-7.81E-06	1.10E-03
ESR1	NP_000116	-0.70	3.86E-05	1.47E-05	-2.39E-05	1.11E-03
UBA6	NP_060697	-0.59	2.59E-05	1.32E-05	-1.27E-05	1.24E-03
TAOK2	NP_057235	-0.40	1.14E-04	7.57E-05	-3.85E-05	1.25E-03

COL8A2	NP_005193	-0.21	5.78E-05	3.13E-05	-2.65E-05	1.28E-03
EXOSC10	NP_001001998	-1.12	5.72E-05	4.32E-05	-1.40E-05	1.42E-03
CHD2	NP_001262	-0.67	4.19E-05	1.49E-05	-2.70E-05	1.49E-03
RIT1	NP_008843	-0.89	1.67E-05	7.30E-06	-9.41E-06	1.52E-03
ALB	NP_000468	-0.57	1.15E-03	7.41E-04	-4.08E-04	2.18E-03
RC3H2	NP_001094058	-0.45	2.38E-04	9.35E-05	-1.44E-04	2.44E-03
DDX3Y	NP_001116137	-0.25	1.47E-05	5.02E-06	-9.64E-06	2.54E-03
CCT5	NP_036205	-0.68	3.76E-05	2.72E-05	-1.04E-05	2.60E-03
RUVBL1	NP_003698	-1.13	1.80E-04	1.49E-04	-3.07E-05	2.68E-03
CCDC8	NP_114429	-0.71	3.31E-05	2.15E-05	-1.16E-05	2.85E-03
ABCC3	NP_003777	-0.48	1.84E-05	6.58E-06	-1.19E-05	2.98E-03
TARDBP	NP_031401	-0.35	2.35E-05	1.80E-05	-5.47E-06	3.25E-03
ACACB	NP_001084	-0.31	4.19E-05	3.38E-05	-8.10E-06	3.30E-03
DDX43	NP_061135	-0.23	5.40E-05	3.45E-05	-1.95E-05	3.62E-03
HNRNPUL1	NP_008971	-0.46	3.21E-05	1.89E-05	-1.32E-05	3.66E-03
ACACA	NP_942131	-0.47	2.59E-05	1.28E-05	-1.31E-05	4.07E-03
RBBP6	NP_008841	-1.24	2.61E-05	1.61E-05	-1.00E-05	4.07E-03
KCTD12	NP_612453	-0.35	2.45E-05	1.42E-05	-1.03E-05	4.32E-03
ZHX2	NP_055758	-0.78	5.23E-05	3.02E-05	-2.21E-05	4.43E-03
MAFB	NP_005452	-0.23	4.03E-05	2.23E-05	-1.81E-05	4.46E-03
CCDC88A	NP_001129069	-0.34	9.08E-05	6.46E-05	-2.62E-05	4.77E-03
STXBP3	NP_009200	-0.53	1.47E-05	8.20E-06	-6.50E-06	4.98E-03
SEC24C	NP_940999	-0.22	2.01E-04	1.09E-04	-9.14E-05	5.67E-03
RPS6KA5	NP_004746	-0.98	5.24E-05	1.51E-05	-3.73E-05	6.19E-03
ARF6	NP_001654	-1.10	2.23E-05	1.46E-05	-7.69E-06	6.32E-03
AKR1D1	NP_005980	-0.27	4.21E-05	2.83E-06	-3.92E-05	6.39E-03
SEPT2	NP_004395	-0.25	1.94E-04	1.50E-04	-4.31E-05	7.52E-03
NRL	NP_006168	-0.21	6.69E-05	3.30E-05	-3.39E-05	8.47E-03
TPM3	NP_705935	-0.31	7.60E-05	5.26E-05	-2.34E-05	8.49E-03
CDK5RAP2	NP_060719	-0.25	4.41E-05	2.89E-05	-1.52E-05	9.06E-03
ITSN1	NP_001001132	-0.64	1.95E-05	7.95E-06	-1.15E-05	9.45E-03
FBXO30	NP_115521	-0.31	1.44E-05	6.49E-06	-7.90E-06	9.70E-03
SEL1L3	NP_056002	-0.80	3.16E-05	1.67E-05	-1.49E-05	9.76E-03
MAGI3	NP_001136254	-0.53	6.99E-05	4.91E-05	-2.08E-05	1.00E-02
RAB5B	NP_002859	-0.22	4.41E-04	3.03E-04	-1.38E-04	1.03E-02
EFS	NP_005855	-0.81	4.56E-05	1.85E-05	-2.70E-05	1.06E-02
PDIK1L	NP_690048	-0.57	1.71E-05	6.95E-06	-1.02E-05	1.08E-02
PABPC4	NP_001129125	-0.30	3.90E-05	2.92E-05	-9.81E-06	1.11E-02
ANKLE2	NP_055929	-0.33	2.09E-04	1.24E-04	-8.45E-05	1.68E-02
FAM46B	NP_443175	-0.23	4.06E-05	1.83E-05	-2.23E-05	1.69E-02
CX3CL1	NP_002987	-0.26	2.71E-05	1.67E-05	-1.04E-05	1.75E-02
TAF15	NP_631961	-0.76	2.13E-05	1.39E-05	-7.48E-06	1.86E-02
EPB41L4B	NP_060894	-0.21	2.20E-05	1.54E-05	-6.58E-06	1.94E-02

CDC42	NP_001782	-1.21	1.34E-04	7.02E-05	-6.37E-05	2.05E-02
SYNC	NP_001155180	-0.64	2.68E-04	1.83E-04	-8.50E-05	2.05E-02
ICT1	NP_001536	-0.28	3.12E-04	1.33E-04	-1.80E-04	2.14E-02
NAP1L1	NP_004528	-0.21	1.93E-05	8.63E-06	-1.07E-05	2.35E-02
CTH	NP_001893	-0.39	1.33E-05	5.24E-06	-8.09E-06	2.38E-02
PSME4	NP_055429	-0.97	1.37E-05	9.08E-06	-4.58E-06	2.41E-02
NAA50	NP_079422	-0.69	3.77E-05	2.07E-05	-1.71E-05	2.43E-02
HNRNPA1	NP_002127	-0.92	6.01E-05	4.06E-05	-1.94E-05	2.80E-02
DOCK5	NP_079216	-0.74	1.15E-05	6.57E-06	-4.96E-06	3.42E-02
SNRPD3	NP_004166	-1.11	5.81E-05	3.88E-05	-1.93E-05	3.45E-02
GMIP	NP_057657	-1.20	2.21E-05	1.71E-05	-4.99E-06	3.47E-02
CIRH1A	NP_116219	-0.35	6.90E-06	4.55E-06	-2.35E-06	3.49E-02
VSIG4	NP_009199	-0.22	4.58E-05	1.93E-05	-2.65E-05	3.67E-02
TNK2	NP_001010938	-0.29	4.98E-05	3.22E-05	-1.76E-05	3.72E-02
TMCC2	NP_055673	-0.53	1.31E-05	7.49E-06	-5.61E-06	3.84E-02
PPP1R9A	NP_001159633	-0.35	1.44E-05	5.21E-06	-9.20E-06	3.87E-02
MKI67	NP_002408	-0.29	2.97E-04	2.61E-04	-3.63E-05	4.01E-02
AHCTF1	NP_056261	-0.50	1.25E-04	1.01E-04	-2.43E-05	4.08E-02
PYGB	NP_002853	-0.23	3.97E-06	1.59E-06	-2.38E-06	4.22E-02
KCNA1	NP_000208	-0.40	1.53E-05	8.08E-06	-7.20E-06	4.32E-02
CENPBD1	NP_659476	-0.45	1.09E-05	8.18E-06	-2.70E-06	4.49E-02
TBC1D22B	NP_060242	-0.36	4.74E-05	2.69E-05	-2.05E-05	4.53E-02
MAP4K3	NP_003609	-0.77	4.64E-05	3.07E-05	-1.57E-05	4.55E-02
RIMS2	NP_055492	-1.15	9.44E-05	6.87E-05	-2.58E-05	4.69E-02
PRDM6	NP_001129711	-1.15	1.93E-04	1.51E-04	-4.26E-05	4.72E-02
MYO1H	NP_001094891	-0.29	3.09E-05	2.39E-05	-6.93E-06	4.91E-02
PDCD6	NP_037364	-0.85	5.74E-06	3.26E-06	-2.48E-06	4.97E-02

mir-19a-3p (MIMAT0000073)						
**Gene Symbol**	**RefSeq ID**	**mirSVR Score**	**SR**	**SEN**	**Diff Mean**	***P*-value**
CTNNA1	NP_001894	-1.18	8.46E-05	6.23E-05	-2.23E-05	1.01E-05
PKD1L1	NP_612152	-0.99	1.20E-04	6.22E-05	-5.76E-05	1.24E-05
DNAJA2	NP_005871	-0.68	5.65E-06	3.04E-06	-2.61E-06	2.88E-05
SCN9A	NP_002968	-0.48	9.38E-05	5.27E-05	-4.11E-05	4.48E-05
PCSK6	NP_612193	-0.27	3.60E-05	1.05E-05	-2.55E-05	6.86E-05
ABCA5	NP_758424	-1.12	2.19E-05	7.82E-06	-1.40E-05	9.05E-05
SETX	NP_055861	-0.76	8.11E-05	3.78E-05	-4.33E-05	9.14E-05
JAG2	NP_002217	-0.51	3.58E-05	1.21E-05	-2.37E-05	1.09E-04
OR12D3	NP_112221	-0.95	1.58E-05	5.97E-06	-9.85E-06	1.54E-04
ANXA7	NP_001147	-0.61	3.71E-05	7.51E-06	-2.96E-05	1.93E-04
COL1A2	NP_000080	-1.14	8.86E-04	4.01E-04	-4.85E-04	1.98E-04

CRABP1	NP_004369	-0.39	1.04E-04	2.69E-05	-7.68E-05	2.12E-04
RCN2	NP_002893	-0.46	1.11E-05	6.78E-06	-4.30E-06	3.58E-04
ZEB2	NP_055610	-0.40	3.21E-05	8.87E-06	-2.32E-05	5.33E-04
KIAA1598	NP_060800	-0.80	5.37E-05	3.34E-05	-2.03E-05	5.48E-04
GRK6	NP_002073	-0.88	2.45E-05	1.25E-05	-1.21E-05	7.26E-04
DNM3	NP_001129599	-1.27	3.87E-05	2.77E-05	-1.09E-05	7.91E-04
E2F7	NP_976328	-1.02	1.93E-05	6.30E-06	-1.30E-05	9.19E-04
NRP1	NP_003864	-0.44	5.15E-05	2.84E-05	-2.31E-05	9.36E-04
BCAR3	NP_003558	-0.22	5.82E-05	4.24E-05	-1.57E-05	1.02E-03
TLL2	NP_036597	-0.70	1.37E-05	8.27E-06	-5.39E-06	1.09E-03
ESR1	NP_000116	-0.41	3.86E-05	1.47E-05	-2.39E-05	1.11E-03
ANTXR1	NP_444262	-0.39	1.15E-05	5.19E-06	-6.26E-06	1.19E-03
CS	NP_004068	-0.76	1.49E-04	8.20E-05	-6.70E-05	1.20E-03
DBN1	NP_004386	-1.16	7.88E-05	4.24E-05	-3.65E-05	1.36E-03
PBX2	NP_002577	-0.80	2.00E-05	5.62E-06	-1.44E-05	1.39E-03
CHD2	NP_001262	-1.00	4.19E-05	1.49E-05	-2.70E-05	1.49E-03
GRB10	NP_005302	-0.74	1.86E-05	9.90E-06	-8.73E-06	1.62E-03
SETD7	NP_085151	-0.31	8.13E-05	1.71E-05	-6.42E-05	1.66E-03
NCBP1	NP_002477	-0.62	1.11E-03	5.55E-04	-5.51E-04	1.75E-03
IGFBP3	NP_001013416	-1.11	1.22E-03	5.43E-04	-6.80E-04	1.81E-03
PABPC1L2B	NP_001035971	-0.35	6.46E-05	4.61E-05	-1.85E-05	1.84E-03
LRRC16A	NP_060110	-0.31	1.95E-05	1.37E-05	-5.84E-06	1.87E-03
ANXA4	NP_001144	-0.93	1.77E-04	9.63E-05	-8.11E-05	2.01E-03
ARID5B	NP_115575	-0.75	2.46E-05	1.97E-05	-4.90E-06	2.14E-03
DYNC1LI2	NP_006132	-0.93	2.42E-05	1.46E-05	-9.64E-06	2.14E-03
TGM2	NP_004604	-0.32	2.95E-04	2.21E-04	-7.42E-05	2.46E-03
DDX3Y	NP_001116137	-0.98	1.47E-05	5.02E-06	-9.64E-06	2.54E-03
ZC3H13	NP_055885	-1.05	8.71E-06	3.87E-06	-4.84E-06	2.54E-03
KIF1B	NP_055889	-0.34	2.81E-05	1.50E-05	-1.31E-05	2.56E-03
MECOM	NP_001098547	-1.10	2.44E-05	1.48E-05	-9.65E-06	2.74E-03
CCDC8	NP_114429	-0.24	3.31E-05	2.15E-05	-1.16E-05	2.85E-03
FAM178A	NP_001129595	-0.46	3.30E-05	2.15E-05	-1.15E-05	2.92E-03
DAAM1	NP_055807	-1.22	2.64E-04	1.03E-04	-1.61E-04	2.92E-03
ABCC3	NP_003777	-1.04	1.84E-05	6.58E-06	-1.19E-05	2.98E-03
HIVEP2	NP_006725	-0.24	1.31E-04	8.79E-05	-4.35E-05	3.01E-03
POP1	NP_001139333	-0.36	1.01E-04	4.89E-05	-5.17E-05	3.43E-03
ANKRD11	NP_037407	-0.33	4.89E-05	3.12E-05	-1.78E-05	3.56E-03
CAND1	NP_060918	-1.11	2.76E-04	1.33E-04	-1.43E-04	3.62E-03
HNRNPUL1	NP_008971	-0.87	3.21E-05	1.89E-05	-1.32E-05	3.66E-03
FAM84B	NP_777571	-0.41	5.26E-05	2.78E-05	-2.48E-05	3.77E-03
PLCXD2	NP_695000	-0.74	2.19E-05	1.50E-05	-6.91E-06	3.92E-03
ACACA	NP_942131	-0.31	2.59E-05	1.28E-05	-1.31E-05	4.07E-03
GPD2	NP_001076581	-0.22	7.59E-05	2.22E-05	-5.37E-05	4.20E-03

RPSA	NP_001012321	-0.69	2.06E-04	1.70E-04	-3.60E-05	4.69E-03
CCDC88A	NP_001129069	-1.06	9.08E-05	6.46E-05	-2.62E-05	4.77E-03
CLTC	NP_004850	-0.50	1.30E-03	1.17E-03	-1.33E-04	4.87E-03
HUWE1	NP_113584	-0.22	1.66E-04	1.18E-04	-4.77E-05	5.01E-03
MTF2	NP_001157864	-1.00	3.63E-05	2.65E-05	-9.79E-06	5.41E-03
PPM1K	NP_689755	-0.41	1.85E-05	1.20E-05	-6.48E-06	5.71E-03
TTF2	NP_003585	-0.48	1.06E-05	4.96E-06	-5.63E-06	5.99E-03
RPS6KA5	NP_004746	-1.02	5.24E-05	1.51E-05	-3.73E-05	6.19E-03
PALMD	NP_060204	-0.20	9.34E-06	6.89E-06	-2.45E-06	6.21E-03
SUZ12	NP_056170	-1.03	2.65E-05	1.54E-05	-1.12E-05	6.65E-03
TRIO	NP_009049	-0.41	3.60E-05	1.71E-05	-1.89E-05	7.69E-03
ZBBX	NP_078963	-0.20	1.34E-05	5.73E-06	-7.62E-06	7.95E-03
CXCL12	NP_954637	-0.48	2.43E-04	1.60E-04	-8.23E-05	7.98E-03
TUB	NP_003311	-0.68	1.56E-05	1.23E-05	-3.24E-06	8.01E-03
AHSG	NP_001613	-0.97	7.72E-05	3.88E-05	-3.84E-05	8.29E-03
PAFAH1B2	NP_002563	-0.61	2.13E-05	1.48E-05	-6.49E-06	8.43E-03
H2AFY	NP_001035248	-0.21	2.91E-04	1.62E-04	-1.29E-04	8.71E-03
KIAA1841	NP_001123465	-0.31	4.35E-05	2.68E-05	-1.67E-05	8.98E-03
CDK5RAP2	NP_060719	-0.29	4.41E-05	2.89E-05	-1.52E-05	9.06E-03
FECH	NP_000131	-0.21	3.31E-05	1.02E-05	-2.29E-05	9.07E-03
ITSN1	NP_001001132	-1.21	1.95E-05	7.95E-06	-1.15E-05	9.45E-03
MED13L	NP_056150	-0.97	8.49E-05	4.77E-05	-3.72E-05	9.63E-03
SEL1L3	NP_056002	-0.81	3.16E-05	1.67E-05	-1.49E-05	9.76E-03
GKAP1	NP_001129425	-0.30	2.58E-05	1.20E-05	-1.38E-05	9.93E-03
RBL1	NP_899662	-0.42	2.40E-05	1.08E-05	-1.32E-05	9.96E-03
DDX3X	NP_001180345	-1.07	2.18E-05	1.46E-05	-7.15E-06	1.03E-02
ARFGAP3	NP_055385	-0.93	5.09E-05	3.47E-05	-1.62E-05	1.03E-02
RAB5B	NP_002859	-0.47	4.41E-04	3.03E-04	-1.38E-04	1.03E-02
ZNF518A	NP_055618	-1.20	9.54E-04	4.97E-04	-4.56E-04	1.04E-02
PDIK1L	NP_690048	-0.81	1.71E-05	6.95E-06	-1.02E-05	1.08E-02
IGF2BP3	NP_006538	-0.93	1.92E-05	1.32E-05	-5.99E-06	1.11E-02
CALD1	NP_149129	-0.70	4.01E-04	2.30E-04	-1.71E-04	1.13E-02
BCL3	NP_005169	-0.31	2.10E-05	1.24E-05	-8.55E-06	1.14E-02
ATP8A1	NP_006086	-0.39	1.06E-04	4.58E-05	-5.98E-05	1.14E-02
ZBTB11	NP_055230	-1.11	1.34E-04	1.05E-04	-2.90E-05	1.48E-02
IQCE	NP_689771	-0.87	8.70E-05	4.53E-05	-4.18E-05	1.53E-02
SAP18	NP_005861	-0.51	1.81E-04	1.19E-04	-6.25E-05	1.55E-02
AKAP1	NP_003479	-0.23	2.57E-05	1.36E-05	-1.20E-05	1.57E-02
TRPS1	NP_054831	-0.22	6.73E-05	5.49E-05	-1.24E-05	1.60E-02
SPATS2L	NP_001093894	-0.63	1.62E-05	1.11E-05	-5.11E-06	1.66E-02
RELN	NP_005036	-1.28	4.53E-06	3.30E-06	-1.23E-06	1.69E-02
FAM46B	NP_443175	-0.94	4.06E-05	1.83E-05	-2.23E-05	1.69E-02
RNF141	NP_057506	-1.09	2.10E-04	1.25E-04	-8.45E-05	1.77E-02

SULF1	NP_055985	-0.85	1.11E-05	6.48E-06	-4.57E-06	1.85E-02
ERG	NP_001129626	-0.36	2.57E-05	1.33E-05	-1.24E-05	1.92E-02
MAP3K12	NP_006292	-0.88	1.80E-05	1.04E-05	-7.59E-06	1.97E-02
EIF4E3	NP_001128123	-0.20	2.58E-05	1.35E-05	-1.23E-05	2.01E-02
ANKRD42	NP_872409	-1.25	3.12E-05	2.07E-05	-1.05E-05	2.04E-02
ZNHIT6	NP_060423	-0.38	7.97E-05	1.50E-05	-6.48E-05	2.20E-02
LBR	NP_002287	-0.90	1.08E-05	4.98E-06	-5.83E-06	2.21E-02
EEF1A1	NP_001393	-1.30	1.81E-03	1.44E-03	-3.64E-04	2.21E-02
RFTN1	NP_055965	-0.54	3.05E-04	1.25E-04	-1.79E-04	2.25E-02
MAP7D2	NP_001161937	-0.60	2.26E-05	1.26E-05	-1.00E-05	2.27E-02
PXDN	NP_036425	-0.73	5.53E-06	3.89E-06	-1.64E-06	2.31E-02
NAP1L1	NP_004528	-0.50	1.93E-05	8.63E-06	-1.07E-05	2.35E-02
CSMD2	NP_443128	-0.33	8.05E-05	5.85E-05	-2.21E-05	2.39E-02
CMYA5	NP_705838	-0.69	4.96E-05	3.41E-05	-1.55E-05	2.42E-02
ANKS1B	NP_690001	-0.82	2.50E-04	2.06E-04	-4.33E-05	2.51E-02
C3orf70	NP_001020437	-0.91	5.92E-05	1.97E-05	-3.95E-05	2.64E-02
WAC	NP_567823	-0.90	1.16E-03	7.24E-04	-4.41E-04	2.65E-02
ATP6V0A1	NP_001123493	-0.93	5.56E-05	3.58E-05	-1.98E-05	2.69E-02
DPM1	NP_003850	-0.35	9.42E-05	4.33E-05	-5.09E-05	2.72E-02
SNX18	NP_001138899	-0.23	1.32E-05	9.50E-06	-3.69E-06	2.74E-02
CYP2U1	NP_898898	-1.06	3.03E-05	2.67E-05	-3.66E-06	2.78E-02
HNRNPA1	NP_002127	-1.11	6.01E-05	4.06E-05	-1.94E-05	2.80E-02
MYO5B	NP_001073936	-0.64	4.18E-04	1.79E-04	-2.38E-04	2.82E-02
AFTPH	NP_060127	-1.31	2.39E-05	8.29E-06	-1.56E-05	2.85E-02
NECAB3	NP_112509	-0.71	5.21E-06	4.05E-06	-1.16E-06	2.96E-02
AP4E1	NP_031373	-0.28	1.63E-05	9.88E-06	-6.44E-06	2.97E-02
INSM2	NP_115983	-1.00	7.83E-05	5.52E-05	-2.31E-05	3.04E-02
CASP1	NP_150637	-1.22	9.13E-05	8.01E-05	-1.12E-05	3.09E-02
PDE4A	NP_006193	-0.44	3.28E-05	1.91E-05	-1.37E-05	3.10E-02
VANGL2	NP_065068	-0.28	2.13E-05	1.59E-05	-5.36E-06	3.10E-02
ARHGAP11A	NP_055598	-1.19	3.57E-05	2.67E-05	-9.02E-06	3.11E-02
CGNL1	NP_116255	-0.72	2.45E-05	2.08E-05	-3.77E-06	3.15E-02
CLUL1	NP_055225	-1.19	1.31E-05	9.54E-06	-3.52E-06	3.38E-02
KIF5C	NP_004513	-0.68	3.74E-05	2.77E-05	-9.65E-06	3.40E-02
PPP1R12A	NP_001137357	-1.09	1.25E-04	8.27E-05	-4.26E-05	3.42E-02
DOCK5	NP_079216	-0.80	1.15E-05	6.57E-06	-4.96E-06	3.42E-02
SNRPD3	NP_004166	-0.50	5.81E-05	3.88E-05	-1.93E-05	3.45E-02
PPP1R9A	NP_001159633	-0.23	1.44E-05	5.21E-06	-9.20E-06	3.87E-02
RBM20	NP_001127835	-0.48	2.52E-05	1.78E-05	-7.44E-06	4.00E-02
AHCTF1	NP_056261	-1.10	1.25E-04	1.01E-04	-2.43E-05	4.08E-02
PYGB	NP_002853	-0.31	3.97E-06	1.59E-06	-2.38E-06	4.22E-02
CAB39L	NP_112187	-0.44	1.99E-05	1.25E-05	-7.32E-06	4.40E-02
TBC1D22B	NP_060242	-0.36	4.74E-05	2.69E-05	-2.05E-05	4.53E-02

MAP4K3	NP_003609	-1.22	4.64E-05	3.07E-05	-1.57E-05	4.55E-02
ZC3H11A	NP_055642	-0.87	1.57E-04	8.50E-05	-7.22E-05	4.59E-02
FAM193A	NP_003695	-0.40	9.01E-05	5.01E-05	-3.99E-05	4.70E-02
POSTN	NP_006466	-0.71	2.83E-04	2.17E-04	-6.56E-05	4.71E-02
PCDH15	NP_001136239	-0.87	1.09E-05	7.87E-06	-3.00E-06	4.72E-02
CEP350	NP_055625	-1.15	7.84E-05	6.00E-05	-1.84E-05	4.94E-02
PDCD6	NP_037364	-0.49	5.74E-06	3.26E-06	-2.48E-06	4.97E-02

**mir-20a-5p (MIMAT0000075)**						
**Gene Symbol**	**RefSeq ID**	**mirSVR Score**	**SR**	**SEN**	**Diff Mean**	***P*-value**
SH3GLB1	NP_057093	-0.44	1.92E-05	6.38E-06	-1.28E-05	1.18E-04
DNALI1	NP_003453	-0.20	3.16E-05	1.51E-05	-1.65E-05	1.61E-04
LCA5L	NP_689718	-0.32	5.13E-05	1.36E-05	-3.77E-05	1.87E-04
CCDC141	NP_775919	-0.46	1.84E-05	8.26E-06	-1.01E-05	3.15E-04
RCN2	NP_002893	-0.21	1.11E-05	6.78E-06	-4.30E-06	3.58E-04
IGFBP7	NP_001544	-0.23	1.20E-05	4.12E-06	-7.89E-06	3.70E-04
CA10	NP_001076002	-0.66	2.84E-05	7.68E-06	-2.08E-05	4.35E-04
ZEB2	NP_055610	-0.56	3.21E-05	8.87E-06	-2.32E-05	5.33E-04
KIAA1598	NP_060800	-1.25	5.37E-05	3.34E-05	-2.03E-05	5.48E-04
NUP205	NP_055950	-1.28	2.54E-05	1.12E-05	-1.43E-05	5.66E-04
MAP2	NP_002365	-0.33	8.61E-05	3.58E-05	-5.03E-05	5.76E-04
FGD5	NP_689749	-1.19	1.52E-05	9.77E-06	-5.40E-06	6.26E-04
COL15A1	NP_001846	-0.83	3.44E-05	1.26E-05	-2.18E-05	7.09E-04
AHNAK	NP_001611	-1.29	2.98E-03	2.02E-03	-9.63E-04	7.28E-04
U2AF1	NP_001020374	-0.24	7.00E-05	3.10E-05	-3.90E-05	9.26E-04
TCTEX1D1	NP_689878	-0.89	1.36E-05	5.81E-06	-7.81E-06	1.10E-03
ESR1	NP_000116	-0.40	3.86E-05	1.47E-05	-2.39E-05	1.11E-03
KTN1	NP_001072989	-0.31	2.25E-04	1.51E-04	-7.40E-05	1.18E-03
ANTXR1	NP_444262	-0.48	1.15E-05	5.19E-06	-6.26E-06	1.19E-03
UBA6	NP_060697	-0.35	2.59E-05	1.32E-05	-1.27E-05	1.24E-03
TAOK2	NP_057235	-1.12	1.14E-04	7.57E-05	-3.85E-05	1.25E-03
VASP	NP_003361	-0.22	1.75E-05	8.17E-06	-9.37E-06	1.26E-03
ATXN2	NP_002964	-1.08	1.17E-05	5.04E-06	-6.65E-06	1.31E-03
SIN3A	NP_001138829	-0.54	1.10E-04	8.09E-05	-2.93E-05	1.35E-03
CHD2	NP_001262	-0.78	4.19E-05	1.49E-05	-2.70E-05	1.49E-03
RIT1	NP_008843	-0.50	1.67E-05	7.30E-06	-9.41E-06	1.52E-03
RB1	NP_000312	-0.72	3.99E-04	2.18E-04	-1.81E-04	1.56E-03
HN1	NP_057269	-1.29	1.86E-05	9.90E-06	-8.73E-06	1.64E-03
SUV39H2	NP_001180353	-0.48	4.11E-04	2.37E-04	-1.74E-04	1.80E-03
CHD4	NP_001264	-0.73	9.12E-06	2.78E-06	-6.34E-06	1.97E-03
ARID5B	NP_115575	-0.30	2.46E-05	1.97E-05	-4.90E-06	2.14E-03

DYNC1LI2	NP_006132	-1.14	2.42E-05	1.46E-05	-9.64E-06	2.14E-03
FTL	NP_000137	-0.64	8.54E-06	3.95E-06	-4.59E-06	2.24E-03
TGM2	NP_004604	-1.09	2.95E-04	2.21E-04	-7.42E-05	2.46E-03
ETF1	NP_004721	-0.30	3.52E-05	2.30E-05	-1.22E-05	2.47E-03
CCT5	NP_036205	-0.44	3.76E-05	2.72E-05	-1.04E-05	2.60E-03
NEB	NP_001157980	-0.24	4.45E-04	2.92E-04	-1.53E-04	2.63E-03
ARID1B	NP_059989	-0.98	2.15E-04	1.20E-04	-9.52E-05	2.93E-03
DSTYK	NP_955749	-0.24	1.00E-04	4.89E-05	-5.15E-05	3.28E-03
ANKRD11	NP_037407	-0.23	4.89E-05	3.12E-05	-1.78E-05	3.56E-03
TRIM22	NP_006065	-0.99	8.68E-06	3.86E-06	-4.82E-06	3.69E-03
CHD9	NP_079410	-1.00	4.29E-04	9.94E-05	-3.30E-04	3.92E-03
PDE8A	NP_002596	-0.24	8.89E-05	6.25E-05	-2.64E-05	4.05E-03
RBBP6	NP_008841	-0.43	2.61E-05	1.61E-05	-1.00E-05	4.07E-03
GPD2	NP_001076581	-0.29	7.59E-05	2.22E-05	-5.37E-05	4.20E-03
ZHX2	NP_055758	-0.76	5.23E-05	3.02E-05	-2.21E-05	4.43E-03
CTSA	NP_001121167	-0.94	4.21E-05	3.13E-05	-1.08E-05	4.60E-03
CCDC88A	NP_001129069	-0.83	9.08E-05	6.46E-05	-2.62E-05	4.77E-03
TIAM1	NP_003244	-1.25	6.62E-05	3.46E-05	-3.16E-05	4.82E-03
TOPORS	NP_005793	-1.08	2.14E-04	1.49E-04	-6.42E-05	4.91E-03
ACCS	NP_115981	-0.47	1.63E-05	9.45E-06	-6.80E-06	4.91E-03
NPM1	NP_002511	-1.05	5.48E-05	2.47E-05	-3.01E-05	5.35E-03
JAK3	NP_000206	-0.65	2.73E-05	1.11E-05	-1.62E-05	5.41E-03
MSR1	NP_619729	-1.11	3.63E-05	2.64E-05	-9.85E-06	5.53E-03
RPS6KA5	NP_004746	-1.29	5.24E-05	1.51E-05	-3.73E-05	6.19E-03
CIR1	NP_004873	-0.22	2.56E-05	9.07E-06	-1.65E-05	6.52E-03
HNRNPC	NP_112604	-0.97	1.38E-04	6.56E-05	-7.23E-05	6.56E-03
NDUFA4	NP_002480	-1.00	9.62E-06	7.11E-06	-2.52E-06	6.68E-03
HSPA8	NP_006588	-1.28	7.15E-04	5.35E-04	-1.79E-04	7.09E-03
SEPT2	NP_004395	-1.01	1.94E-04	1.50E-04	-4.31E-05	7.52E-03
FLG2	NP_001014364	-0.33	1.98E-05	1.42E-05	-5.58E-06	7.83E-03
COQ2	NP_056512	-0.62	6.27E-05	4.40E-05	-1.86E-05	8.19E-03
PAFAH1B2	NP_002563	-0.94	2.13E-05	1.48E-05	-6.49E-06	8.43E-03
NKTR	NP_005376	-0.31	1.04E-04	7.38E-05	-3.07E-05	8.45E-03
PLEKHO2	NP_001181988	-0.40	4.92E-05	3.09E-05	-1.83E-05	8.52E-03
SEC24D	NP_055637	-0.63	3.76E-05	2.56E-05	-1.21E-05	8.52E-03
LMAN1	NP_005561	-0.37	2.14E-04	1.42E-04	-7.19E-05	9.04E-03
ZNF362	NP_689706	-0.84	2.62E-05	8.99E-06	-1.73E-05	9.47E-03
FBXO30	NP_115521	-0.60	1.44E-05	6.49E-06	-7.90E-06	9.70E-03
GKAP1	NP_001129425	-0.70	2.58E-05	1.20E-05	-1.38E-05	9.93E-03
RBL1	NP_899662	-0.77	2.40E-05	1.08E-05	-1.32E-05	9.96E-03
MAGI3	NP_001136254	-1.21	6.99E-05	4.91E-05	-2.08E-05	1.00E-02
RAB5B	NP_002859	-0.96	4.41E-04	3.03E-04	-1.38E-04	1.03E-02
PDIK1L	NP_690048	-0.93	1.71E-05	6.95E-06	-1.02E-05	1.08E-02

CALD1	NP_149129	-1.32	4.01E-04	2.30E-04	-1.71E-04	1.13E-02
IKBIP	NP_710154	-0.84	1.08E-04	6.84E-05	-3.96E-05	1.15E-02
H2AFV	NP_619541	-0.31	7.13E-06	3.77E-06	-3.36E-06	1.17E-02
TMCC1	NP_001017395	-0.47	9.96E-05	1.66E-05	-8.31E-05	1.24E-02
SORL1	NP_003096	-0.81	3.49E-05	2.19E-05	-1.29E-05	1.35E-02
KIAA1109	NP_056127	-0.21	2.99E-04	2.46E-04	-5.31E-05	1.40E-02
KCNJ8	NP_004973	-0.85	1.61E-05	8.29E-06	-7.83E-06	1.46E-02
VASH2	NP_001129947	-0.47	1.92E-04	9.82E-05	-9.34E-05	1.51E-02
IQCE	NP_689771	-0.20	8.70E-05	4.53E-05	-4.18E-05	1.53E-02
LATS2	NP_055387	-0.21	1.61E-04	1.04E-04	-5.65E-05	1.57E-02
IFRD1	NP_001541	-0.90	1.05E-04	8.19E-05	-2.34E-05	1.57E-02
TRPS1	NP_054831	-0.25	6.73E-05	5.49E-05	-1.24E-05	1.60E-02
NEFH	NP_066554	-0.22	2.58E-05	1.94E-05	-6.45E-06	1.65E-02
ACSM5	NP_060358	-0.37	8.77E-05	5.29E-05	-3.47E-05	1.68E-02
BDH1	NP_976059	-0.50	3.36E-05	2.66E-05	-6.98E-06	1.74E-02
MTHFD1	NP_005947	-0.98	5.37E-05	4.33E-05	-1.03E-05	1.76E-02
CCDC150	NP_001074008	-0.27	5.16E-05	4.72E-05	-4.38E-06	1.77E-02
BTF3	NP_001032726	-0.20	1.83E-05	1.47E-05	-3.53E-06	1.80E-02
TACC2	NP_008928	-0.91	2.17E-05	1.54E-05	-6.30E-06	1.82E-02
SULF1	NP_055985	-0.65	1.11E-05	6.48E-06	-4.57E-06	1.85E-02
SMARCA5	NP_003592	-0.32	2.66E-04	1.80E-04	-8.64E-05	1.89E-02
CHGA	NP_001266	-0.63	3.87E-05	2.07E-05	-1.80E-05	1.91E-02
ERG	NP_001129626	-0.79	2.57E-05	1.33E-05	-1.24E-05	1.92E-02
EPB41L4B	NP_060894	-0.70	2.20E-05	1.54E-05	-6.58E-06	1.94E-02
MAP3K12	NP_006292	-0.63	1.80E-05	1.04E-05	-7.59E-06	1.97E-02
ANKRD42	NP_872409	-0.44	3.12E-05	2.07E-05	-1.05E-05	2.04E-02
SYNC	NP_001155180	-0.44	2.68E-04	1.83E-04	-8.50E-05	2.05E-02
SYNE2	NP_878918	-0.51	1.38E-04	1.02E-04	-3.66E-05	2.08E-02
PLIN1	NP_001138783	-0.51	5.44E-06	3.29E-06	-2.16E-06	2.14E-02
UBE3C	NP_055486	-1.29	2.87E-05	2.02E-05	-8.44E-06	2.15E-02
PXDN	NP_036425	-0.98	5.53E-06	3.89E-06	-1.64E-06	2.31E-02
MAP7D3	NP_078873	-0.64	6.86E-06	5.91E-06	-9.46E-07	2.35E-02
NAP1L1	NP_004528	-0.24	1.93E-05	8.63E-06	-1.07E-05	2.35E-02
CD109	NP_598000	-0.44	3.01E-05	1.72E-05	-1.28E-05	2.44E-02
ANKS1B	NP_690001	-1.06	2.50E-04	2.06E-04	-4.33E-05	2.51E-02
SEZ6	NP_849191	-0.48	4.24E-05	2.42E-05	-1.81E-05	2.58E-02
GAK	NP_005246	-0.25	6.45E-05	2.78E-05	-3.67E-05	2.63E-02
WAC	NP_567823	-0.92	1.16E-03	7.24E-04	-4.41E-04	2.65E-02
CYP2U1	NP_898898	-0.58	3.03E-05	2.67E-05	-3.66E-06	2.78E-02
MYO5B	NP_001073936	-0.79	4.18E-04	1.79E-04	-2.38E-04	2.82E-02
DPP8	NP_569118	-0.38	5.25E-05	2.21E-05	-3.04E-05	2.83E-02
NECAB3	NP_112509	-0.21	5.21E-06	4.05E-06	-1.16E-06	2.96E-02
GOLGB1	NP_004478	-0.34	1.10E-05	5.72E-06	-5.31E-06	2.96E-02

AP4E1	NP_031373	-0.99	1.63E-05	9.88E-06	-6.44E-06	2.97E-02
KAT2B	NP_003875	-1.10	4.13E-04	3.74E-04	-3.89E-05	3.12E-02
DDX5	NP_004387	-0.89	3.92E-05	2.32E-05	-1.60E-05	3.15E-02
PDIA5	NP_006801	-0.40	3.02E-05	1.97E-05	-1.06E-05	3.28E-02
SETDB2	NP_001153780	-0.30	9.39E-05	6.74E-05	-2.64E-05	3.37E-02
CLUL1	NP_055225	-0.44	1.31E-05	9.54E-06	-3.52E-06	3.38E-02
KIF5C	NP_004513	-0.66	3.74E-05	2.77E-05	-9.65E-06	3.40E-02
PPP1R12A	NP_001137357	-1.02	1.25E-04	8.27E-05	-4.26E-05	3.42E-02
DOCK5	NP_079216	-0.40	1.15E-05	6.57E-06	-4.96E-06	3.42E-02
ZMYM1	NP_079048	-0.70	9.78E-05	5.86E-05	-3.92E-05	3.44E-02
SNRPD3	NP_004166	-0.32	5.81E-05	3.88E-05	-1.93E-05	3.45E-02
CPNE1	NP_690904	-0.89	2.21E-05	1.71E-05	-4.99E-06	3.47E-02
KIF23	NP_004847	-1.22	3.96E-06	1.79E-06	-2.17E-06	3.52E-02
TAGAP	NP_473455	-0.94	4.99E-05	3.21E-05	-1.78E-05	3.69E-02
BCL6	NP_001124317	-0.32	2.67E-05	2.01E-05	-6.50E-06	3.80E-02
ZEB1	NP_001121600	-0.32	7.96E-06	5.67E-06	-2.29E-06	3.82E-02
RBM20	NP_001127835	-0.82	2.52E-05	1.78E-05	-7.44E-06	4.00E-02
AHCTF1	NP_056261	-1.06	1.25E-04	1.01E-04	-2.43E-05	4.08E-02
LASP1	NP_006139	-0.36	5.47E-05	4.39E-05	-1.09E-05	4.14E-02
TLR8	NP_619542	-0.72	8.64E-06	4.37E-06	-4.27E-06	4.20E-02
EXOSC3	NP_057126	-1.08	3.52E-05	2.12E-05	-1.39E-05	4.23E-02
KLHL2	NP_001154993	-1.08	4.48E-05	1.86E-05	-2.62E-05	4.33E-02
CAB39L	NP_112187	-0.44	1.99E-05	1.25E-05	-7.32E-06	4.40E-02
RALYL	NP_001093861	-1.19	3.22E-05	1.78E-05	-1.45E-05	4.43E-02
CENPBD1	NP_659476	-0.42	1.09E-05	8.18E-06	-2.70E-06	4.49E-02
PIP4K2C	NP_001139731	-0.52	4.63E-05	3.60E-05	-1.03E-05	4.54E-02
SKI	NP_003027	-0.59	1.92E-05	1.43E-05	-4.92E-06	4.56E-02
ZC3H11A	NP_055642	-0.23	1.57E-04	8.50E-05	-7.22E-05	4.59E-02
DPY19L1	NP_056098	-0.23	3.63E-05	2.16E-05	-1.47E-05	4.62E-02
ZNF528	NP_115799	-0.49	1.74E-05	9.52E-06	-7.87E-06	4.67E-02
PCDH15	NP_001136239	-0.77	1.09E-05	7.87E-06	-3.00E-06	4.72E-02
PRDM6	NP_001129711	-1.22	1.93E-04	1.51E-04	-4.26E-05	4.72E-02
FAF2	NP_055428	-0.46	9.62E-06	8.20E-06	-1.42E-06	4.90E-02
TMEM87A	NP_056312	-0.21	2.92E-05	2.12E-05	-8.01E-06	4.95E-02

**mir-100-5p (MIMAT0000098)**						
**Gene Symbol**	**RefSeq ID**	**mirSVR Score**	**SR**	**SEN**	**Diff Mean**	***P*-value**
EPDR1	NP_060019	-0.37	2.78E-05	1.43E-05	-1.35E-05	9.83E-05
CCDC141	NP_775919	-0.59	1.84E-05	8.26E-06	-1.01E-05	3.15E-04
ST5	NP_631896	-0.36	2.01E-05	5.56E-06	-1.45E-05	1.28E-03
TARDBP	NP_031401	-0.92	2.35E-05	1.80E-05	-5.47E-06	3.25E-03

HNRNPH2	NP_062543	-0.41	3.11E-05	1.32E-05	-1.79E-05	5.38E-03
PTPRN2	NP_570857	-0.21	4.39E-05	2.90E-05	-1.49E-05	5.59E-03
VNN1	NP_004657	-0.97	6.24E-06	3.95E-06	-2.29E-06	1.35E-02
SMARCA5	NP_003592	-1.27	2.66E-04	1.80E-04	-8.64E-05	1.89E-02
HOXA1	NP_005513	-0.84	2.82E-06	1.53E-06	-1.30E-06	3.08E-02
NOP56	NP_006383	-0.29	8.98E-05	6.46E-05	-2.52E-05	3.27E-02
TBC1D22B	NP_060242	-0.51	4.74E-05	2.69E-05	-2.05E-05	4.53E-02

**mir-125b-5p (MIMAT0000423)**						
**Gene Symbol**	**RefSeq ID**	**mirSVR Score**	**SR**	**SEN**	**Diff Mean**	***P*-value**
PLCB2	NP_004564	-0.44	1.96E-05	1.38E-05	-5.79E-06	1.27E-05
COL1A2	NP_000080	-0.37	8.86E-04	4.01E-04	-4.85E-04	1.98E-04
ACSBG2	NP_112186	-0.40	1.96E-05	9.43E-06	-1.02E-05	3.52E-04
ZEB2	NP_055610	-0.59	3.21E-05	8.87E-06	-2.32E-05	5.33E-04
KIAA1598	NP_060800	-0.55	5.37E-05	3.34E-05	-2.03E-05	5.48E-04
CALCA	NP_001029124	-0.52	3.54E-04	1.72E-04	-1.81E-04	1.28E-03
EXOSC10	NP_001001998	-0.65	5.72E-05	4.32E-05	-1.40E-05	1.42E-03
RIT1	NP_008843	-0.51	1.67E-05	7.30E-06	-9.41E-06	1.52E-03
RB1	NP_000312	-0.51	3.99E-04	2.18E-04	-1.81E-04	1.56E-03
MUC5B	NP_002449	-0.21	4.56E-05	2.80E-05	-1.76E-05	1.84E-03
ZC3H13	NP_055885	-0.36	8.71E-06	3.87E-06	-4.84E-06	2.54E-03
HIVEP2	NP_006725	-0.38	1.31E-04	8.79E-05	-4.35E-05	3.01E-03
NAIF1	NP_931045	-0.25	1.89E-05	1.24E-05	-6.51E-06	4.23E-03
SLC25A35	NP_958928	-0.99	3.38E-05	1.70E-05	-1.69E-05	4.77E-03
CASC3	NP_031385	-0.26	1.08E-04	5.58E-05	-5.20E-05	5.14E-03
PLIN3	NP_005808	-0.32	1.89E-05	1.49E-05	-3.94E-06	5.70E-03
ARNTL	NP_001025444	-0.75	8.34E-05	4.87E-05	-3.48E-05	6.16E-03
AKR1D1	NP_005980	-0.40	4.21E-05	2.83E-06	-3.92E-05	6.39E-03
RGAG1	NP_065820	-0.32	7.88E-06	5.32E-06	-2.57E-06	7.03E-03
TRIO	NP_009049	-0.83	3.60E-05	1.71E-05	-1.89E-05	7.69E-03
KIAA1841	NP_001123465	-0.62	4.35E-05	2.68E-05	-1.67E-05	8.98E-03
CDK5RAP2	NP_060719	-0.72	4.41E-05	2.89E-05	-1.52E-05	9.06E-03
ITSN1	NP_001001132	-0.26	1.95E-05	7.95E-06	-1.15E-05	9.45E-03
EML6	NP_001034842	-0.29	3.57E-05	2.41E-05	-1.16E-05	1.01E-02
TOMM40	NP_006105	-0.21	4.57E-05	2.96E-05	-1.61E-05	1.13E-02
IKBIP	NP_710154	-0.38	1.08E-04	6.84E-05	-3.96E-05	1.15E-02
MYO15A	NP_057323	-0.42	4.59E-05	3.44E-05	-1.15E-05	1.33E-02
ARHGEF2	NP_004714	-0.33	2.66E-04	1.75E-04	-9.17E-05	1.46E-02
VASH2	NP_001129947	-0.49	1.92E-04	9.82E-05	-9.34E-05	1.51E-02
TRPS1	NP_054831	-0.31	6.73E-05	5.49E-05	-1.24E-05	1.60E-02
BDH1	NP_976059	-0.47	3.36E-05	2.66E-05	-6.98E-06	1.74E-02

RNF141	NP_057506	-0.77	2.10E-04	1.25E-04	-8.45E-05	1.77E-02
HNRNPA2B1	NP_112533	-0.32	1.33E-04	7.21E-05	-6.06E-05	1.84E-02
PDZD3	NP_079067	-1.09	4.08E-05	7.18E-06	-3.36E-05	1.93E-02
MAP3K12	NP_006292	-0.21	1.80E-05	1.04E-05	-7.59E-06	1.97E-02
PNPT1	NP_149100	-0.90	1.12E-05	7.93E-06	-3.30E-06	1.99E-02
MTMR14	NP_001070993	-0.78	1.15E-05	0.00E+00	-1.15E-05	2.00E-02
ANKRD42	NP_872409	-1.11	3.12E-05	2.07E-05	-1.05E-05	2.04E-02
PTPRS	NP_002841	-0.28	1.16E-05	4.98E-06	-6.62E-06	2.40E-02
ANPEP	NP_001141	-0.32	6.21E-04	5.01E-04	-1.20E-04	2.44E-02
CYP24A1	NP_000773	-0.73	3.33E-04	2.35E-04	-9.80E-05	2.90E-02
NECAB3	NP_112509	-0.39	5.21E-06	4.05E-06	-1.16E-06	2.96E-02
GOLGB1	NP_004478	-0.23	1.10E-05	5.72E-06	-5.31E-06	2.96E-02
ENTPD1	NP_001767	-0.22	3.15E-05	2.39E-05	-7.61E-06	3.01E-02
PPCDC	NP_068595	-0.29	1.78E-05	1.41E-05	-3.69E-06	3.17E-02
GMIP	NP_057657	-0.41	2.21E-05	1.71E-05	-4.99E-06	3.47E-02
KIF23	NP_004847	-0.45	3.96E-06	1.79E-06	-2.17E-06	3.52E-02
RBM20	NP_001127835	-0.79	2.52E-05	1.78E-05	-7.44E-06	4.00E-02
PCDH15	NP_001136239	-0.38	1.09E-05	7.87E-06	-3.00E-06	4.72E-02
PDCD6	NP_037364	-0.63	5.74E-06	3.26E-06	-2.48E-06	4.97E-02

**mir-92a-1-5p (MIMAT0004507)**						
**Gene Symbol**	**RefSeq ID**	**mirSVR Score**	**SR**	**SEN**	**Diff Mean**	***P*-value**
YWHAH	NP_003396	-0.24	6.61E-05	1.92E-05	-4.68E-05	8.56E-06
COL1A1	NP_000079	-0.75	2.23E-03	6.76E-04	-1.55E-03	7.25E-05
ABCA5	NP_758424	-0.39	2.19E-05	7.82E-06	-1.40E-05	9.05E-05
CNTN5	NP_780775	-0.20	1.04E-04	2.67E-05	-7.73E-05	1.95E-04
VIM	NP_003371	-0.29	5.05E-02	3.74E-02	-1.31E-02	2.04E-04
LMO7	NP_056667	-0.25	1.74E-05	7.66E-06	-9.71E-06	4.43E-04
ANO10	NP_060545	-0.27	4.65E-05	2.48E-05	-2.18E-05	6.53E-04
ANXA2	NP_001129487	-0.30	7.03E-03	4.42E-03	-2.61E-03	1.26E-03
LRCH4	NP_002310	-0.50	4.52E-05	2.59E-05	-1.93E-05	1.30E-03
ATXN2	NP_002964	-0.25	1.17E-05	5.04E-06	-6.65E-06	1.31E-03
SIN3A	NP_001138829	-0.39	1.10E-04	8.09E-05	-2.93E-05	1.35E-03
CHD2	NP_001262	-0.43	4.19E-05	1.49E-05	-2.70E-05	1.49E-03
PPEF2	NP_006230	-0.29	4.69E-05	2.04E-05	-2.65E-05	1.78E-03
CANX	NP_001737	-0.39	6.95E-04	4.89E-04	-2.06E-04	2.12E-03
FTL	NP_000137	-0.50	8.54E-06	3.95E-06	-4.59E-06	2.24E-03
HNRNPK	NP_112553	-0.91	3.12E-04	1.97E-04	-1.15E-04	2.68E-03
MECOM	NP_001098547	-1.28	2.44E-05	1.48E-05	-9.65E-06	2.74E-03
FA2H	NP_077282	-0.25	1.67E-04	8.13E-05	-8.53E-05	3.09E-03
NETO1	NP_620416	-0.84	2.85E-05	1.99E-05	-8.57E-06	3.44E-03

HNRNPUL1	NP_008971	-0.42	3.21E-05	1.89E-05	-1.32E-05	3.66E-03
PLCXD2	NP_695000	-0.25	2.19E-05	1.50E-05	-6.91E-06	3.92E-03
BGN	NP_001702	-0.22	8.52E-05	6.24E-05	-2.28E-05	4.83E-03
NFYC	NP_055038	-0.23	1.29E-04	4.74E-05	-8.18E-05	4.89E-03
HUWE1	NP_113584	-0.34	1.66E-04	1.18E-04	-4.77E-05	5.01E-03
JAK3	NP_000206	-0.35	2.73E-05	1.11E-05	-1.62E-05	5.41E-03
RRBP1	NP_004578	-0.32	1.90E-04	1.31E-04	-5.93E-05	5.62E-03
SEC24C	NP_940999	-0.51	2.01E-04	1.09E-04	-9.14E-05	5.67E-03
PPM1K	NP_689755	-0.48	1.85E-05	1.20E-05	-6.48E-06	5.71E-03
HAVCR2	NP_116171	-0.72	2.08E-05	1.18E-05	-9.02E-06	6.39E-03
LMAN2	NP_006807	-1.18	4.87E-05	3.50E-05	-1.36E-05	7.56E-03
KIAA1841	NP_001123465	-1.04	4.35E-05	2.68E-05	-1.67E-05	8.98E-03
CDK5RAP2	NP_060719	-0.23	4.41E-05	2.89E-05	-1.52E-05	9.06E-03
CRKL	NP_005198	-0.27	1.82E-05	1.50E-05	-3.17E-06	9.78E-03
HDAC1	NP_004955	-0.61	7.01E-04	5.89E-04	-1.12E-04	1.02E-02
CALD1	NP_149129	-0.24	4.01E-04	2.30E-04	-1.71E-04	1.13E-02
NLRC3	NP_849172	-0.28	2.54E-05	1.32E-05	-1.22E-05	1.16E-02
RPTN	NP_001116437	-0.41	3.03E-05	1.89E-05	-1.14E-05	1.57E-02
TRPS1	NP_054831	-0.92	6.73E-05	5.49E-05	-1.24E-05	1.60E-02
PDZRN3	NP_055824	-0.38	1.62E-05	1.11E-05	-5.11E-06	1.66E-02
VAT1	NP_006364	-0.39	3.54E-04	2.80E-04	-7.40E-05	1.67E-02
TAF15	NP_631961	-0.92	2.13E-05	1.39E-05	-7.48E-06	1.86E-02
TJAP1	NP_001139489	-0.35	1.13E-05	7.93E-06	-3.34E-06	1.88E-02
ERG	NP_001129626	-0.25	2.57E-05	1.33E-05	-1.24E-05	1.92E-02
DPYSL3	NP_001378	-0.70	4.92E-05	4.25E-05	-6.66E-06	2.04E-02
PLIN1	NP_001138783	-0.55	5.44E-06	3.29E-06	-2.16E-06	2.14E-02
ADCY4	NP_640340	-0.21	5.82E-05	3.42E-05	-2.39E-05	2.46E-02
ANKS1B	NP_690001	-0.35	2.50E-04	2.06E-04	-4.33E-05	2.51E-02
PPFIA4	NP_055868	-0.71	6.09E-06	3.38E-06	-2.70E-06	2.57E-02
DNAH5	NP_001360	-1.06	1.68E-04	1.00E-04	-6.79E-05	2.57E-02
CYP2B6	NP_000758	-0.48	1.25E-04	1.02E-04	-2.37E-05	2.64E-02
PPARGC1B	NP_001166170	-0.29	4.56E-04	2.20E-04	-2.36E-04	2.76E-02
FGD6	NP_060821	-0.29	2.34E-04	1.85E-04	-4.90E-05	2.86E-02
INTS4	NP_291025	-0.26	7.03E-05	4.90E-05	-2.12E-05	2.87E-02
GALM	NP_620156	-1.00	4.36E-05	2.69E-05	-1.67E-05	2.95E-02
PCOLCE	NP_002584	-0.39	2.64E-05	1.90E-05	-7.33E-06	3.01E-02
CPNE1	NP_690904	-0.34	2.21E-05	1.71E-05	-4.99E-06	3.47E-02
NCSTN	NP_056146	-0.35	3.96E-06	2.91E-06	-1.05E-06	3.85E-02
C12orf54	NP_689532	-0.27	1.44E-05	5.22E-06	-9.19E-06	3.87E-02
EBPL	NP_115954	-0.66	1.71E-04	1.13E-04	-5.88E-05	4.00E-02
BEST2	NP_060152	-0.37	3.37E-05	2.02E-05	-1.35E-05	4.13E-02
TBC1D22B	NP_060242	-0.58	4.74E-05	2.69E-05	-2.05E-05	4.53E-02
SKI	NP_003027	-0.27	1.92E-05	1.43E-05	-4.92E-06	4.56E-02

let-7a-2-3p (MIMAT0010195)						
**Gene Symbol**	**RefSeq ID**	**mirSVR Score**	**SR**	**SEN**	**Diff Mean**	***P*-value**
YWHAH	NP_003396	-0.58	6.61E-05	1.92E-05	-4.68E-05	8.56E-06
PLCB2	NP_004564	-0.46	1.96E-05	1.38E-05	-5.79E-06	1.27E-05
DNAJA2	NP_005871	-0.32	5.65E-06	3.04E-06	-2.61E-06	2.88E-05
CASS4	NP_065089	-0.86	3.14E-05	2.08E-05	-1.06E-05	4.54E-05
PCSK6	NP_612193	-0.77	3.60E-05	1.05E-05	-2.55E-05	6.86E-05
ELF1	NP_001138825	-0.21	5.78E-05	1.47E-05	-4.31E-05	1.24E-04
COL10A1	NP_000484	-0.22	1.90E-05	1.02E-05	-8.82E-06	1.39E-04
ANXA7	NP_001147	-0.26	3.71E-05	7.51E-06	-2.96E-05	1.93E-04
ZEB2	NP_055610	-0.65	3.21E-05	8.87E-06	-2.32E-05	5.33E-04
LRBA	NP_006717	-0.70	5.36E-05	3.31E-05	-2.05E-05	5.55E-04
DST	NP_056363	-0.28	4.49E-05	2.06E-05	-2.43E-05	6.40E-04
AHNAK	NP_001611	-1.14	2.98E-03	2.02E-03	-9.63E-04	7.28E-04
DNM3	NP_001129599	-0.38	3.87E-05	2.77E-05	-1.09E-05	7.91E-04
NAPA	NP_003818	-0.48	6.33E-04	4.23E-04	-2.10E-04	9.95E-04
CDH11	NP_001788	-0.81	1.18E-05	5.32E-06	-6.52E-06	1.05E-03
TCTEX1D1	NP_689878	-0.64	1.36E-05	5.81E-06	-7.81E-06	1.10E-03
ESR1	NP_000116	-0.21	3.86E-05	1.47E-05	-2.39E-05	1.11E-03
KTN1	NP_001072989	-0.30	2.25E-04	1.51E-04	-7.40E-05	1.18E-03
ANTXR1	NP_444262	-0.28	1.15E-05	5.19E-06	-6.26E-06	1.19E-03
CS	NP_004068	-1.16	1.49E-04	8.20E-05	-6.70E-05	1.20E-03
KLHL7	NP_001165899	-0.94	2.59E-05	1.32E-05	-1.27E-05	1.24E-03
UBA6	NP_060697	-0.23	2.59E-05	1.32E-05	-1.27E-05	1.24E-03
THAP11	NP_065190	-1.34	5.92E-05	3.28E-05	-2.63E-05	1.30E-03
LRCH4	NP_002310	-0.46	4.52E-05	2.59E-05	-1.93E-05	1.30E-03
RB1	NP_000312	-1.17	3.99E-04	2.18E-04	-1.81E-04	1.56E-03
GRB10	NP_005302	-0.72	1.86E-05	9.90E-06	-8.73E-06	1.62E-03
HN1	NP_057269	-0.98	1.86E-05	9.90E-06	-8.73E-06	1.64E-03
NCBP1	NP_002477	-0.30	1.11E-03	5.55E-04	-5.51E-04	1.75E-03
PLEKHA5	NP_001137293	-0.57	3.64E-04	2.05E-04	-1.60E-04	1.76E-03
H1F0	NP_005309	-0.63	6.34E-03	2.55E-03	-3.78E-03	1.93E-03
DYNC1LI2	NP_006132	-0.53	2.42E-05	1.46E-05	-9.64E-06	2.14E-03
ZNF10	NP_056209	-0.59	3.19E-05	1.85E-05	-1.34E-05	2.40E-03
RC3H2	NP_001094058	-0.23	2.38E-04	9.35E-05	-1.44E-04	2.44E-03
GTF2IRD1	NP_005676	-0.77	2.30E-05	4.49E-06	-1.85E-05	2.46E-03
COL3A1	NP_000081	-0.81	6.74E-04	4.16E-04	-2.58E-04	2.49E-03
ZC3H13	NP_055885	-1.04	8.71E-06	3.87E-06	-4.84E-06	2.54E-03
HNRNPK	NP_112553	-0.26	3.12E-04	1.97E-04	-1.15E-04	2.68E-03
MECOM	NP_001098547	-0.34	2.44E-05	1.48E-05	-9.65E-06	2.74E-03

FAM178A	NP_001129595	-0.21	3.30E-05	2.15E-05	-1.15E-05	2.92E-03
ARID1B	NP_059989	-0.53	2.15E-04	1.20E-04	-9.52E-05	2.93E-03
HIVEP2	NP_006725	-0.55	1.31E-04	8.79E-05	-4.35E-05	3.01E-03
ANKRD11	NP_037407	-0.81	4.89E-05	3.12E-05	-1.78E-05	3.56E-03
SP140	NP_009168	-0.30	2.28E-05	1.50E-05	-7.79E-06	3.63E-03
SRP72	NP_008878	-0.31	2.90E-05	2.02E-05	-8.86E-06	3.80E-03
ARHGAP36	NP_659404	-0.95	7.17E-05	5.47E-05	-1.70E-05	3.92E-03
RBBP6	NP_008841	-0.33	2.61E-05	1.61E-05	-1.00E-05	4.07E-03
CCDC88A	NP_001129069	-0.39	9.08E-05	6.46E-05	-2.62E-05	4.77E-03
PSIP1	NP_001121689	-0.40	1.86E-05	1.20E-05	-6.55E-06	4.77E-03
PKP4	NP_003619	-0.25	3.67E-04	2.64E-04	-1.03E-04	5.07E-03
HNRNPH2	NP_062543	-0.83	3.11E-05	1.32E-05	-1.79E-05	5.38E-03
METTL6	NP_689609	-0.38	3.63E-05	2.65E-05	-9.81E-06	5.44E-03
PPM1K	NP_689755	-0.27	1.85E-05	1.20E-05	-6.48E-06	5.71E-03
AKAP12	NP_005091	-0.51	1.08E-04	7.54E-05	-3.26E-05	5.86E-03
STAC2	NP_945344	-0.23	1.84E-05	1.33E-05	-5.12E-06	6.01E-03
NPVF	NP_071433	-0.78	2.44E-05	1.16E-05	-1.28E-05	6.07E-03
RPS6KA5	NP_004746	-0.40	5.24E-05	1.51E-05	-3.73E-05	6.19E-03
SUZ12	NP_056170	-0.38	2.65E-05	1.54E-05	-1.12E-05	6.65E-03
EIF4ENIF1	NP_062817	-1.14	7.04E-05	4.89E-05	-2.15E-05	7.11E-03
MMRN2	NP_079032	-0.87	1.06E-05	3.89E-06	-6.70E-06	7.43E-03
TRIO	NP_009049	-0.78	3.60E-05	1.71E-05	-1.89E-05	7.69E-03
FLG2	NP_001014364	-1.24	1.98E-05	1.42E-05	-5.58E-06	7.83E-03
PAFAH1B2	NP_002563	-0.88	2.13E-05	1.48E-05	-6.49E-06	8.43E-03
TPM3	NP_705935	-0.89	7.60E-05	5.26E-05	-2.34E-05	8.49E-03
SEC24D	NP_055637	-0.44	3.76E-05	2.56E-05	-1.21E-05	8.52E-03
DOCK8	NP_001177387	-0.37	8.39E-06	2.33E-06	-6.06E-06	8.83E-03
KIAA1841	NP_001123465	-1.24	4.35E-05	2.68E-05	-1.67E-05	8.98E-03
ITSN1	NP_001001132	-0.80	1.95E-05	7.95E-06	-1.15E-05	9.45E-03
MED13L	NP_056150	-0.99	8.49E-05	4.77E-05	-3.72E-05	9.63E-03
RAB5B	NP_002859	-0.37	4.41E-04	3.03E-04	-1.38E-04	1.03E-02
ZNF518A	NP_055618	-0.93	9.54E-04	4.97E-04	-4.56E-04	1.04E-02
KIDINS220	NP_065789	-0.31	4.02E-05	2.65E-05	-1.36E-05	1.12E-02
SYT13	NP_065877	-0.52	1.06E-04	4.56E-05	-6.00E-05	1.12E-02
CALD1	NP_149129	-0.23	4.01E-04	2.30E-04	-1.71E-04	1.13E-02
ATP8A1	NP_006086	-0.22	1.06E-04	4.58E-05	-5.98E-05	1.14E-02
IKBIP	NP_710154	-0.38	1.08E-04	6.84E-05	-3.96E-05	1.15E-02
UBE2F	NP_542409	-0.68	1.33E-05	5.30E-06	-7.98E-06	1.33E-02
SORL1	NP_003096	-0.25	3.49E-05	2.19E-05	-1.29E-05	1.35E-02
IFRD1	NP_001541	-0.45	1.05E-04	8.19E-05	-2.34E-05	1.57E-02
AKAP1	NP_003479	-0.97	2.57E-05	1.36E-05	-1.20E-05	1.57E-02
TRPS1	NP_054831	-0.37	6.73E-05	5.49E-05	-1.24E-05	1.60E-02
RALGAPA1	NP_055805	-0.66	6.20E-05	4.65E-05	-1.55E-05	1.67E-02

TMEM131	NP_056163	-0.77	3.41E-05	2.31E-05	-1.10E-05	1.68E-02
RNF141	NP_057506	-0.57	2.10E-04	1.25E-04	-8.45E-05	1.77E-02
BTF3	NP_001032726	-1.01	1.83E-05	1.47E-05	-3.53E-06	1.80E-02
HNRNPA2B1	NP_112533	-0.41	1.33E-04	7.21E-05	-6.06E-05	1.84E-02
SULF1	NP_055985	-1.07	1.11E-05	6.48E-06	-4.57E-06	1.85E-02
SMARCA5	NP_003592	-1.28	2.66E-04	1.80E-04	-8.64E-05	1.89E-02
S100A6	NP_055439	-0.24	7.28E-06	4.17E-06	-3.10E-06	1.92E-02
PICALM	NP_009097	-1.03	3.28E-05	1.87E-05	-1.41E-05	2.12E-02
ZNHIT6	NP_060423	-0.39	7.97E-05	1.50E-05	-6.48E-05	2.20E-02
NAP1L1	NP_004528	-0.53	1.93E-05	8.63E-06	-1.07E-05	2.35E-02
ANP32B	NP_006392	-1.34	1.17E-05	8.69E-06	-2.99E-06	2.37E-02
PSME4	NP_055429	-0.42	1.37E-05	9.08E-06	-4.58E-06	2.41E-02
NAA50	NP_079422	-0.47	3.77E-05	2.07E-05	-1.71E-05	2.43E-02
HKDC1	NP_079406	-0.30	1.14E-05	3.39E-06	-8.06E-06	2.48E-02
GAK	NP_005246	-0.74	6.45E-05	2.78E-05	-3.67E-05	2.63E-02
CORO1C	NP_055140	-0.77	4.36E-04	2.07E-04	-2.29E-04	2.63E-02
CYP2B6	NP_000758	-0.37	1.25E-04	1.02E-04	-2.37E-05	2.64E-02
NODAL	NP_060525	-0.79	1.28E-05	9.55E-06	-3.27E-06	2.68E-02
C5orf34	NP_940968	-0.29	1.59E-04	5.98E-05	-9.92E-05	2.72E-02
INVS	NP_899068	-0.61	6.32E-05	4.35E-05	-1.97E-05	2.73E-02
ANO5	NP_001136121	-0.44	5.54E-06	2.55E-06	-2.99E-06	2.91E-02
HOXA1	NP_005513	-1.07	2.82E-06	1.53E-06	-1.30E-06	3.08E-02
VANGL2	NP_065068	-1.31	2.13E-05	1.59E-05	-5.36E-06	3.10E-02
ARHGAP11A	NP_055598	-0.96	3.57E-05	2.67E-05	-9.02E-06	3.11E-02
SETDB2	NP_001153780	-1.11	9.39E-05	6.74E-05	-2.64E-05	3.37E-02
KIF5C	NP_004513	-0.71	3.74E-05	2.77E-05	-9.65E-06	3.40E-02
DOCK5	NP_079216	-0.43	1.15E-05	6.57E-06	-4.96E-06	3.42E-02
PCBP4	NP_065151	-0.78	2.36E-05	1.54E-05	-8.20E-06	3.43E-02
PSMC6	NP_002797	-0.27	1.59E-04	1.11E-04	-4.72E-05	3.45E-02
NID1	NP_002499	-0.86	2.41E-05	1.01E-05	-1.40E-05	3.46E-02
KIF23	NP_004847	-1.27	3.96E-06	1.79E-06	-2.17E-06	3.52E-02
BCL6	NP_001124317	-1.32	2.67E-05	2.01E-05	-6.50E-06	3.80E-02
ZEB1	NP_001121600	-1.05	7.96E-06	5.67E-06	-2.29E-06	3.82E-02
FKBP11	NP_001137254	-0.26	3.22E-05	2.04E-05	-1.17E-05	3.85E-02
FBXO38	NP_110420	-1.13	4.95E-05	3.38E-05	-1.56E-05	3.91E-02
RBM20	NP_001127835	-0.29	2.52E-05	1.78E-05	-7.44E-06	4.00E-02
FBXO25	NP_036305	-0.30	7.09E-05	5.34E-05	-1.75E-05	4.22E-02
ST18	NP_055497	-0.57	3.33E-05	1.47E-05	-1.86E-05	4.27E-02
RALYL	NP_001093861	-1.30	3.22E-05	1.78E-05	-1.45E-05	4.43E-02
MTM1	NP_000243	-0.48	5.43E-06	2.91E-06	-2.52E-06	4.47E-02
CENPBD1	NP_659476	-0.33	1.09E-05	8.18E-06	-2.70E-06	4.49E-02
TBC1D22B	NP_060242	-0.37	4.74E-05	2.69E-05	-2.05E-05	4.53E-02
PIP4K2C	NP_001139731	-0.28	4.63E-05	3.60E-05	-1.03E-05	4.54E-02

MAP4K3	NP_003609	-0.99	4.64E-05	3.07E-05	-1.57E-05	4.55E-02
PCDH15	NP_001136239	-1.15	1.09E-05	7.87E-06	-3.00E-06	4.72E-02
PRDM6	NP_001129711	-0.22	1.93E-04	1.51E-04	-4.26E-05	4.72E-02
DSCAML1	NP_065744	-0.75	1.45E-05	1.17E-05	-2.72E-06	4.84E-02
CEP350	NP_055625	-0.79	7.84E-05	6.00E-05	-1.84E-05	4.94E-02
HMGB1	NP_002119	-1.16	8.94E-06	5.10E-06	-3.84E-06	4.96E-02
PDCD6	NP_037364	-0.60	5.74E-06	3.26E-06	-2.48E-06	4.97E-02
