# Supplementary material for: Opposing activities of oncogenic MIR17HG and tumor suppressive MIR100HG clusters and their gene targets regulate replicative senescence in human adult stem cells
Source: NPJ Aging Mech Dis. 2017 Apr 20;3:7. doi: 10.1038/s41514-017-0006-y (PMC5460214; doi:10.1038/s41514-017-0006-y)
Supplement: Supplementary file 10 — Supplementary Figure9 [file 41514_2017_6_MOESM10_ESM.pdf]

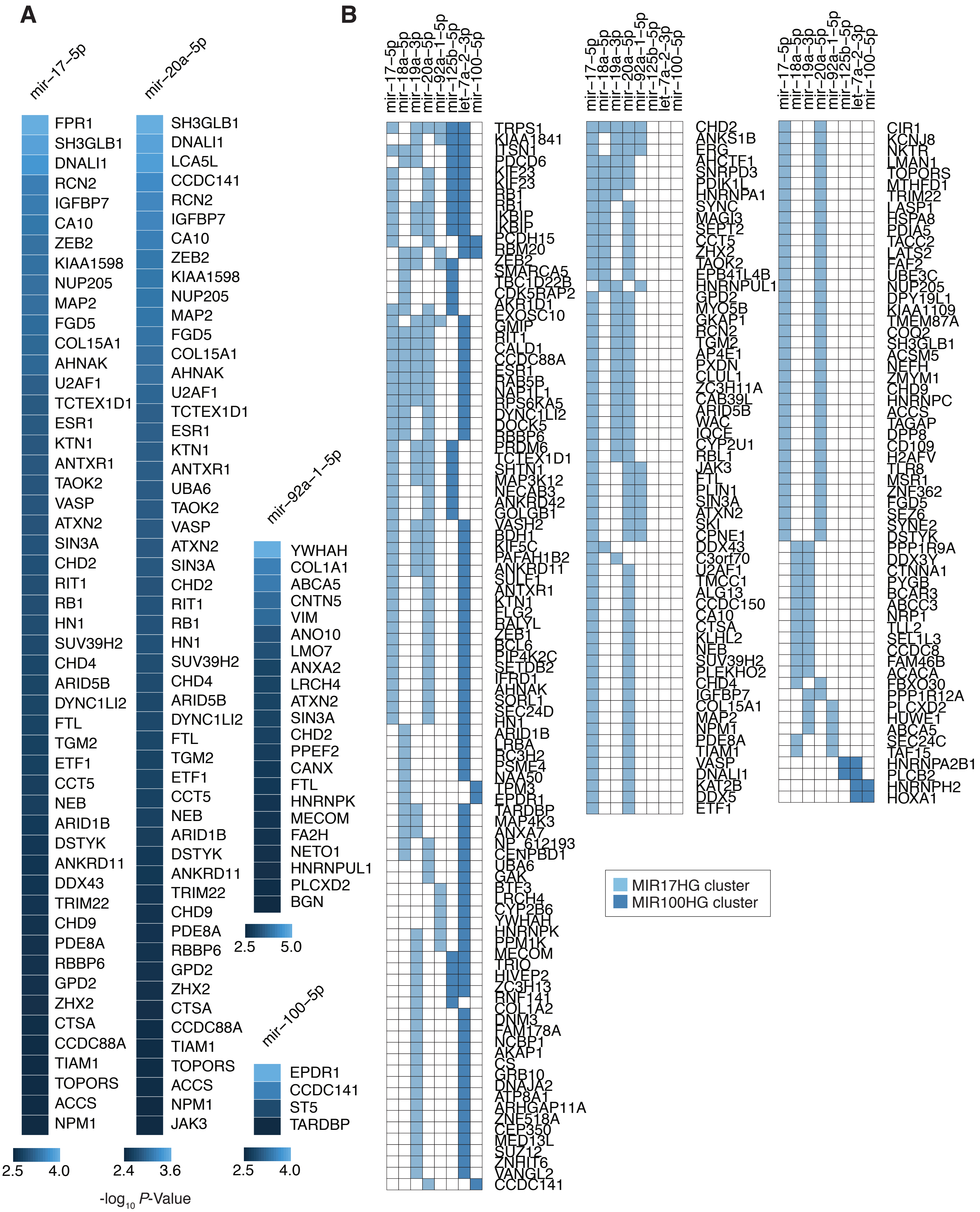

Heatmap for top 40% consistent targets

Suppl. Fig.9
